# Heritability of human “directed” functional connectome

**DOI:** 10.1002/brb3.2839

**Published:** 2023-03-29

**Authors:** Maria Giovanna Bianco, Andrea Duggento, Salvatore Nigro, Allegra Conti, Nicola Toschi, Luca Passamonti

**Affiliations:** ^1^ Neuroscience Research Center, Department of Medical and Surgical Sciences “Magna Graecia” University of Catanzaro Italy; ^2^ Department of Biomedicine and Prevention University “Tor Vergata” Rome Italy; ^3^ Institute of Nanotechnology (NANOTEC) National Research Council Lecce Italy; ^4^ Center for Neurodegenerative Diseases and the Aging Brain, Department of Clinical Research in Neurology University of Bari 'Aldo Moro, “Pia Fondazione Cardinale G. Panico” Tricase Italy; ^5^ Martinos Center for Biomedical Imaging Massachusetts General Hospital & Harvard Medical School Charlestown Boston MA 02129 USA; ^6^ Institute of Bioimaging and Molecular Physiology National Research Council Milan Italy; ^7^ Department of Clinical Neurosciences University of Cambridge Cambridge UK

**Keywords:** default mode network, fronto‐cerebellar networks, Granger causality, heritability, resting state fMRI

## Abstract

**Introduction:**

The functional connectivity patterns in the brain are highly heritable; however, it is unclear how genetic factors influence the directionality of such “information flows.” Studying the “directionality” of the brain functional connectivity and assessing how heritability modulates it can improve our understanding of the human connectome.

**Methods:**

Here, we investigated the heritability of “directed” functional connections using a state‐space formulation of Granger causality (GC), in conjunction with blind deconvolution methods accounting for local variability in the hemodynamic response function. Such GC implementation is ideal to explore the directionality of functional interactions across a large number of networks. Resting‐state functional magnetic resonance imaging data were drawn from the Human Connectome Project (total *n = 898* participants). To add robustness to our findings, the dataset was randomly split into a “discovery” and a “replication” sample (each with *n = 449* participants). The two cohorts were carefully matched in terms of demographic variables and other confounding factors (e.g., education). The effect of shared environment was also modeled.

**Results:**

The parieto‐ and prefronto‐cerebellar, parieto‐prefrontal, and posterior‐cingulate to hippocampus connections showed the highest and most replicable heritability effects with little influence by shared environment. In contrast, shared environmental factors significantly affected the visuo‐parietal and sensory‐motor directed connectivity.

**Conclusion:**

We suggest a robust role of heritability in influencing the directed connectivity of some cortico‐subcortical circuits implicated in cognition. Further studies, for example using task‐based fMRI and GC, are warranted to confirm the asymmetric effects of genetic factors on the functional connectivity within cognitive networks and their role in supporting executive functions and learning.

## Graphical abstract

We investigated the heritability of ‘directed’ functional connections using a state‐space formulation of Granger causality on resting‐state functional magnetic resonance imaging data from the Human Connectome Project. The parieto‐cerebellar, parieto‐prefrontal, and posterior‐cingulate to hippocampus connections showed the highest and most replicable heritability effects with little influence by the shared environment. In contrast, shared environmental factors significantly affected the visuo‐parietal and sensory‐motor directed connectivity.

We suggest a robust role of heritability in influencing the function of some cortico‐subcortical circuits consistently implicated in cognition.

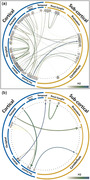

## 1 INTRODUCTION

Heritability is defined as the degree of variance in a biological trait that can be explained by shared genetic factors (Glahn et al., 2010). To quantify heritability, classic twin studies and statistical approaches applied to large groups of unrelated individuals have been used (Ge et al., 2015, 2016; Golan et al., 2014; Polderman et al., 2015; Wedel, 1962; Yang et al., 2010, 2011) (Almasy & Blangero, 1998).

Recently, there has been a growing interest in studying the heritability of brain functional connectivity “at rest” (Abbasi et al., 2020; Adhikari et al., 2018; Elliott et al., 2018, 2019; Reineberg et al., 2020). To this end, numerous methods have been developed, spanning from bivariate correlational analyses to “connectotyping” methods based on machine learning (Colclough et al., 2017; Elliott et al., 2019; Ge et al., 2017; Glahn et al., 2010; Miranda‐Dominguez et al., 2017a; Sinclair et al., 2015a; Yang et al., 2016). The findings reported have been mixed, although there is general consensus that the functional connectivity patterns involving the default mode network (DMN) are highly heritable (Colclough et al., 2017; Ge et al., 2017; Glahn et al., 2010; Miranda‐Dominguez et al., 2017b; Sinclair et al., 2015b; Yang et al., 2016).

The directionality of these genetic effects remains, however, largely uncharacterized. Assessing the direction of the “information flow” in the brain and how this is influenced by hereditary factors can provide mechanistic insights to understand the functional connectome. The brain functional connectivity patterns typically derive from “bottom‐up” interactions (e.g., from lower sensory areas to progressively higher order regions) and “top‐down” communication patterns (vice versa). These specific and “directed” connections support distinct aspects of information processing, cognition, and behavior. One cannot simply assume that the functional interactions in the human connectome are symmetrical, despite most of the anatomical connections being bi‐directional. Several genes can also influence brain functioning in different ways, and some of these effects may be more pronounced in certain “directed” pathways than others.

Past research has explored the directed connectivity patterns in the human brain using different methods (Bajaj et al., 2016; Chén et al., 2019; Duggento et al., 2018; Lund et al., 2020; Schwab et al., 2018; Xu et al., 2020), although no one has studied how heritability influences the “information flow” from a functional network to another one. The aim of the present study is to characterize the heritability of the human “directed” functional connectome. To this end, we employed high‐quality resting‐state functional magnetic resonance imaging (rs‐fMRI) data from the Human Connectome Project (HCP) (S1200 data release).

**FIGURE 1:**
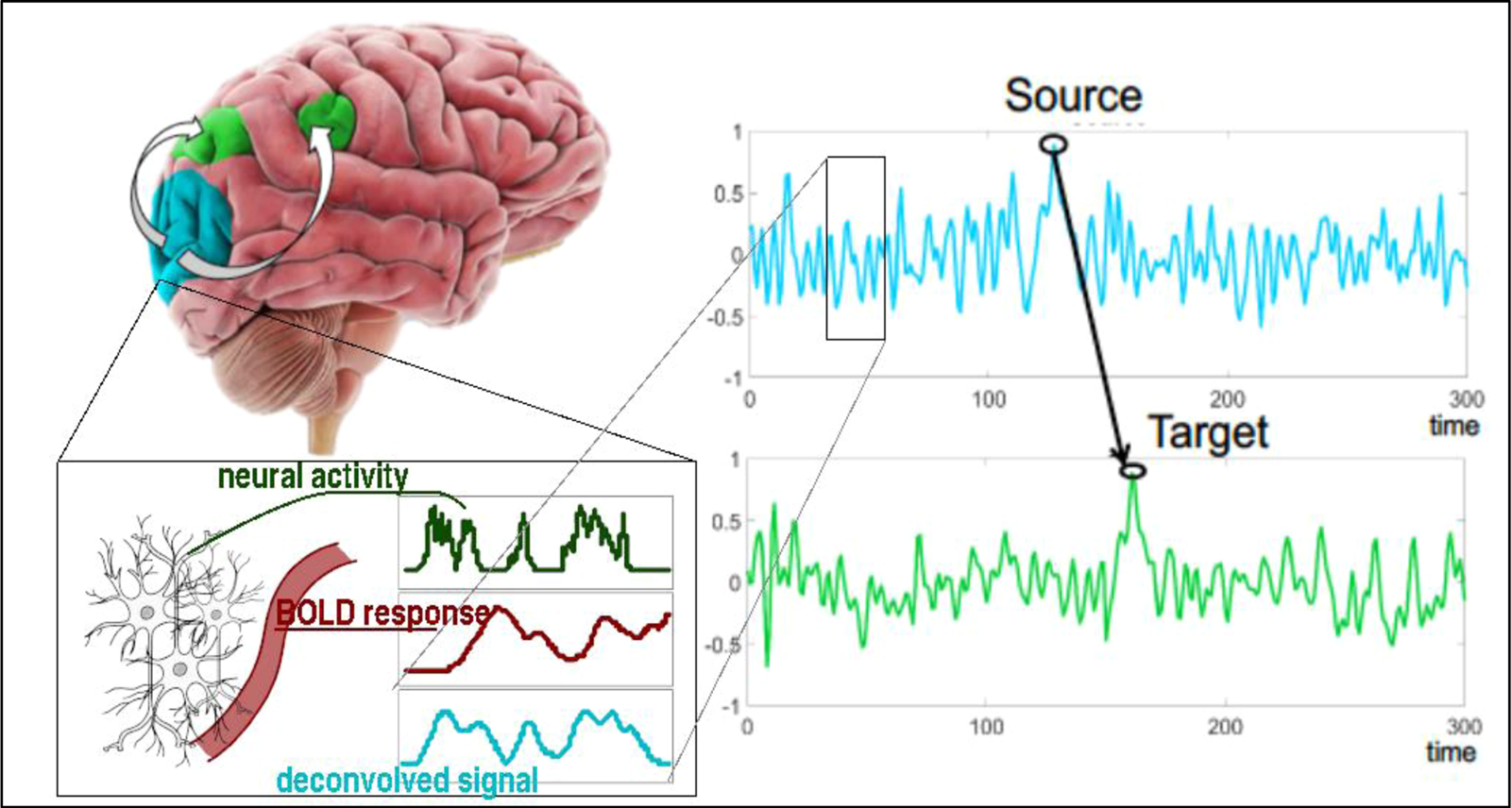
Effective connectivity estimation. Granger causality (GC) methods infer causal influences between the estimated activity in a specific brain region or network (“Target”) on the basis of the information contained in the “past” of the estimated activity in another region or network (“Source”). The estimated activity in the “Target” and “Source” is inferred from the BOLD signal via “blind” deconvolution methods (see Section 2.4).

To estimate the directed connectome, we used Granger causality (GC), in its most recent state‐space formulation. GC is a commonly employed analytical technique which is based on the concept of predictability (Figure 1) (Seth et al., 2015). To control for non‐uniform delays when estimating the neuronal activity from the BOLD signal (Handwerker et al., 2012), we applied a “blind” deconvolution technique, which infers both the shape of the hemodynamic response function (HRF) and its underlying neural activity (Wu et al., 2013).

To study the heritability of GC measures, we calculated the extent to which inter‐subject GC became more similar as a function of the relatedness between individuals. We approached this through a maximum likelihood variance decomposition method of family‐based quantitative data (Amos, 1994). This method decomposes the “phenotypic” variance of GC into a genetic and an environmental component, as well as their interaction.

To strengthen our findings, we randomly split the HCP dataset into two cohorts (herein called the “discovery” and “replication” sample) and carefully matched them in terms of age, gender, education, and handedness (Adhikari et al., 2018; Fairchild et al., 2016). We aimed to investigate inter‐subject variability with two independent samples to verify whether the findings in one cohort were replicated in a second cohort. Kinship was taken into account while splitting the datasets, to prevent that twin couples were divided between the discovery and replication dataset. We also assessed the impact of the shared environment on our findings. For completeness, we report the findings in both cohorts, although we focus our discussion on the results that replicated between them (i.e., 38% of the total heritable connections and 14% taking in account the shared environment).

To the best of our knowledge, this study is the first to characterize the heritability of the “directed” functional connectome. Due to the exploratory nature of our work, we had no a priori hypothesis regarding the directionality of the effects. Nevertheless, the existing literature suggested a high level of heritability in the connections originating from and/or “directed” towards the DMN (Ge et al., 2017; Glahn et al., 2010; Sinclair et al., 2015a; Yang et al., 2016).

## 2 MATERIALS AND METHODS

### 2.1 Participants

We employed data drawn from the HCP (http://www.humanconnectome.org/, *n* = 1206 individuals). The rs‐fMRI scans were released in March, 2017 (humanconnectome.org) after passing the quality control and assurance standards set up by the HCP consortium (Marcus et al., 2013). *N* = 203 individuals were removed from our analyses due to the lack of rs‐fMRI data. We randomly divided the remaining sample (*n* = 1003) into a discovery and a replication cohort that were carefully matched for sex distribution, age, education, handedness, and kinship (i.e., number of monozygotic [MZ] or dizygotic [DZ] twins, non‐twin siblings, and singletons). The years of education were used as a proxy index of education level and indirect measure of potential differences in familiar socioeconomic status. See Table 1 for general demographic characteristics of the samples and Table S1 for further details. The matching was performed using MedCalc statistical software and resulted in the exclusion of *n* = 107 participants to achieve sufficient matching quality (https://www.medcalc.org/). The final population included *n* = 898 people divided into a discovery and replication sample of *n* = 449 individuals each, composed as follows: *n* = 124 MZ twins, *n* = 50 DZ twins, *n* = 197 non‐twin siblings, and *n* = 78 singletons.

**TABLE 1:** Demographic variables in the discovery and replication set

**Demographic variables**				
*Discovery set*				
	Gender (males/females)	Age (years)	Handedness (right/left/both)	Education (years)
MZ	52/72	29.0 ± 3.3	118/6/0	15.1 ± 1.8
DZ	22/28	29.5 ± 3.5	45/5/0	15.2 ± 1.7
Siblings	103/94	28.4 ± 3.8	183/14/0	15.0 ± 1.7
Singletons	37/41	29.1 ± 4.1	67/11/0	14.7 ± 1.8
*Replication set*				
	Gender (males/females)	Age (years)	Handedness (right/left/both)	Education (years)
MZ	52/72	29.3 ± 3.4	110/14/0	14.8 ± 1.9
DZ	22/28	29.5 ± 3.5	48/2/0	15.1 ± 1.7
Siblings	103/94	28.1 ± 3.8	178/16/3	15.0 ± 1.7
Singletons	37/41	29.0 ± 3.8	71/7/0	14.8 ± 1.8

### 2.2 MRI scanning protocol and preprocessing

In each participant, four rs‐fMRI scans were acquired via a customized 67 Siemens Skyra 3T scanner (Van Essen et al., 2013) with 1200 timepoints/scan, TR/TE = 720/33.1 ms, FA = 52°, FOV = 208 × 180; 72 slices; 2.0 mm isotropic voxel size, multiband factor 8, Echo spacing = .58 ms, BW = 2290 Hz/Px, and 14 min of acquisition time/scan. The pre‐processing pipeline included artifact correction through an automatic classifier trained on the HCP dataset (ICA+FIX) (Griffanti et al., 2014; Salimi‐Khorshidi et al., 2014), which has >99% specificity and sensitivity in recognizing and removing artifacts, including physiological noise. Further information about preprocessing of rs‐fMRI data is available at (https://www.humanconnectome.org/storage/app/media/documentation/s1200/HCP_S1200_Release76_Reference_Manual.pdf). After pre‐processing, a group‐principal component analysis was run across all subjects and fed into group‐wise spatial independent component analysis (ICA) using FSL MELODIC tool to obtain 100 distinct spatio‐temporal components (Jenkinson et al., 2012). Henceforth, these components will be termed “ICA networks.” For each subject, we analyzed 400 signals (100 ICA‐networks x 4 sessions) with a length of 1200 time‐points each. The above described MRI scanning protocol and preprocessing were performed by the HCP consortium prior to data release (https://www.humanconnectome.org/study/hcp‐young‐adult/article/release‐s1200‐extensively‐processed‐rfmri‐data).

### 2.3 “Blind” deconvolution and effective connectivity estimation

To remove the temporal and spatial confound related to locally varying HRF, each signal underwent blind deconvolution via an algorithm specifically designed for rs‐fMRI data (Wu et al., 2013). Next, each BOLD signal was deconvolved using the corresponding HRF estimate which produced a more accurate proxy of the underlying neuronal activity. This procedure augments the accuracy of resting‐state derived connectivity measures in a variety of contexts (Rangaprakash et al., 2018; Wu et al., 2019) (See Figure 1 for a pictorial representation).

After deconvolution, multivariate between‐network effective connectivity was estimated via the most recent formulation of state‐space GC (Barnett & Seth, 2015). State‐space GC enables a reliable GC evaluation because it relies on few mathematical assumptions about the nature of the data. In detail, it allows to relax assumptions of linearity, stationarity, and homoscedasticity of the signals. To this end, we employed a publicly available Matlab tool (http://www.lucafaes.net/msGC.html) detailed in Faes et al. (2017), which was modified in‐house to employ, for each subject, all four sessions‐specific timeseries within the same model. This way, we pooled within‐subject measures into a single model estimation, yielding higher robustness (Figure 1). From the evaluation of state‐space GC, in each subject, we obtained the connection strengths between any two ICA‐networks—which resulted in 1003, 100 × 100 non‐symmetric matrices. To evaluate the global directed connectome, for each connection, the median strength among all subjects was calculated.

### 2.4 Quantitative genetic analyses

The heritability of GC connectivity was investigated using the Sequential Oligogenic Linkage Analysis Routines (SOLAR)‐Eclipse software (http://www.nitrc.org/projects/se_linux). SOLAR implements variance component models on family‐based quantitative data and fits such models using the maximum likelihood estimation (Amos, 1994). SOLAR handles pedigrees (i.e., dataset containing genetic relationships between family members) of arbitrary size and complexity and it calculates heritability values, genetic correlations, linkage. SOLAR also performs genome‐wide association analyses with asymptotically precise estimates (Almasy & Blangero, 1998; Blangero et al., 2001). In this framework, the phenotypic trait variability that exists in a population, or phenotypic variance (*σ_P_* ^2^), is decomposed to estimate how much a variation between individuals results from genetic (*σ* ^2^ *_g_*) or environmental differences (*σ* ^2^ *_e_*):

(1) $$\sigma_{p}^{2}=\sigma_{g}^{2}\;+\sigma_{e}^{2}.$$

This model treats each functional connection between ICA networks as a trait and the phenotypic covariance matrix for a pedigree among family members is modeled as a function of genetic kinship as:

(2) $$\Omega =2\Phi \sigma_{g}^{2}+I\sigma_{e}^{2},$$

where Ω is a matrix of size *n x m*, *n* is the number of individuals in the pedigree for whom trait measurement is available, *m* is the number of functional connections. *σ* ^2^ *_g_* is the total additive genetic variance, *σ* ^2^ *_e_* is the variance due to environmental influences, *Φ* is the kinship matrix (i.e., the pair‐wise kinship coefficients that identify related individuals), and *I* is an identity matrix (which assumes that all environmental effects are uncorrelated among family members) (Almasy & Blangero, 1998).

The heritability measures how much a trait variation is due to genetic effects. It ranges from zero (when a trait is fully driven by environmental factors) to one, when genetics fully explains trait's variability.

Narrow sense heritability (*h* ^2^), that is, a measure of the strength of genetic effects on a specific trait, is defined as the ratio between the additive genetic effects (*σ_g_* ^2^) and total phenotypic variance (*σ_P_* ^2^):

(3) $$h^{2}=\sigma_{g}^{2}\;/\sigma_{p}^{2}.$$

*h* ^2^ reflects the shared genotypic variation of traits and is higher when more individuals have stronger genetic heritage.

In twin designs, a third variance parameter can be modelled (*σ_c_* ) to account for the shared environment of individuals growing up in the same family. This three‐parameter model is known as the ACE model, while the two‐parameter model (2) is called AE model.

The heritability due to genetic causes (A) is the ratio of $\sigma_{g}^{2}$ and $\sigma_{p}^{2}$ with:

(4) $$\sigma_{p}^{2}\approx \sigma_{g}^{2}+\sigma_{c}^{2}+\sigma_{e}^{2}.$$

Under these assumptions, C is the ratio of the variance contributed by common environmental variance to total variance and E is the ratio of the variance due to unique environmental effects and measurement error to total variance, following the ACE model of heritability.

We evaluated the heritability of GC functional connectivity considering each connection as an independent brain “endophenotypic” trait. In the discovery and replication group, age, sex, handedness, and education were included as covariates of no interest hence adjusting connectivity‐values for each individual before evaluating heritability. Moreover, inverse Gaussian transformation was applied to functional connectivity values to ensure normality of measurements.

We estimated a three‐parameter model for each traits, heritability, and sample size combination using the “polygenic” command. The effect of shared environment for people with a first‐degree kinship was also modeled. As the HCP consortium does not provide household information, we assumed that two individuals shared the same household if they had the same family id, using the SOLAR option “‐keephouse.”

**FIGURE 2:**
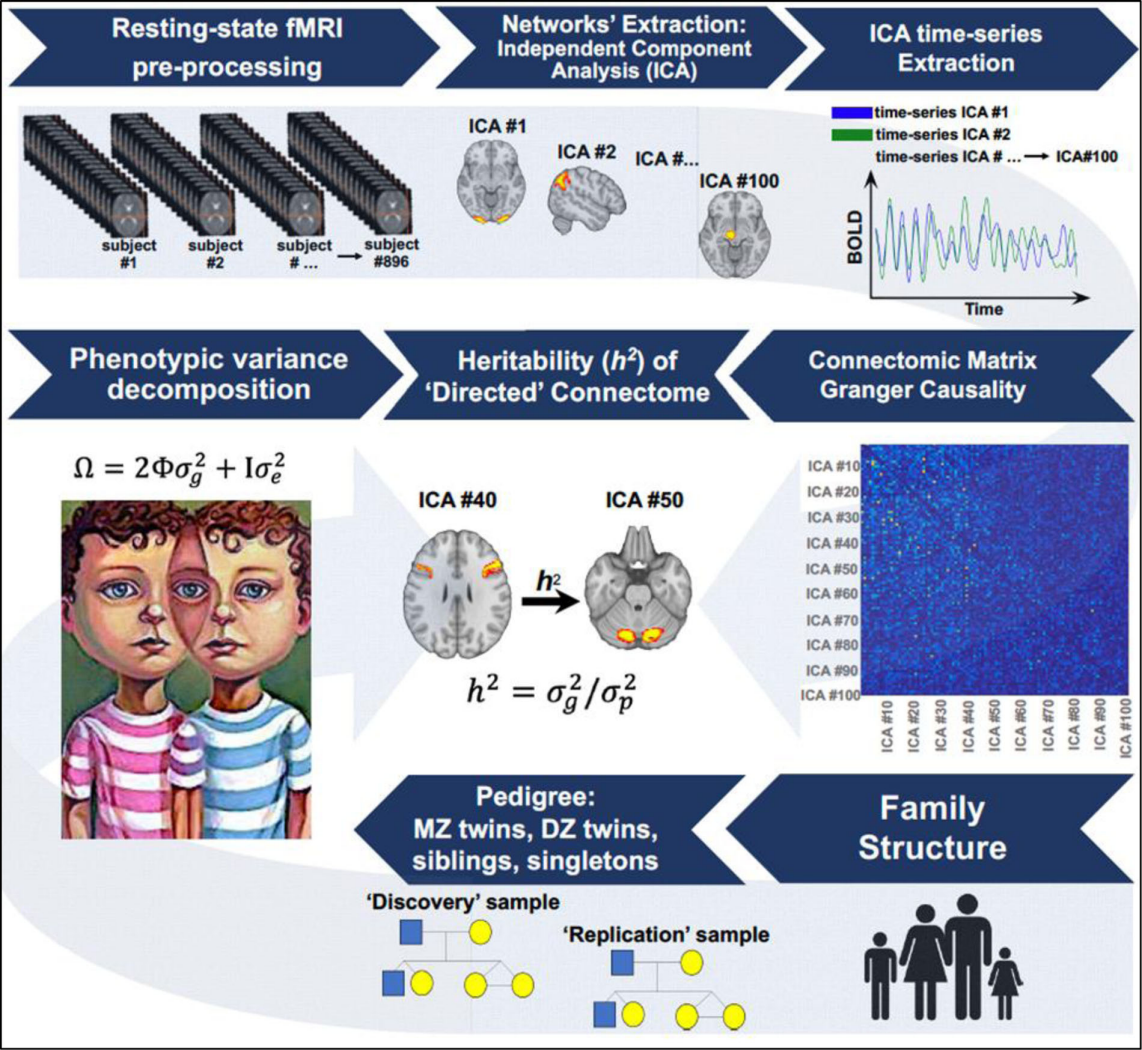
Image analysis workflow. Resting‐state functional magnetic imaging (fMRI) data were pre‐processed by the HCP consortium, including the extraction of *n* = 100 brain networks and corresponding time‐series via independent components analysis (ICA). A “blind” deconvolution approach eliminated confounds related to local HRF variations while the subject‐specific and directed functional connectivity matrices were calculated using state‐space Granger causality. For each family, kinship information was organized into a pedigree and phenotypic matrix that was derived considering the functional connectivity ICA as traits. Finally, the variance components method, as implemented in the Sequential Oligogenic Linkage Analysis Routines (SOLAR) software, was used to estimate the heritability of directed functional connectivity between brain networks.

An *h* ^2^ value was considered statistically significant when *p* < .05, using a false discovery rate (FDR) correction for multiple comparisons among functional connectivity ICA. The image analysis pipeline is summarized in Figure 2. The heritability of functional connections was evaluated separately for discovery and replication set.

## 3 RESULTS

### 3.1 Participants

The demographic characteristics of the participants in the discovery and replication sample are summarized in Table 1. Our matching procedures ensured that no significant differences were found between the discovery and replication set in terms of sex distribution, age, education, handedness, and kinship (Table S1 for the statistical details).

### 3.2 Neuroimaging findings

#### 3.2.1 ICA of networks “nodes”

**FIGURE 3:**
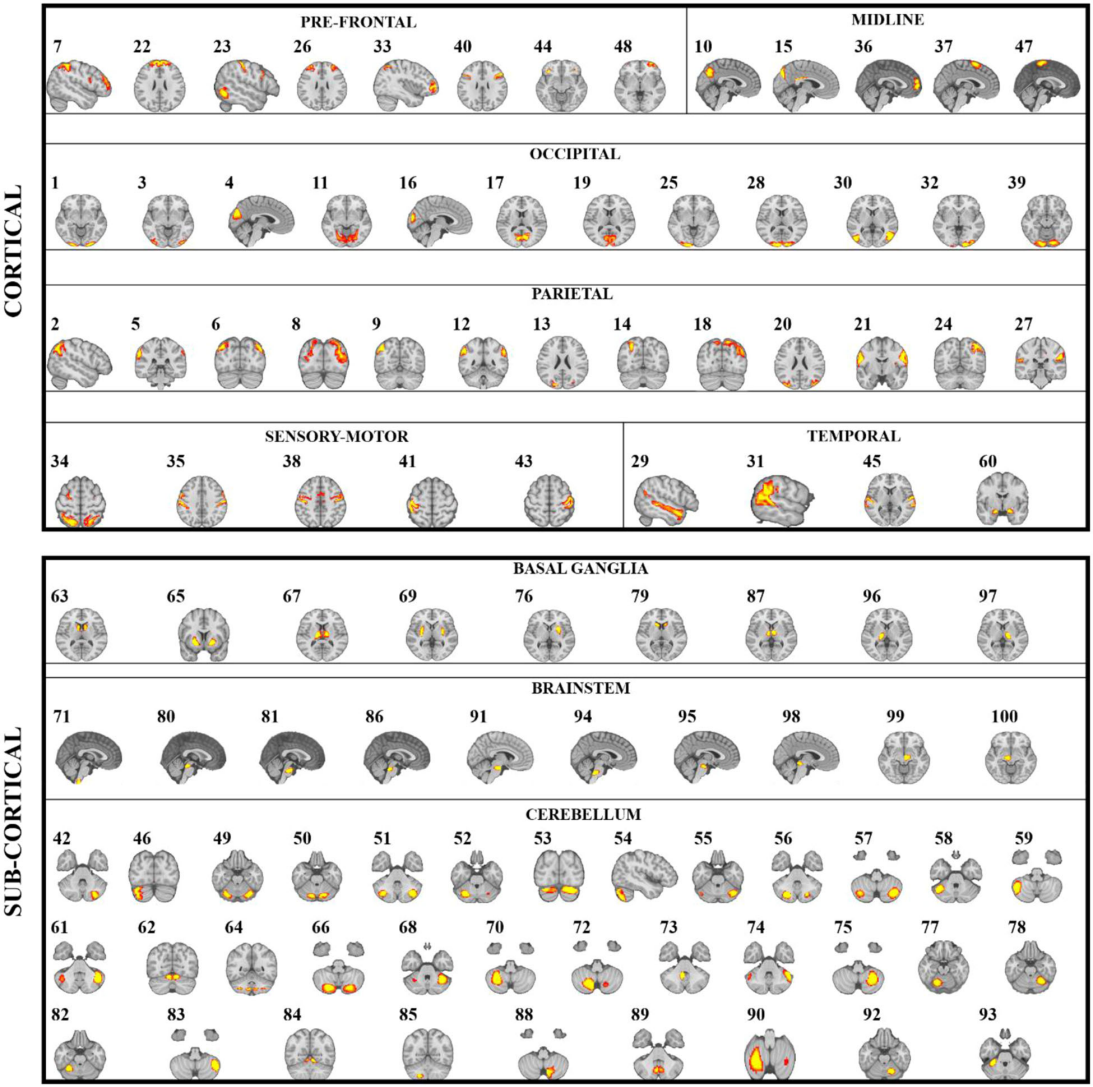
Brain functional networks (i.e., independent components). These networks were identified by group independent component analysis in *n* = 1003 subjects drawn from the Human Connectome Project database.

Each ICA network (i.e., “node”) was characterized by a set of brain regions that are consistent with previous studies and different levels of “granularity” in resting‐state networks (Toschi et al., 2017) (Figure 3).

#### 3.2.2 Global directed functional connectome regardless heritability

**FIGURE 4:**
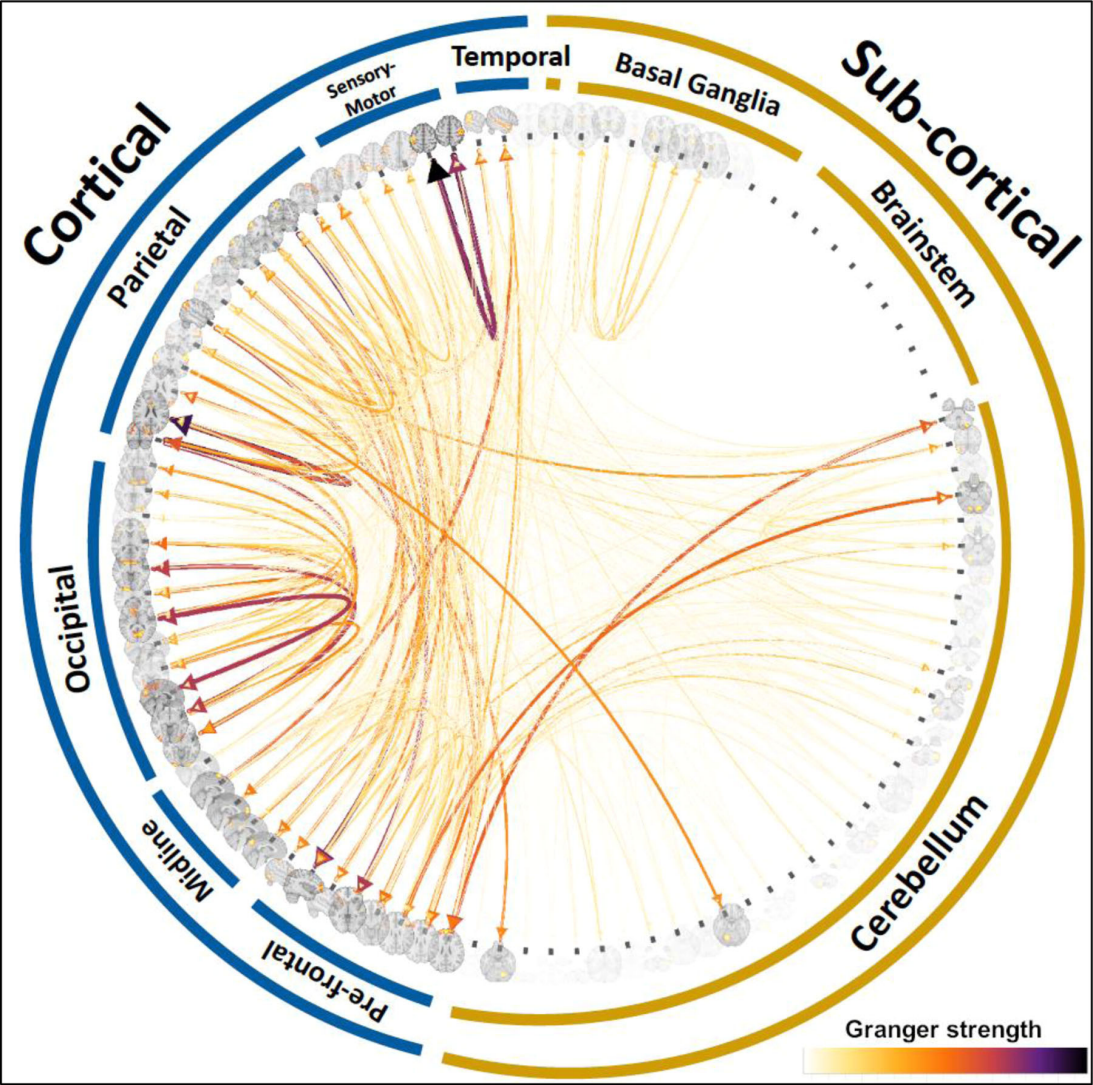
Upper quartile (across connections) of median CG strength across subjects

Figure 4 shows the upper quartile (across connections) of median (across subjects) GC measures. The highest GC strengths were observed in cortical‐to‐cortical connections: most notably within subnetworks of the sensory‐motor network, parietal circuits, and occipital cortices. The direction of the “information flow” evaluated through GC showed bi‐directional cortico‐cortical influences. However, a few asymmetric influences were observed, for example, from parietal to cerebellar areas, from parietal to occipital cortex, and from parietal to prefrontal networks (Bajaj et al., 2016; Chén et al., 2019; Duggento et al., 2018; Lund et al., 2020; Schwab et al., 2018; Xu et al., 2020). Upper quartiles of median GC strengths are provided in Table S5.

#### 3.2.3 Heritability of the directed functional connectome

**FIGURE 5:**
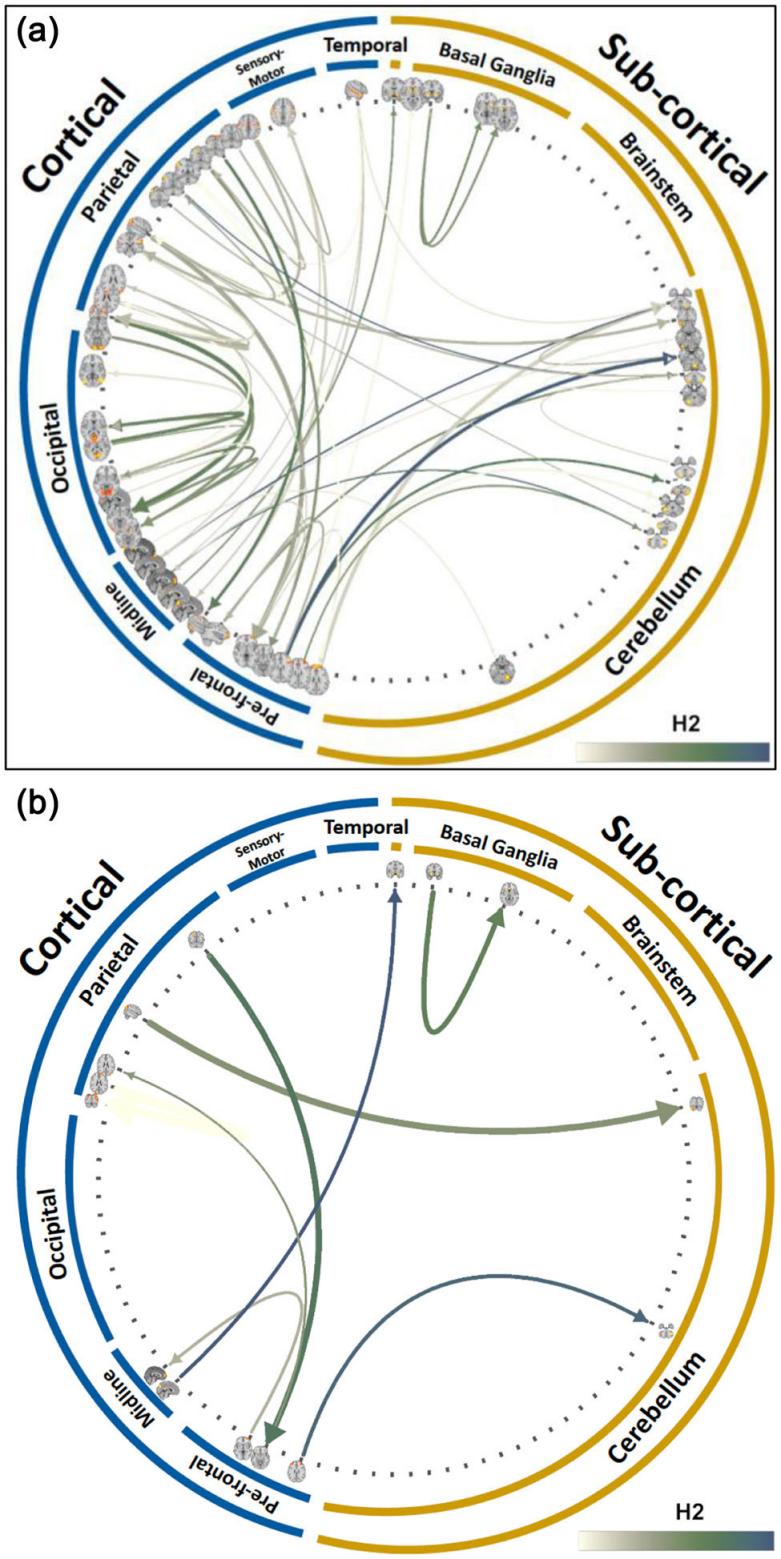
Heritable directed connections in the functional connectome that replicated between the discovery and replication dataset when (a) not modeling the shared environment, and (b) modeling the shared environment. The width of each connection is proportional to the GC strength, while the color scale indicates the heritability value (*h* ^2^)

The heritability scores (*h* ^2^) in the discovery sample ranged from .28 to .55 (mean *h* ^2^ = .36) with *n* = 152 directed connections showing significant effects (*p*’s < .05, FDR corrected). In the replication set, *h* ^2^ values spanned from .27 to .57 (mean *h* ^2^ = .35) with *n* = 241 network connections showing significant heritability (*p*’s < .05, FDR corrected). Fifty‐eight connections with a mean *h* ^2^ ranging from .30 to .50 were significant both in the discovery and replication cohorts (Figure 5a; Table S2).

When the shared environment was modeled, the directed connections showing significant heritability effects were *n* = 56 in the discovery set (.33 to .57, mean *h* ^2^ = .38, CI = .015, *p*’s < .05, FDR corrected) and *n* = 79 in the replication set (.31 to .51, mean *h* ^2^ = .38, CI = .009, *p*’s < .05, FDR corrected) (Table S3). Eight connections were significant both in the discovery and replication cohorts with a mean heritability ranging from .34 to .40, with a mean *h* ^2^ = .37, CI = .015, *p*’s < .05, FDR corrected.(Figure 5b; Table S4). For completeness, we have also carried out an exploratory analysis using the entire data‐set with heritability scores ranging from .18 to .49 (mean *h* ^2^ = .27) with *n* = 313 directed connections showing significant effects (*p*’s < .05, FDR corrected) (Table S6).

## 4 DISCUSSION

We discuss three sets of findings: (1) the strength of the GC connections irrespective of their heritability (Figure 4); (2) the effect of heritability on the directed connections (Figure 5a); (3) the impact of the shared environment on the heritability of directed connectome (Figure 5b). Importantly, each set of findings was replicated between the discovery and replication samples, which were derived from randomly splitting the HCP dataset while carefully matching for a series of potentially confounding variables (age, sex, education, handedness, and kinship).

The fMRI timeseries used to calculate the ICA decomposition were denoised by the HCP consortium using the ICA‐FIX algorithm (Salimi‐Khorshidi et al., 2014). This algorithm carefully examines the data in ICA space via a specific classifier that has been manually trained by experts to remove common sources of fMRI noise including movement, physiological pulsation, and many other artifacts.

We employed State Space GC, a measure that is particularly robust against filtering, downsampling, noise corruption during observation, or any subprocess of a higher dimensional process. Crucially, numerical simulations have demonstrated that SS GC has greater statistical power and smaller bias than the more widely used autoregressive AR estimators. Relative to other methods, SS GC also allows the relaxation of the assumptions of linearity, stationarity, and homoscedasticity. These assumptions are common amongst other methods but are likely not applicable to fMRI data, which have a moving average component  (Barnett & Seth, 2015). We also mitigated the well‐known shortcomings of GC methods applied to fMRI datasets via a deconvolution strategy that limited the lag‐inducing effects of HRF and its spatial variations.

The first group of results regards the strength of the GC connections irrespective of their heritability. The strongest directed connections were found within posterior cortical networks while a weaker strength was found across posterior‐anterior cortical networks and cortico‐subcortical networks. More specifically, high GC strength was observed within the sensory‐motor, parietal, and occipital networks, while intermediate‐to low GC strengths were present across posterior‐anterior cortical networks or within sub‐cortical networks. The strongest connections within the sensory‐motor, parietal, and occipital networks were reciprocated. For example, a sensory‐motor network sending a strong direct connection toward a nearby sensory‐motor component was also the recipient of a strong “backward” connection from such sensory‐motor component. Such “tight” and strong functional interplay between visuo‐parieto and sensory‐motor components is not surprising and is in line with the well‐known anatomical connectivity in these circuits. The feed‐forward and backward patterns of GC connectivity that we found across these networks is also in keeping with the visuo‐spatial processing functions and sensory‐motor transformation that these circuits support.

We now discuss together the second and third set of findings (heritability with or without modeling the shared environment) as these results are conceptually linked. It is also worth noting that the GC strength and GC heritability measures are not dependent on each other in any way. This means that it is possible to have low‐strength GC connections which are highly heritable and/or vice versa.

While no a priori hypothesis on directionality and its heritability was formulated, several previous fMRI studies showed that most heritable connections were in the DMN (Ge et al., 2017; Glahn et al., 2010; Sinclair et al., 2015a), which is in keeping with our results.

In Glahn et al., the heritability estimates involving the DMN (i.e., posterior cingulate/precuneus, medial prefrontal, and cerebellum) ranged from .27 to .42. Colclough et al. (2017) showed an average heritability of 15−18%, while Sinclair et al. showed that graph theoretical metrics derived from resting fMRI activity are under strong genetic influence (.47–.50 heritability). Yang et al., 2016 found that intrinsic functional network properties are heritable (.23−.65) in five of seven networks (i.e., DAN‐dorsal attention network, VN‐visual network, PCN‐posterior default network, DMN‐default mode network, FPN fronto‐parietal network) while 11 of the 21 internetwork coherences were influenced by common environmental factors (.18−.47), similar to our results.

In this study, the heritability scores in the GC connections remained comparable when the shared environment was factored in, ranging from .28–.55 when the shared environment was not modeled, to .34–.40 when shared environment was included in the analyses. However, the number of direct connections showing high heritability and replicability was significantly reduced when the effect of shared environment was modeled (from 58 to 8 connections). This almost depended on the fact that the heritability of the connections within posterior cortical networks (parieto‐parietal, occipito‐occipital, sensory‐motor) did not remain significant when the shared environment was modelled. This suggests a strong influence of shared environmental factors on the cortico‐cortical directed connectivity patterns that involve visuo‐parietal and sensory‐motor circuits, the same networks showing a high GC connectivity strength. Although the heritability of GC connections and their strength are completely different measures, it is interesting that similar posterior cortical connections are not only the strongest but also the most influenced by shared environmental factors. We speculate that this might depend on an intrinsic plasticity of such networks which are constantly engaged by upcoming sensory stimuli.

In contrast, the heritability of the directed connections from parietal to cerebellar components, from parieto to prefrontal cortex (PFC) networks, or from midline to PFC circuits were less influenced by shared environmental factors and showed the highest and most replicable heritability effects. The PFC networks comprised the anterior PFC and dorsolateral/ventrolateral PFC, while the midline cortical structures included the medial PFC (the most anterior part of the DMN), and posterior cingulate cortex/cuneus (a posterior part of the DMN). The main parietal components showing directed functional connections to the PFC and cerebellum were localized in the inferior and superior parietal lobule. Such PFC and DMN components have been implicated in a wide range of high‐order cognitive functions including self‐referential thoughts, theory of mind, and autobiographical memory (Andrews‐Hanna et al., 2014; Buckner et al., 2008).

The cerebellar areas that receive highly hereditable “top‐down” inputs from these cortical regions are the Crus I/II and, to a lesser extent, Crus VIIB. Interestingly, a previous resting‐state fMRI study showed that the Crus I and II are strongly connected with the same PFC and parietal regions identified here, although nor the directionality neither the heritability of these effects were determined (Buckner et al., 2011).

A strong and directional effect of heritability was also found in the connectivity from the posterior cingulate/cuneus component to the medial temporal network including the hippocampus. Similarly, moderate heritability was observed within the basal ganglia (BG), from the ventral to dorsal BG circuits. These cortico‐subcortical circuits support episodic memory (cingulate‐hippocampal network) or habit formation and reward‐driven action control (ventro‐dorsal BG) (Natu et al., 2019). Here, we show that the directed connections between these critical circuits subserving memory or reward‐driven behavior are asymmetrically influenced by hereditary factors.

Together, our findings suggest a robust role of genetic factors in controlling the direction of the “communication” flow within PFC, parietal, hippocampal, BG, and cerebellar networks that mediate several aspects of cognition, learning, and goal‐directed behaviour. On the other hand, our results emphasize that shared environmental factors can have a strong influence on the directed connectivity patterns within posterior occipital and parietal networks which are involved in visuo‐spatial processing and the transformation of sensory input into motor outputs.

It is also worth reflecting on the asymmetric influence of heritability over the above mentioned GC connections. Most of this asymmetry regarded connections from “top‐down” cortical regions such as the PFC and the parietal components to subcortical circuits including the cerebellum and hippocampus. Within the BG, heritability asymmetrically influenced the interaction from the ventral to dorsal striatum. The reason for such asymmetry and how this relates to the neuropsychological functions mediated by these circuits remains an interesting open question for future research. For example, studies using task‐based fMRI coupled with paradigms designed to explore the specific functions of parieto‐PFC, cortico‐cerebellar, BG or parieto‐temporal networks (e.g., visuo‐spatial memory, working memory, learning) are warranted. In principle, these studies can use a similar analytical framework to what employed here to estimate the directionality of the effects within cortico‐cortical or cortico‐subcortical networks, their heritability, and the relationships to behavioral performance.

### 4.1 Strengths and limitations

Our study has strengths and limitations. The split of the sample in a discovery and replication cohort in conjunction with the use of state of art analytical pipelines (i.e., state‐space GC analyses with deconvolution methods) is the first strength of this study. The fact that we employed a highly controlled and publicly available dataset specifically acquired for connectivity studies is a second strength. A third strength is represented by the explicit modeling of the shared environmental factors, which helped disentangling the “pure” effects of heritability.

In terms of shortcomings of the GC analyses applied to rs‐fMRI time‐series, we discuss two main issues, alongside the approaches used to mitigate them. First, the low sampling rate of fMRI data can introduce aliasing effects (Seth et al., 2013). This aspect is partially alleviated by the much shorter repetition time (TR = .72 s) of the HCP protocol, relative to the more conventional fMRI protocols (TR: 1.5–3 s). This particularly short TR guarantees a positive predictive value of almost 100%, especially for the strongest connections (Duggento et al., 2018). Furthermore, the modern state‐space formulation of GC used guarantees better robustness (relative to classic autoregressive approaches) to the down‐sampled, noisy, and filtered rs‐fMRI data (Barnett & Seth, 2015).

Second, the locally varying HRF can introduce non‐uniform delays when estimating the neuronal activity from the BOLD signal (Handwerker et al., 2012). To control for this aspect, we employed a “blind” deconvolution technique, which infers both the shape of the HRF and its underlying neural activity (Wu et al., 2013). This technique has been specifically optimized for rs‐fMRI datasets, where the calculation of the HRF is more difficult than in the presence of a task or stimuli (Wu et al., 2013). The estimated neural activity, deconvolved by the HRF filter, was employed for all connectivity‐related processing.

Other methods, most notably dynamic causal modeling (DCM), are available to assess causality in fMRI data (Stephan et al., 2010). However, DCM was not readily applicable for exploring the directionality of the effects in such large number of networks as those reported here. The intrinsic exploratory nature of our study therefore called for the use of GC methods, although DCM remains a powerful tool to estimate the directionality of the effects in more targeted and hypothesis‐driven networks.

It should also be noted that fMRI connectivity metrics exhibit a high degree of intersubject variability (Conti et al., 2019), which may have limited the accuracy of our findings.

In terms of potential confounds related to participants' behaviour during fMRI scanning, we acknowledge that the HCP consortium did not monitor with eye tracking people's ability to maintain fixation throughout rs‐fMRI scanning. However, eye movements are not known to affect the low‐frequency rs‐fMRI fluctuations, although they can bias magneto‐encephalo‐graphic measures (Muthukumaraswamy, 2013; Orekhova et al., 2015), which we did not study here. It could also be argued that participants’ level of motion during rs‐fMRI scanning is heritable (Couvy‐Duchesne et al., 2014). Movement artifacts are always a concern in fMRI studies, but the HCP pipeline and pre‐processing algorithms are highly robust and reliable in controlling for this caveat (i.e., the HCP‐specific ICA‐FIX automated algorithm has ∼99% sensitivity and specificity in de‐noising the HCP data) (Griffanti et al., 2014; Salimi‐Khorshidi et al., 2014; Smith et al., 2013). Nevertheless, it might be possible that future machine‐learning strategies further improve de‐noising via using deep neural networks with surrogate models (Brescia et al., 2021) or physically informed statistical models (Brescia et al., 2020; Loncarski et al., 2020).

Finally, the statistical model to assess heritability is not without limitations. As per classic twin studies, the heritability estimate (*h* ^2^) only takes into account the additive genetic variance, assuming minimal genetic‐environmental interactions and relatively similar environmental influences in MZ and DZ twins (Boomsma et al., 2002; Mayhew & Meyre, 2017). This is a debatable issue, as two studies supported the equal environment assumption in MZ and DZ twins (Conley et al., 2013; Felson, 2014) while an earlier one did not (Joseph, 1998). Twin designs are also limited in their capacity to reveal the intimate genetic mechanisms underlying heritability. A notable recent work has explored the more nuanced associations between the genetic co‐expression profile and rs‐fMRI functional connectivity patterns, although the directionality of the effects was not ascertained (Bertolero et al., 2019). Future research is warranted to explore how the directed functional connectome maps onto distinct patterns of genetic co‐expression.

## 5 CONCLUSION

Using a discovery and replication sample, alongside with a twin design, we found high heritability in the functional connections from prefrontal and parietal regions to cerebellar areas known to mediate cognitive control and learning. We also observed a strong asymmetric effect of heritability in the connection from the posterior cingulate to the hippocampal components and from ventral to dorsal BG. Together with past literature highlighting the high heritability of the human connectome, our data provide new mechanistic insights to understand the role of genetic factors in controlling the functional connectome.

## CONFLICT OF INTEREST

The authors declare no conflict of interest.

### PEER REVIEW

The peer review history for this article is available at https://publons.com/publon/10.1002/brb3.2839.

## Supporting information

Supplementary Materials

Supplementary Table 5. Upper quartile of median GC across all subjects.

## Data Availability

The data supporting the findings of this study are available in Human Connectome Project (HCP) at (http://www.humanconnectome.org/). These data were derived from the following resources available in the public domain and further information about preprocessing of rs‐fMRI data is available at [https://www.humanconnectome.org/storage/app/media/documentation/s1200/HCP_S1200_Release76_Reference_Manual.pdf]. To estimate state‐space GC, we employed a publicly available Matlab tool with some in‐house modifications in the scripts to include, for each subject, all timeseries in the same model (http://www.lucafaes.net/msGC.html) (Faes et al., 2017).

## References

[brb32839-bib-0001] Abbasi, N. , Duncan, J. , & Rajimehr, R. (2020). Genetic influence is linked to cortical morphology in category‐selective areas of visual cortex. Nature Communications, 11, 709. 10.1038/s41467-020-14610-8 PMC7002610 32024844

[brb32839-bib-0002] Adhikari, B. M. , Jahanshad, N. , Shukla, D. , Glahn, D. C. , Blangero, J. , Reynolds, R. C. , Cox, R. W. , Fieremans, E. , Veraart, J. , Novikov, D. S. , Nichols, T. E. , Hong, L. E. , Thompson, P. M. , & Kochunov, P. (2018). Heritability estimates on resting state fMRI data using ENIGMA analysis pipeline. Pacific Symposium on Biocomputing, 23, 307–318. 29218892 PMC5728672

[brb32839-bib-0003] Almasy, L. , & Blangero, J. (1998). Multipoint quantitative‐trait linkage analysis in general pedigrees. American Journal of Human Genetics, 62, 1198–1211. 10.1086/301844 9545414 PMC1377101

[brb32839-bib-0004] Amos, C. I. (1994). Robust variance‐components approach for assessing genetic linkage in pedigrees. American Journal of Human Genetics, 54, 535–543. 8116623 PMC1918121

[brb32839-bib-0005] Andrews‐Hanna, J. R. , Smallwood, J. , & Spreng, R. N. (2014). The default network and self‐generated thought: Component processes, dynamic control, and clinical relevance. Annals of the New York Academy of Sciences, 1316, 29–52. 10.1111/nyas.12360 24502540 PMC4039623

[brb32839-bib-0006] Bajaj, S. , Adhikari, B. M. , Friston, K. J. , & Dhamala, M. (2016). Bridging the gap: Dynamic causal modeling and granger causality analysis of resting state functional magnetic resonance imaging. Brain Connectivity, 6, 652–661. 10.1089/brain.2016.0422 27506256

[brb32839-bib-0007] Barnett, L. , & Seth, A. K. (2015). Granger causality for state‐space models. Physical Review E, 91, 040101. 10.1103/PhysRevE.91.040101. 25974424

[brb32839-bib-0008] Bertolero, M. A. , Blevins, A. S. , Baum, G. L. , Gur, R. C. , Gur, R. E. , Roalf, D. R. , Satterthwaite, T. D. , & Bassett, D. S. (2019). The human brain's network architecture is genetically encoded by modular pleiotropy. arXiv:1905.07606 [q‐bio].

[brb32839-bib-0009] Blangero, J. , Williams, J. T. , & Almasy, L. (2001). Variance component methods for detecting complex trait loci. Advances in Genetics, 42, 151–181. 10.1016/s0065-2660(01)42021-9 11037320

[brb32839-bib-0010] Boomsma, D. , Busjahn, A. , & Peltonen, L. (2002). Classical twin studies and beyond. Nature Reviews Genetics, 3, 872–882. 10.1038/nrg932 12415317

[brb32839-bib-0066] Brescia, E. , Costantino, D. , Massenio, P. R. , Monopoli, V. G. , Cupertino, F. , & Cascella, G. L. (2021). A design method for the cogging torque minimization of permanent magnet machines with a segmented stator core based on ANN surrogate models. Energies, 14(7), 1880. 10.3390/en14071880.

[brb32839-bib-0067] Brescia, E. , Palmieri, M. , Cascella, G. L. , & Cupertino, F. (2020). Optimal tooth tips design for cogging torque suppression of permanent magnet machines with a segmented stator core. 2020 International Conference on Electrical Machines (ICEM). 10.1109/icem49940.2020.9270968.

[brb32839-bib-0011] Buckner, R. L. , Andrews‐Hanna, J. R. , & Schacter, D. L. (2008). The brain's default network. Annals of the New York Academy of Sciences, 1124, 1–38. 10.1196/annals.1440.011 18400922

[brb32839-bib-0012] Buckner, R. L. , Krienen, F. M. , Castellanos, A. , Diaz, J. C. , & Yeo, B. T. T. (2011). The organization of the human cerebellum estimated by intrinsic functional connectivity. Journal of Neurophysiology, 106, 2322–2345. 10.1152/jn.00339.2011 21795627 PMC3214121

[brb32839-bib-0013] Chén, O. Y. , Cao, H. , Reinen, J. M. , Qian, T. , Gou, J. , Phan, H. , De Vos, M. , & Cannon, T. D. (2019). Resting‐state brain information flow predicts cognitive flexibility in humans. Scientific Reports, 9, 3879. 10.1038/s41598-019-40345-8 30846746 PMC6406001

[brb32839-bib-0014] Colclough, G. L. , Smith, S. M. , Nichols, T. E. , Winkler, A. M. , Sotiropoulos, S. N. , Glasser, M. F. , Van Essen, D. C. , & Woolrich, M. W. (2017). The heritability of multi‐modal connectivity in human brain activity. eLife, 6, e20178. 10.7554/eLife.20178. 28745584 PMC5621837

[brb32839-bib-0015] Conley, D. , Rauscher, E. , Dawes, C. , Magnusson, P. K. E. , & Siegal, M. L. (2013). Heritability and the equal environments assumption: Evidence from multiple samples of misclassified twins. Behavior Genetics, 43, 415–426. 10.1007/s10519-013-9602-1 23903437

[brb32839-bib-0016] Conti, A. , Duggento, A. , Guerrisi, M. , Passamonti, L. , Indovina, I. , & & Toschi, N. (2019). Variability and reproducibility of directed and undirected functional MRI connectomes in the human brain. Entropy, 21(7), 661. 10.3390/e21070661 33267375 PMC7515158

[brb32839-bib-0017] Couvy‐Duchesne, B. , Blokland, G. A. M. , Hickie, I. B. , Thompson, P. M. , Martin, N. G. , de Zubicaray, G. I. , McMahon, K. L. , & Wright, M. J. (2014). Heritability of head motion during resting state functional MRI in 462 healthy twins. Neuroimage, 102, 424–434. 10.1016/j.neuroimage.2014.08.010 25132021 PMC4252775

[brb32839-bib-0018] Duggento, A. , Passamonti, L. , Valenza, G. , Barbieri, R. , Guerrisi, M. , & Toschi, N. (2018). Multivariate Granger causality unveils directed parietal to prefrontal cortex connectivity during task‐free MRI. Scientific Reports, 8, 5571. 10.1038/s41598-018-23996-x 29615790 PMC5882904

[brb32839-bib-0019] Elliott, L. T. , Sharp, K. , Alfaro‐Almagro, F. , Shi, S. , Miller, K. L. , Douaud, G. , Marchini, J. , & Smith, S. M. (2018). Genome‐wide association studies of brain imaging phenotypes in UK biobank. Nature, 562, 210–216. 10.1038/s41586-018-0571-7 30305740 PMC6786974

[brb32839-bib-0020] Elliott, M. L. , Knodt, A. R. , Cooke, M. , Kim, M. J. , Melzer, T. R. , Keenan, R. , Ireland, D. , Ramrakha, S. , Poulton, R. , Caspi, A. , Moffitt, T. E. , & Hariri, A. R. (2019). General functional connectivity: Shared features of resting‐state and task fMRI drive reliable and heritable individual differences in functional brain networks. Neuroimage, 189, 516–532. 10.1016/j.neuroimage.2019.01.068 30708106 PMC6462481

[brb32839-bib-0021] Faes, L. , Nollo, G. , Stramaglia, S. , & Marinazzo, D. (2017). Multiscale Granger causality. Physical Review E, 96, 042150. 10.1103/PhysRevE.96.042150 29347576

[brb32839-bib-0022] Fairchild, G. , Toschi, N. , Sully, K. , Sonuga‐Barke, E. J. S. , Hagan, C. C. , Diciotti, S. , Goodyer, I. M. , Calder, A. J. , & Passamonti, L. (2016). Mapping the structural organization of the brain in conduct disorder: Replication of findings in two independent samples. Journal of Child Psychology and Psychiatry, 57, 1018–1026. 10.1111/jcpp.12581 27306512 PMC4995723

[brb32839-bib-0023] Felson, J. (2014). What can we learn from twin studies? A comprehensive evaluation of the equal environments assumption. Social Science Research, 43, 184–199. 10.1016/j.ssresearch.2013.10.004 24267761

[brb32839-bib-0024] Ge, T. , Holmes, A. J. , Buckner, R. L. , Smoller, J. W. , & Sabuncu, M. R. (2017). Heritability analysis with repeat measurements and its application to resting‐state functional connectivity. Proceedings of the National Academy of Sciences of the United States of America, 114, 5521–5526. 10.1073/pnas.1700765114 28484032 PMC5448225

[brb32839-bib-0025] Ge, T. , Nichols, T. E. , Lee, P. H. , Holmes, A. J. , Roffman, J. L. , Buckner, R. L. , Sabuncu, M. R. , & Smoller, J. W. (2015). Massively expedited genome‐wide heritability analysis (MEGHA). PNAS, 112, 2479–2484. 10.1073/pnas.1415603112 25675487 PMC4345618

[brb32839-bib-0026] Ge, T. , Reuter, M. , Winkler, A. M. , Holmes, A. J. , Lee, P. H. , Tirrell, L. S. , Roffman, J. L. , Buckner, R. L. , Smoller, J. W. , & Sabuncu, M. R. (2016). Multidimensional heritability analysis of neuroanatomical shape. Nature Communications, 7, 13291. 10.1038/ncomms13291 PMC5116071 27845344

[brb32839-bib-0027] Glahn, D. C. , Winkler, A. M. , Kochunov, P. , Almasy, L. , Duggirala, R. , Carless, M. A. , Curran, J. C. , Olvera, R. L. , Laird, A. R. , Smith, S. M. , Beckmann, C. F. , Fox, P. T. , & Blangero, J. (2010). Genetic control over the resting brain. PNAS, 107, 1223–1228. 10.1073/pnas.0909969107 20133824 PMC2824276

[brb32839-bib-0028] Golan, D. , Lander, E. S. , & Rosset, S. (2014). Measuring missing heritability: Inferring the contribution of common variants. Proceedings National Academy of Science USA, 111, E5272. 10.1073/pnas.1419064111 PMC4267399 25422463

[brb32839-bib-0029] Griffanti, L. , Salimi‐Khorshidi, G. , Beckmann, C. F. , Auerbach, E. J. , Douaud, G. , Sexton, C. E. , Zsoldos, E. , Ebmeier, K. P. , Filippini, N. , Mackay, C. E. , Moeller, S. , Xu, J. , Yacoub, E. , Baselli, G. , Ugurbil, K. , Miller, K. L. , & Smith, S. M. (2014). ICA‐based artefact removal and accelerated fMRI acquisition for improved resting state network imaging. Neuroimage, 95, 232–247. 10.1016/j.neuroimage.2014.03.034 24657355 PMC4154346

[brb32839-bib-0030] Handwerker, D. A. , Gonzalez‐Castillo, J. , D'Esposito, M. , & Bandettini, P. A. (2012). The continuing challenge of understanding and modeling hemodynamic variation in fMRI. Neuroimage, 62, 1017–1023. 10.1016/j.neuroimage.2012.02.015 22366081 PMC4180210

[brb32839-bib-0031] Jenkinson, M. , Beckmann, C. F. , Behrens, T. E. J. , Woolrich, M. W. , & Smith, S. M. (2012). Fsl. Neuroimage, *62*, 782–790. 10.1016/j.neuroimage.2011.09.015 21979382

[brb32839-bib-0032] Joseph, J. (1998). The equal environment assumption of the classical twin method: A critical analysis. The Journal of Mind and Behavior, 19, 325–358.

[brb32839-bib-0033] Kelly, R. M. , & Strick, P. L. (2003). Cerebellar loops with motor cortex and prefrontal cortex of a nonhuman primate. Journal of Neuroscience, 23, 8432–8444. 12968006 10.1523/JNEUROSCI.23-23-08432.2003 PMC6740694

[brb32839-bib-0034] Koziol, L. F. , Budding, D. , Andreasen, N. , D'Arrigo, S. , Bulgheroni, S. , Imamizu, H. , Ito, M. , Manto, M. , Marvel, C. , Parker, K. , Pezzulo, G. , Ramnani, N. , Riva, D. , Schmahmann, J. , Vandervert, L. , & Yamazaki, T. (2014). Consensus paper: The cerebellum's role in movement and cognition. Cerebellum (London, England), 13, 151–177. 10.1007/s12311-013-0511-x 23996631 PMC4089997

[brb32839-bib-0068] Loncarski, J. , Monopoli, V. G. , Cascella, G. L. , & Cupertino, F. (2020). SiC‐MOSFET and Si‐IGBT‐Based dc‐dc Interleaved Converters for EV Chargers: Approach for Efficiency Comparison with Minimum Switching Losses Based on Complete Parasitic Modeling. Energies, 13(17), 4585. 10.3390/en13174585.

[brb32839-bib-0035] Lund, M. J. , Alnæs, D. , Schwab, S. , Meer, D. v. d. , Andreassen, O. A. , Westlye, L. T. , & Kaufmann, T. (2020). Differences in directed functional brain connectivity related to age, sex and mental health. Human Brain Mapping, 41, 4173–4186. 10.1002/hbm.25116 32613721 PMC7502836

[brb32839-bib-0036] Marcus, D. S. , Harms, M. P. , Snyder, A. Z. , Jenkinson, M. , Wilson, J. A. , Glasser, M. F. , Barch, D. M. , Archie, K. A. , Burgess, G. C. , Ramaratnam, M. , Hodge, M. , Horton, W. , Herrick, R. , Olsen, T. , McKay, M. , House, M. , Hileman, M. , Reid, E. , Harwell, J. , … Consortium W. U.‐M. H. C. P . (2013). Human connectome project informatics: Quality control, database services, and data visualization. Neuroimage, 80, 202–219. 10.1016/j.neuroimage.2013.05.077 23707591 PMC3845379

[brb32839-bib-0037] Mayhew, A. J. , & Meyre, D. (2017). Assessing the heritability of complex traits in humans: Methodological challenges and opportunities. Current Genomics, 18, 332–340. 10.2174/1389202918666170307161450 29081689 PMC5635617

[brb32839-bib-0038] Miranda‐Dominguez, O. , Feczko, E. , Grayson, D. S. , Walum, H. , Nigg, J. T. , & Fair, D. A. (2017a). Heritability of the human connectome: A connectotyping study. Network Neuroscience, 2, 175–199. 10.1162/netn_a_00029 PMC6130446 30215032

[brb32839-bib-0039] Miranda‐Dominguez, O. , Feczko, E. , Grayson, D. S. , Walum, H. , Nigg, J. T. , & Fair, D. A. (2017b). Heritability of the human connectome: A connectotyping study. Network Neuroscience, 2, 175–199. 10.1162/netn_a_00029 PMC6130446 30215032

[brb32839-bib-0040] Muthukumaraswamy, S. (2013). High‐frequency brain activity and muscle artifacts in MEG/EEG: A review and recommendations. Frontiers in Human Neuroscience, 7, 10.3389/fnhum.2013.00138 PMC3625857 23596409

[brb32839-bib-0041] Natu, V. S. , Lin, J. J. , Burks, A. , Arora, A. , Rugg, M. D. , & & Lega, B. (2019). Sep 4. Stimulation of the posterior cingulate cortex impairs episodic memory encoding. Journal of Neuroscience, 39(36), 7173–7182. 10.1523/JNEUROSCI.0698-19.2019 31358651 PMC6733540

[brb32839-bib-0042] Orekhova, E. V. , Butorina, A. V. , Sysoeva, O. V. , Prokofyev, A. O. , Nikolaeva, A. Y. U. , & Stroganova, T. A. (2015). Frequency of gamma oscillations in humans is modulated by velocity of visual motion. Journal of Neurophysiology, 114, 244–255. 10.1152/jn.00232.2015 25925324 PMC4507959

[brb32839-bib-0043] Polderman, J. C. , Benyamin, B. , Leeuw, C. A. d. , Sullivan, P. F. , Bochoven, A. , Visscher, P. M. , & Posthuma, D. (2015). Meta‐analysis of the heritability of human traits based on fifty years of twin studies. Nat Gen, 47, 702–709. 10.1038/ng.3285 25985137

[brb32839-bib-0044] Rangaprakash, D. , Wu, G. ‐R. , Marinazzo, D. , Hu, X. , & Deshpande, G. (2018). Hemodynamic response function (HRF) variability confounds resting‐state fMRI functional connectivity. Magnetic Resonance in Medicine, 80, 1697–1713. 10.1002/mrm.27146 29656446

[brb32839-bib-0045] Reineberg, A. E. , Hatoum, A. S. , Hewitt, J. K. , Banich, M. T. , & Friedman, N. P. (2020). Genetic and environmental influence on the human functional connectome. Cerebral Cortex, 30, 2099–2113. 10.1093/cercor/bhz225 31711120 PMC7175002

[brb32839-bib-0046] Salimi‐Khorshidi, G. , Douaud, G. , Beckmann, C. F. , Glasser, M. F. , Griffanti, L. , & Smith, S. M. (2014). Automatic denoising of functional MRI data: Combining independent component analysis and hierarchical fusion of classifiers. Neuroimage, 90, 449–468. 10.1016/j.neuroimage.2013.11.046 24389422 PMC4019210

[brb32839-bib-0047] Schmahmann, J. D. , & Pandya, D. N. (1997). Anatomic organization of the basilar pontine projections from prefrontal cortices in rhesus monkey. Journal of Neuroscience, 17, 438–458. 10.1523/JNEUROSCI.17-01-00438.1997 8987769 PMC6793685

[brb32839-bib-0048] Schwab, S. , Harbord, R. , Zerbi, V. , Elliott, L. , Afyouni, S. , Smith, J. Q. , Woolrich, M. W. , Smith, S. M. , & Nichols, T. E. (2018). Directed functional connectivity using dynamic graphical models. Neuroimage, 175, 340–353. 10.1016/j.neuroimage.2018.03.074 29625233 PMC6153304

[brb32839-bib-0049] Seth, A. K. , Barrett, A. B. , & Barnett, L. (2015). Granger causality analysis in neuroscience and neuroimaging. Journal of Neuroscience, 35, 3293–3297. 10.1523/JNEUROSCI.4399-14.2015 25716830 PMC4339347

[brb32839-bib-0050] Seth, A. K. , Chorley, P. , & Barnett, L. C. (2013). Granger causality analysis of fMRI BOLD signals is invariant to hemodynamic convolution but not downsampling. Neuroimage, 65, 540–555. 10.1016/j.neuroimage.2012.09.049 23036449

[brb32839-bib-0051] Shine, J. M. , Bissett, P. G. , Bell, P. T. , Koyejo, O. , Balsters, J. H. , Gorgolewski, K. J. , Moodie, C. A. , & Poldrack, R. A. (2016). The dynamics of functional brain networks: Integrated network states during cognitive task performance. Neuron, 92, 544–554. 10.1016/j.neuron.2016.09.018 27693256 PMC5073034

[brb32839-bib-0052] Sinclair, B. , Hansell, N. K. , Blokland, G. A. M. , Martin, N. G. , Thompson, P. M. , Breakspear, M. , de Zubicaray, G. I. , Wright, M. J. , & McMahon, K. L. (2015a). Heritability of the network architecture of intrinsic brain functional connectivity. Neuroimage, 121, 243–252. 10.1016/j.neuroimage.2015.07.048 26226088 PMC4837693

[brb32839-bib-0053] Sinclair, B. , Hansell, N. K. , Blokland, G. A. M. , Martin, N. G. , Thompson, P. M. , Breakspear, M. , de Zubicaray, G. I. , Wright, M. J. , & McMahon, K. L. (2015b). Heritability of the network architecture of intrinsic brain functional connectivity. Neuroimage, 121, 243–252. 10.1016/j.neuroimage.2015.07.048 26226088 PMC4837693

[brb32839-bib-0054] Smith, S. M. , Beckmann, C. F. , Andersson, J. , Auerbach, E. J. , Bijsterbosch, J. , Douaud, G. , Duff, E. , Feinberg, D. A. , Griffanti, L. , Harms, M. P. , Kelly, M. , Laumann, T. , Miller, K. L. , Moeller, S. , Petersen, S. , Power, J. , Salimi‐Khorshidi, G. , Snyder, A. Z. , Vu, A. T. , … Consortium W. U.‐M. H. C. P . (2013). Resting‐state fMRI in the human connectome project. Neuroimage, 80, 144–168. 10.1016/j.neuroimage.2013.05.039 23702415 PMC3720828

[brb32839-bib-0055] Stephan, K. E. , Penny, W. D. , Moran, R. J. , den Ouden, H. E. M. , Daunizeau, J. , & Friston, K. J. (2010). Ten simple rules for dynamic causal modeling. Neuroimage, 49, 3099–3109. 10.1016/j.neuroimage.2009.11.015 19914382 PMC2825373

[brb32839-bib-0056] Toschi, N. , Duggento, A. , & Passamonti, L. (2017). Functional connectivity in amygdalar‐sensory/(pre)motor networks at rest: New evidence from the human connectome project. European Journal of Neuroscience, 45, 1224–1229. 10.1111/ejn.13544 28231395

[brb32839-bib-0057] Van Essen, D. C. , Smith, S. M. , Barch, D. M. , Behrens, T. E. J. , Yacoub, E. , Ugurbil, K. , & Consortium W. U.‐M. H. C. P . (2013). The WU‐Minn human connectome project: An overview. Neuroimage, 80, 62–79. 10.1016/j.neuroimage.2013.05.041 23684880 PMC3724347

[brb32839-bib-0058] Wedel, I. (1962). Falconer, d. S.: Introduction to quantitative genetics. Oliver and boyd, edinburgh and london 1960; 365 S., 35 s. Biometrische Zeitschrift, 4, 140–141. 10.1002/bimj.19620040211

[brb32839-bib-0059] Wu, G.‐R. , Di Perri, C. , Charland‐Verville, V. , Martial, C. , Carrière, M. , Vanhaudenhuyse, A. , Laureys, S. , & Marinazzo, D. (2019). Modulation of the spontaneous hemodynamic response function across levels of consciousness. Neuroimage, 200, 450–459. 10.1016/j.neuroimage.2019.07.011 31284028

[brb32839-bib-0060] Wu, G.‐R. , Liao, W. , Stramaglia, S. , Ding, J.‐R. , Chen, H. , & Marinazzo, D. (2013). A blind deconvolution approach to recover effective connectivity brain networks from resting state fMRI data. Medical Image Analysis, 17, 365–374. 10.1016/j.media.2013.01.003 23422254

[brb32839-bib-0061] Xu, N. , Doerschuk, P. C. , Keilholz, S. D. , & Spreng, R. N. (2020). Spatiotemporal functional interactivity among large‐scale brain networks. bioRxiv 2020.04.14.041830. 10.1101/2020.04.14.041830 33316394

[brb32839-bib-0062] Yang, J. , Benyamin, B. , McEvoy, B. P. , Gordon, S. , Henders, A. K. , Nyholt, D. R. , Madden, P. A. , Heath, A. C. , Martin, N. G. , Montgomery, G. W. , Goddard, M. E. , & Visscher, P. M. (2010). Common SNPs explain a large proportion of the heritability for human height. Nature Genetics, 42, 565–569. 10.1038/ng.608 20562875 PMC3232052

[brb32839-bib-0063] Yang, J. , Lee, S. H. , Goddard, M. E. , & Visscher, P. M. (2011). GCTA: A tool for genome‐wide complex trait analysis. American Journal of Human Genetics, 88, 76–82. 10.1016/j.ajhg.2010.11.011 21167468 PMC3014363

[brb32839-bib-0064] Yang, Z. , Zuo, X.‐N. , McMahon, K. L. , Craddock, R. C. , Kelly, C. , de Zubicaray, G. I. , Hickie, I. , Bandettini, P. A. , Castellanos, F. X. , Milham, M. P. , & Wright, M. J. (2016a). Genetic and environmental contributions to functional connectivity architecture of the human brain. Cerebral Cortex, 26, 2341–2352. 10.1093/cercor/bhw02. 26891986 PMC4830303

