# Supplementary material for: Heritability of human “directed” functional connectome
Source: Brain Behav. 2023 Mar 29;13(5):e2839. doi: 10.1002/brb3.2839 (PMC10175995; doi:10.1002/brb3.2839)
Supplement: Supplementary file 1 — Supplementary Materials [file BRB3-13-e2839-s002.docx]

**Supplementary Materials**

*Heritability of the human ‘directed’ functional connectome*

*Maria Giovanna Bianco, Andrea Duggento, Salvatore Nigro, Allegra Conti, Nicola Toschi, Luca Passamonti*

***Supplementary Table 1*.** Demographic variables in the discovery and replication set.

|  | **MZ** | | | | **DZ** | | | | **Siblings** | | | | **Singletons** | | | |
| --- | --- | --- | --- | --- | --- | --- | --- | --- | --- | --- | --- | --- | --- | --- | --- | --- |
|  | **Discovery set** | **Replication set** | **t/χ2** | **p-value** | **Discovery set** | **Replication set** | **t/χ2** | **p-value** | **Discovery set** | **Replication set** | **t/χ2** | **p-value** | **Discovery set** | **Replication set** | **t/χ2** | **p-value** |
| Gender (males/females) | 52/72 | 52/72 | 0 | 1.00 | 22/28 | 22/28 | 0 | 1.00 | 103/94 | 103/94 | 0 | 1.00 | 37/41 | 37/41 | 0 | 1.00 |
| Age  (years) | 29.0 ± 3.3 | 29.3 ± 3.4 | -0.55 | 0.58 | 29.5±3.5 | 29.5 ± 3.5 | 0.03 | 0.98 | 28.4±3.8 | 28.1 ± 3.8 | 0.75 | 0.46 | 29.1±4.1 | 29.0 ± 3.8 | 0.08 | 0.94 |
| Handedness (right/left/both) | 118/6/0 | 110/14/0 | 3.48 | 0.10 | 45/5/0 | 48/2/0 | 1.382 | 0.24 | 183/14/0 | 178/16/3 | 2.82 | 0.24 | 67/11/0 | 71/7/0 | 1.01 | 0.45 |
| Education  (years) | 15.1 ± 1.8 | 14.8 ± 1.9 | 1.16 | 0.24 | 15.2 ± 1.7 | 15.1 ± 1.7 | -0.49 | 0.62 | 15.0 ± 1.7 | 15.0 ± 1.7 | -0.36 | 0.72 | 14.7 ± 1.8 | 14.8 ± 1.8 | -0.12 | 0.90 |

***Supplementary Table 2*.** Heritability of GC connections that matches between the discovery and replication set (FDR: false discovery rate correction for multiple comparisons).

***Supplementary* *Table 3.*** Heritability of GC connections in discovery and replication set (FDR: false discovery rate correction for multiple comparisons) taking in account the common shared environment.

.

***Table 4***. Heritability of GC connections that matches between the discovery and replication set (FDR: false discovery rate correction for multiple comparisons) with the common shared environment

|  | | |
| --- | --- | --- |
| **Connections** | | **Discovery/Replication** |
| **ICA node #1** | **ICA node #2** | **mean h^2^** |
| ICA13  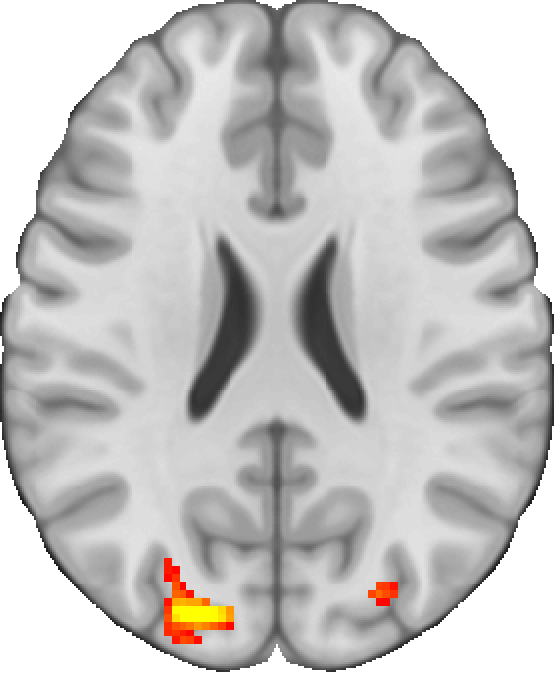 | ICA8  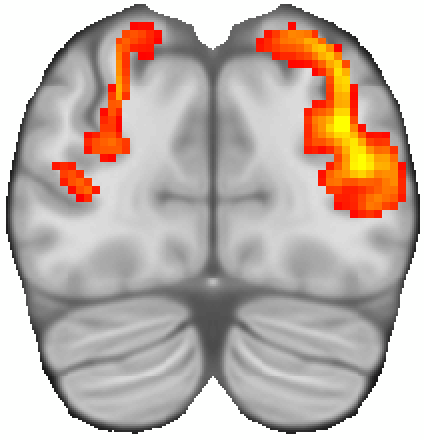 | 0.3374151 |
| ICA14  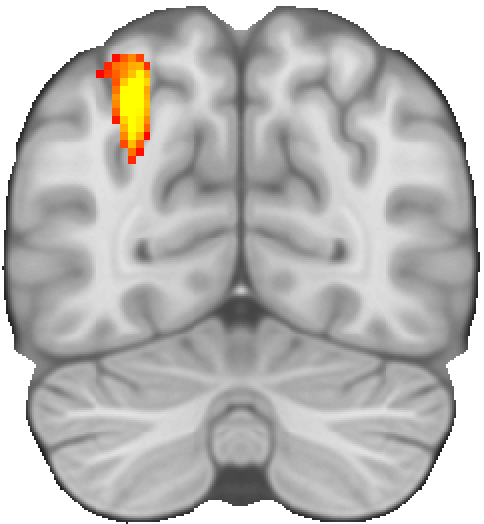 | ICA44  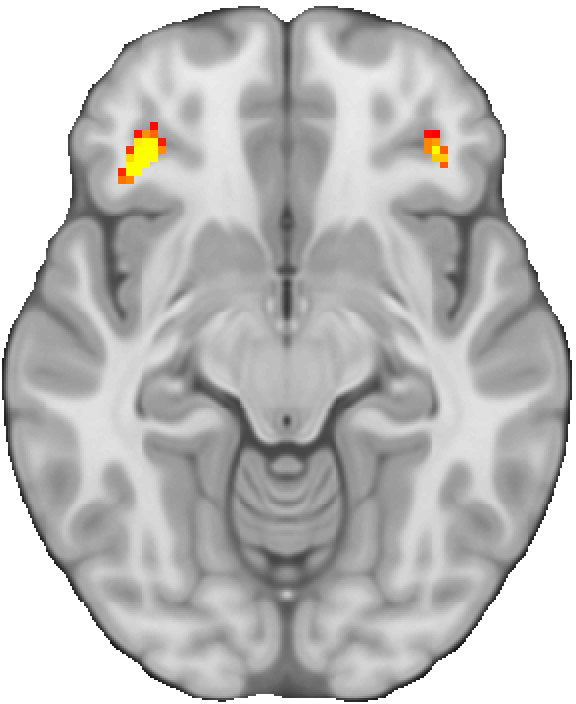 | 0.38707965 |
| ICA15  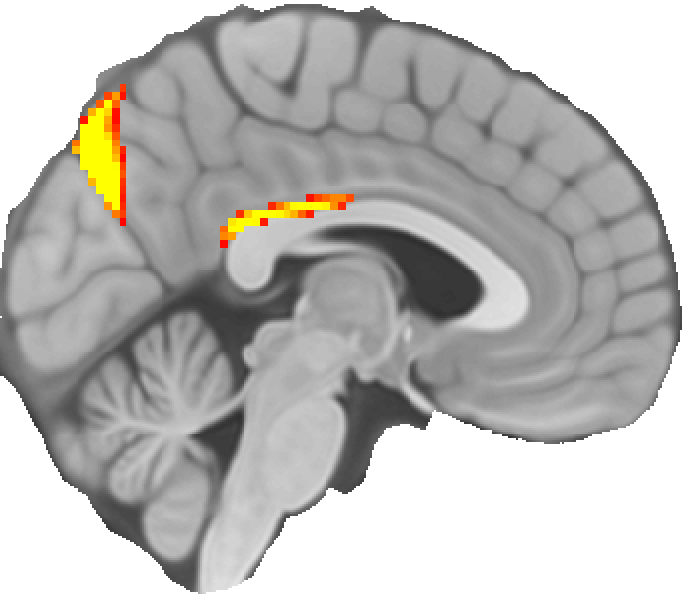 | ICA60  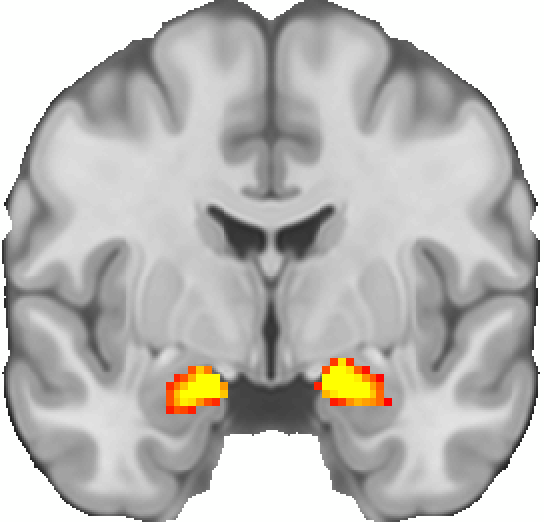 | 0.4035691 |
| ICA26  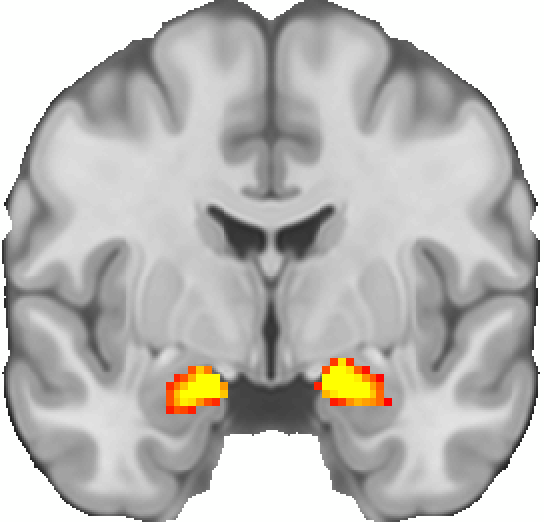 | ICA61  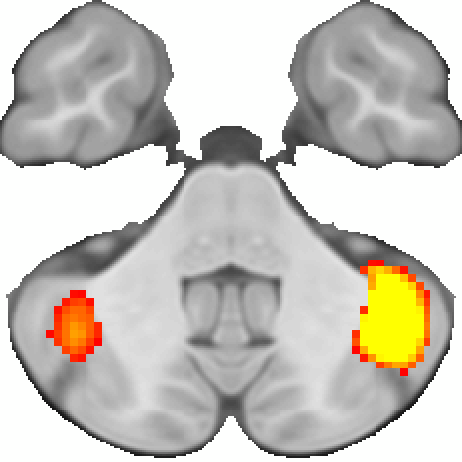 | 0.3972464 |
| ICA2  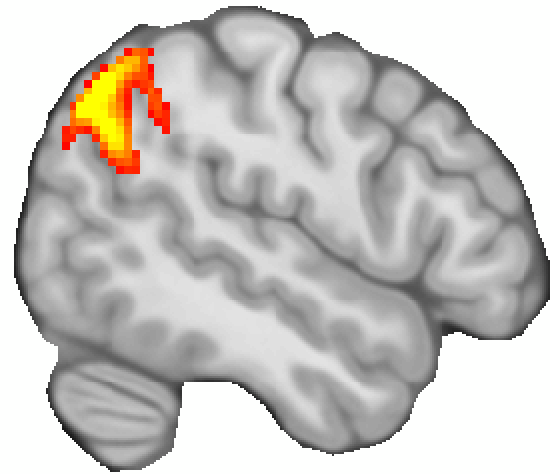 | ICA46  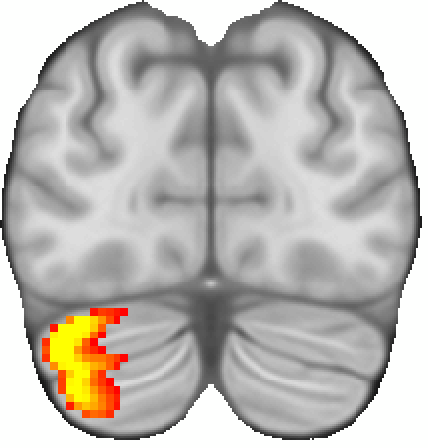 | 0.36801735 |
| ICA44  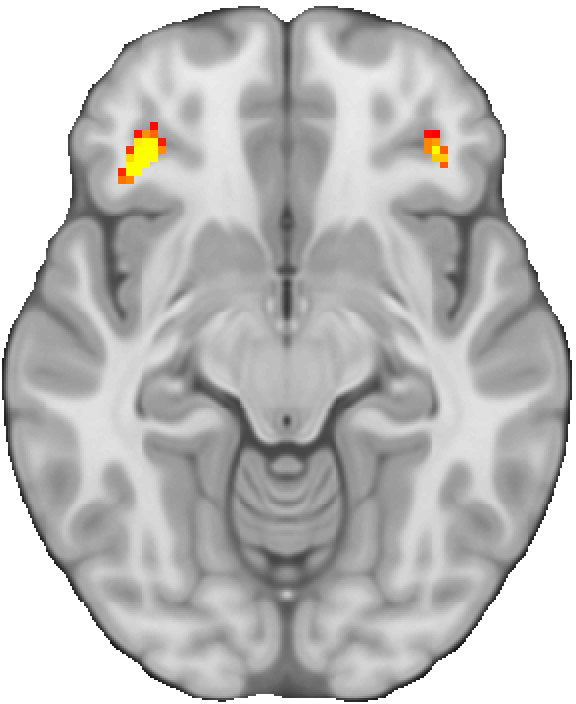 | ICA20  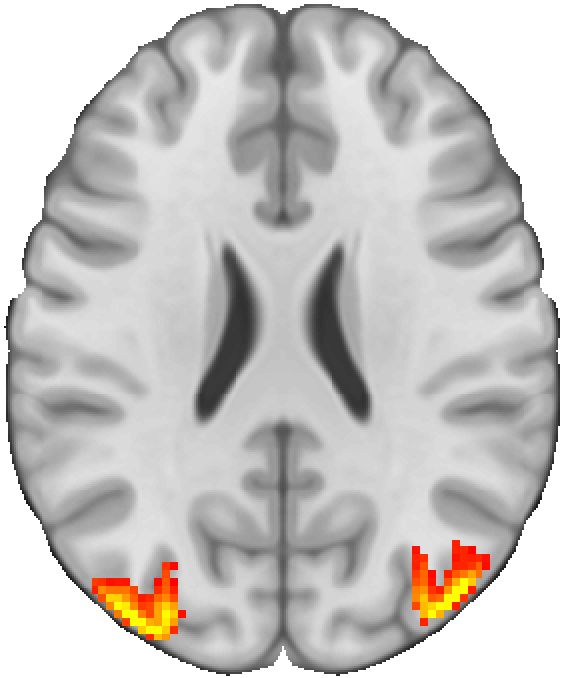 | 0.3666998 |
| ICA48  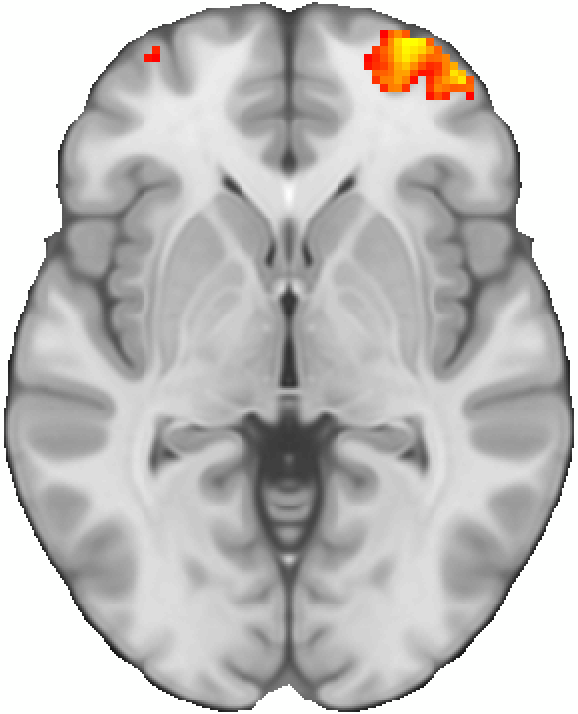 | ICA36  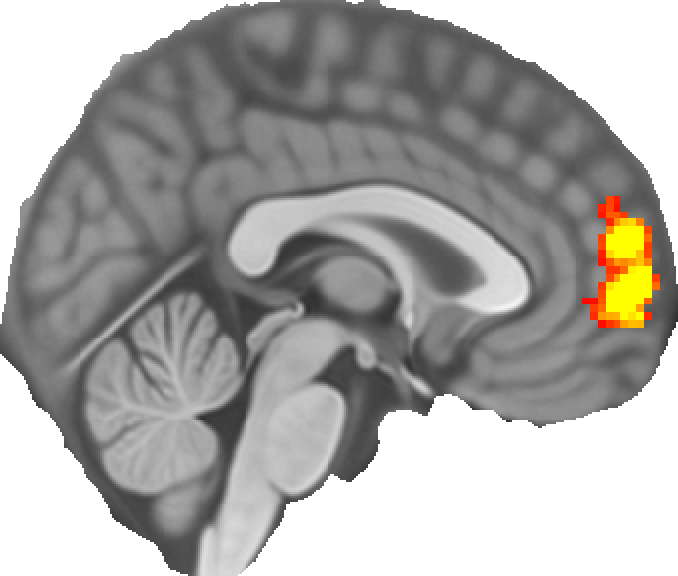 | 0.3565373 |
| ICA65  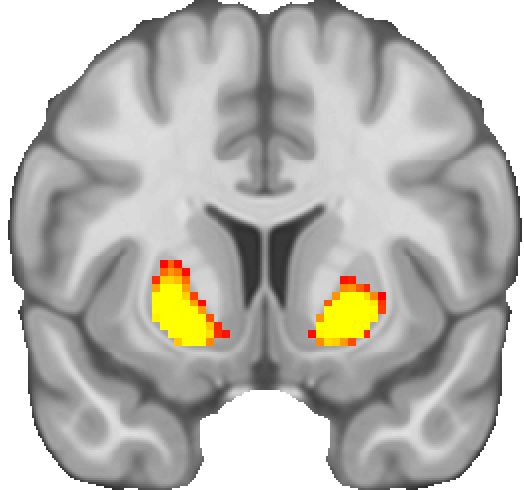 | ICA79  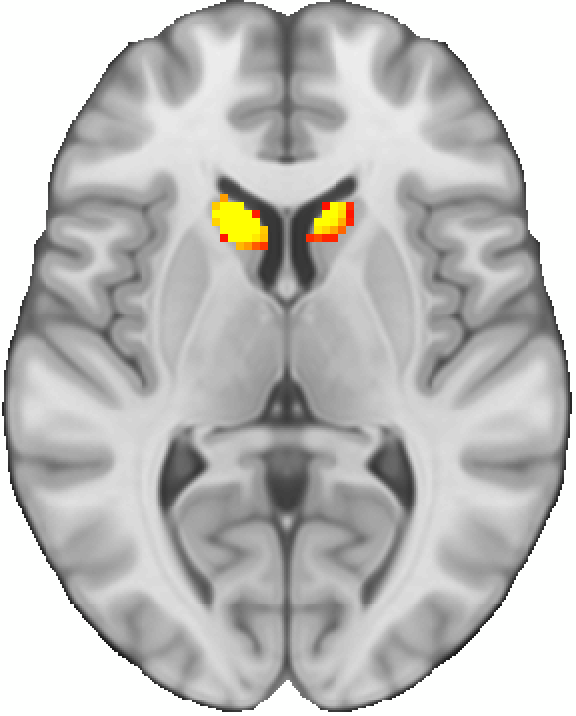 | 0.3796197 |

***Supplementary* *Table 6***. Heritability of GC connections in the overall Dataset (FDR: false discovery rate correction for multiple comparisons) with the common shared environment.

| **Connections** | **ALL DATASET** |  | **Connections** | **ALL DATASET** |  | **Connections** | **ALL DATASET** |
| --- | --- | --- | --- | --- | --- | --- | --- |
| **ICA node #1 - ICA node #2** | **mean h^2^** |  | **ICA node #1 - ICA node #2** | **mean h^2^** |  | **ICA node #1 - ICA node #2** | **mean h^2^** |
| ICA9-ICA51 | 0,491 |  | ICA17-ICA28 | 0,340 |  | ICA2-ICA12 | 0,332 |
| ICA36-ICA61 | 0,452 |  | ICA48-ICA22 | 0,336 |  | ICA40-ICA9 | 0,326 |
| ICA11-ICA1 | 0,425 |  | ICA33-ICA48 | 0,354 |  | ICA22-ICA26 | 0,305 |
| ICA26-ICA61 | 0,409 |  | ICA17-ICA30 | 0,328 |  | ICA7-ICA52 | 0,325 |
| ICA9-ICA2 | 0,435 |  | ICA1-ICA3 | 0,321 |  | ICA21-ICA77 | 0,327 |
| ICA17-ICA19 | 0,402 |  | ICA23-ICA35 | 0,329 |  | ICA24-ICA34 | 0,306 |
| ICA40-ICA50 | 0,461 |  | ICA29-ICA26 | 0,334 |  | ICA48-ICA36 | 0,332 |
| ICA13-ICA8 | 0,384 |  | ICA31-ICA66 | 0,342 |  | ICA17-ICA11 | 0,336 |
| ICA39-ICA1 | 0,410 |  | ICA37-ICA29 | 0,335 |  | ICA21-ICA92 | 0,311 |
| ICA6-ICA51 | 0,374 |  | ICA37-ICA55 | 0,321 |  | ICA22-ICA51 | 0,312 |
| ICA3-ICA1 | 0,417 |  | ICA38-ICA35 | 0,348 |  | ICA14-ICA51 | 0,306 |
| ICA11-ICA19 | 0,398 |  | ICA40-ICA33 | 0,335 |  | ICA29-ICA36 | 0,315 |
| ICA40-ICA57 | 0,419 |  | ICA40-ICA61 | 0,354 |  | ICA2-ICA22 | 0,308 |
| ICA14-ICA44 | 0,362 |  | ICA41-ICA78 | 0,331 |  | ICA41-ICA8 | 0,302 |
| ICA65-ICA76 | 0,379 |  | ICA42-ICA51 | 0,338 |  | ICA26-ICA58 | 0,296 |
| ICA28-ICA11 | 0,383 |  | ICA65-ICA79 | 0,360 |  | ICA5-ICA24 | 0,310 |
| ICA44-ICA20 | 0,362 |  | ICA6-ICA10 | 0,352 |  | ICA12-ICA31 | 0,320 |
| ICA17-ICA4 | 0,355 |  | ICA9-ICA20 | 0,307 |  | ICA14-ICA33 | 0,294 |
| ICA15-ICA60 | 0,377 |  | ICA12-ICA29 | 0,315 |  | ICA17-ICA8 | 0,307 |
| ICA24-ICA14 | 0,369 |  | ICA26-ICA51 | 0,328 |  | ICA7-ICA29 | 0,288 |
| ICA45-ICA22 | 0,367 |  | ICA37-ICA49 | 0,323 |  | ICA11-ICA20 | 0,317 |
| ICA31-ICA56 | 0,376 |  | ICA44-ICA9 | 0,348 |  | ICA16-ICA11 | 0,317 |
| ICA26-ICA59 | 0,354 |  | ICA4-ICA11 | 0,326 |  | ICA7-ICA66 | 0,312 |
| ICA24-ICA7 | 0,352 |  | ICA52-ICA50 | 0,320 |  | ICA20-ICA8 | 0,336 |
| ICA13-ICA4 | 0,367 |  | ICA56-ICA46 | 0,346 |  | ICA8-ICA13 | 0,287 |
| ICA12-ICA42 | 0,355 |  | ICA26-ICA68 | 0,309 |  | ICA19-ICA3 | 0,285 |
| ICA35-ICA37 | 0,352 |  | ICA15-ICA48 | 0,321 |  | ICA3-ICA8 | 0,306 |
| ICA2-ICA57 | 0,354 |  | ICA24-ICA61 | 0,328 |  | ICA23-ICA34 | 0,301 |

| **Connections** | **ALL DATASET** |  | **Connections** | **ALL DATASET** |  | **Connections** | **ALL DATASET** |
| --- | --- | --- | --- | --- | --- | --- | --- |
| **ICA node #1 - ICA node #2** | **mean h^2^** |  | **ICA node #1 - ICA node #2** | **mean h^2^** |  | **ICA node #1 - ICA node #2** | **mean h^2^** |
| ICA12-ICA22 | 0,315 |  | ICA37-ICA64 | 0,294 |  | ICA11-ICA17 | 0,245 |
| ICA19-ICA67 | 0,297 |  | ICA24-ICA5 | 0,297 |  | ICA19-ICA4 | 0,261 |
| ICA2-ICA20 | 0,301 |  | ICA38-ICA58 | 0,274 |  | ICA18-ICA48 | 0,243 |
| ICA44-ICA51 | 0,280 |  | ICA12-ICA33 | 0,285 |  | ICA10-ICA51 | 0,271 |
| ICA40-ICA59 | 0,306 |  | ICA24-ICA42 | 0,290 |  | ICA7-ICA2 | 0,267 |
| ICA8-ICA11 | 0,280 |  | ICA10-ICA56 | 0,284 |  | ICA1-ICA7 | 0,273 |
| ICA29-ICA10 | 0,307 |  | ICA22-ICA48 | 0,262 |  | ICA5-ICA52 | 0,253 |
| ICA50-ICA51 | 0,286 |  | ICA40-ICA48 | 0,299 |  | ICA9-ICA7 | 0,241 |
| ICA16-ICA28 | 0,294 |  | ICA37-ICA22 | 0,259 |  | ICA25-ICA1 | 0,285 |
| ICA10-ICA7 | 0,285 |  | ICA21-ICA38 | 0,298 |  | ICA40-ICA52 | 0,257 |
| ICA31-ICA70 | 0,291 |  | ICA23-ICA18 | 0,294 |  | ICA4-ICA17 | 0,266 |
| ICA40-ICA10 | 0,281 |  | ICA31-ICA14 | 0,281 |  | ICA40-ICA14 | 0,271 |
| ICA33-ICA58 | 0,308 |  | ICA15-ICA45 | 0,293 |  | ICA1-ICA28 | 0,253 |
| ICA44-ICA14 | 0,293 |  | ICA35-ICA56 | 0,281 |  | ICA6-ICA8 | 0,277 |
| ICA33-ICA55 | 0,282 |  | ICA12-ICA24 | 0,241 |  | ICA33-ICA50 | 0,268 |
| ICA13-ICA16 | 0,304 |  | ICA37-ICA40 | 0,290 |  | ICA5-ICA83 | 0,244 |
| ICA27-ICA4 | 0,291 |  | ICA37-ICA56 | 0,267 |  | ICA36-ICA31 | 0,263 |
| ICA89-ICA84 | 0,304 |  | ICA9-ICA10 | 0,267 |  | ICA37-ICA57 | 0,264 |
| ICA26-ICA44 | 0,297 |  | ICA18-ICA34 | 0,278 |  | ICA42-ICA53 | 0,250 |
| ICA34-ICA23 | 0,290 |  | ICA33-ICA44 | 0,259 |  | ICA52-ICA49 | 0,249 |
| ICA47-ICA78 | 0,293 |  | ICA1-ICA39 | 0,253 |  | ICA6-ICA19 | 0,235 |
| ICA44-ICA56 | 0,279 |  | ICA20-ICA18 | 0,269 |  | ICA6-ICA64 | 0,252 |
| ICA31-ICA57 | 0,274 |  | ICA22-ICA36 | 0,252 |  | ICA26-ICA33 | 0,257 |
| ICA38-ICA69 | 0,287 |  | ICA33-ICA40 | 0,276 |  | ICA13-ICA66 | 0,257 |
| ICA12-ICA51 | 0,287 |  | ICA33-ICA14 | 0,255 |  | ICA8-ICA6 | 0,241 |
| ICA40-ICA44 | 0,280 |  | ICA17-ICA34 | 0,263 |  | ICA28-ICA1 | 0,256 |
| ICA40-ICA30 | 0,297 |  | ICA37-ICA33 | 0,288 |  | ICA10-ICA83 | 0,252 |
| ICA22-ICA42 | 0,295 |  | ICA56-ICA42 | 0,273 |  | ICA6-ICA30 | 0,277 |

| **Connections** | **ALL DATASET** |  | **Connections** | **ALL DATASET** |  | **Connections** | **ALL DATASET** |
| --- | --- | --- | --- | --- | --- | --- | --- |
| **ICA node #1 - ICA node #2** | **mean h^2^** |  | **ICA node #1 - ICA node #2** | **mean h^2^** |  | **ICA node #1 - ICA node #2** | **mean h^2^** |
| ICA12-ICA22 | 0,315 |  | ICA37-ICA64 | 0,294 |  | ICA11-ICA17 | 0,245 |
| ICA19-ICA67 | 0,297 |  | ICA24-ICA5 | 0,297 |  | ICA19-ICA4 | 0,261 |
| ICA2-ICA20 | 0,301 |  | ICA38-ICA58 | 0,274 |  | ICA18-ICA48 | 0,243 |
| ICA44-ICA51 | 0,280 |  | ICA12-ICA33 | 0,285 |  | ICA10-ICA51 | 0,271 |
| ICA40-ICA59 | 0,306 |  | ICA24-ICA42 | 0,290 |  | ICA7-ICA2 | 0,267 |
| ICA8-ICA11 | 0,280 |  | ICA10-ICA56 | 0,284 |  | ICA1-ICA7 | 0,273 |
| ICA29-ICA10 | 0,307 |  | ICA22-ICA48 | 0,262 |  | ICA5-ICA52 | 0,253 |
| ICA50-ICA51 | 0,286 |  | ICA40-ICA48 | 0,299 |  | ICA9-ICA7 | 0,241 |
| ICA16-ICA28 | 0,294 |  | ICA37-ICA22 | 0,259 |  | ICA25-ICA1 | 0,285 |
| ICA10-ICA7 | 0,285 |  | ICA21-ICA38 | 0,298 |  | ICA40-ICA52 | 0,257 |
| ICA31-ICA70 | 0,291 |  | ICA23-ICA18 | 0,294 |  | ICA4-ICA17 | 0,266 |
| ICA40-ICA10 | 0,281 |  | ICA31-ICA14 | 0,281 |  | ICA40-ICA14 | 0,271 |
| ICA33-ICA58 | 0,308 |  | ICA15-ICA45 | 0,293 |  | ICA1-ICA28 | 0,253 |
| ICA44-ICA14 | 0,293 |  | ICA35-ICA56 | 0,281 |  | ICA6-ICA8 | 0,277 |
| ICA33-ICA55 | 0,282 |  | ICA12-ICA24 | 0,241 |  | ICA33-ICA50 | 0,268 |
| ICA13-ICA16 | 0,304 |  | ICA37-ICA40 | 0,290 |  | ICA5-ICA83 | 0,244 |
| ICA27-ICA4 | 0,291 |  | ICA37-ICA56 | 0,267 |  | ICA36-ICA31 | 0,263 |
| ICA89-ICA84 | 0,304 |  | ICA9-ICA10 | 0,267 |  | ICA37-ICA57 | 0,264 |
| ICA26-ICA44 | 0,297 |  | ICA18-ICA34 | 0,278 |  | ICA42-ICA53 | 0,250 |
| ICA34-ICA23 | 0,290 |  | ICA33-ICA44 | 0,259 |  | ICA52-ICA49 | 0,249 |
| ICA47-ICA78 | 0,293 |  | ICA1-ICA39 | 0,253 |  | ICA6-ICA19 | 0,235 |
| ICA44-ICA56 | 0,279 |  | ICA20-ICA18 | 0,269 |  | ICA6-ICA64 | 0,252 |
| ICA31-ICA57 | 0,274 |  | ICA22-ICA36 | 0,252 |  | ICA26-ICA33 | 0,257 |
| ICA38-ICA69 | 0,287 |  | ICA33-ICA40 | 0,276 |  | ICA13-ICA66 | 0,257 |
| ICA12-ICA51 | 0,287 |  | ICA33-ICA14 | 0,255 |  | ICA8-ICA6 | 0,241 |
| ICA40-ICA44 | 0,280 |  | ICA17-ICA34 | 0,263 |  | ICA28-ICA1 | 0,256 |
| ICA40-ICA30 | 0,297 |  | ICA37-ICA33 | 0,288 |  | ICA10-ICA83 | 0,252 |
| ICA22-ICA42 | 0,295 |  | ICA56-ICA42 | 0,273 |  | ICA6-ICA30 | 0,277 |

| **Connections** | **ALL DATASET** |  | **Connections** | **ALL DATASET** |  | **Connections** | **ALL DATASET** |
| --- | --- | --- | --- | --- | --- | --- | --- |
| **ICA node #1 - ICA node #2** | **mean h^2^** |  | **ICA node #1 - ICA node #2** | **mean h^2^** |  | **ICA node #1 - ICA node #2** | **mean h^2^** |
| ICA11-ICA28 | 0,205 |  | ICA9-ICA66 | 0,1915 |  | ICA4-ICA39 | 0,183 |
| ICA37-ICA83 | 0,215 |  | ICA12-ICA90 | 0,2040 |  | ICA68-ICA50 | 0,179 |
| ICA14-ICA61 | 0,211 |  | ICA33-ICA56 | 0,1942 |  | ICA20-ICA24 | 0,194 |
| ICA28-ICA25 | 0,200 |  | ICA29-ICA30 | 0,2245 |  | ICA4-ICA23 | 0,194 |
| ICA48-ICA24 | 0,198 |  | ICA24-ICA56 | 0,1876 |  | ICA60-ICA48 | 0,181 |
| ICA10-ICA44 | 0,211 |  | ICA24-ICA51 | 0,1979 |  |  |  |
| ICA6-ICA20 | 0,219 |  | ICA55-ICA42 | 0,2093 |  |  |  |
| ICA12-ICA60 | 0,226 |  | ICA48-ICA45 | 0,1896 |  |  |  |
| ICA37-ICA51 | 0,214 |  | ICA55-ICA11 | 0,1946 |  |  |  |
| ICA22-ICA46 | 0,221 |  | ICA23-ICA15 | 0,2020 |  |  |  |
| ICA17-ICA51 | 0,206 |  | ICA18-ICA72 | 0,2117 |  |  |  |
| ICA38-ICA23 | 0,206 |  | ICA24-ICA12 | 0,1996 |  |  |  |
| ICA38-ICA62 | 0,204 |  | ICA85-ICA66 | 0,1860 |  |  |  |
| ICA37-ICA12 | 0,205 |  | ICA28-ICA17 | 0,2047 |  |  |  |
| ICA39-ICA32 | 0,209 |  | ICA13-ICA19 | 0,1992 |  |  |  |
| ICA31-ICA29 | 0,207 |  | ICA18-ICA42 | 0,2033 |  |  |  |
| ICA10-ICA64 | 0,216 |  | ICA37-ICA88 | 0,1954 |  |  |  |
| ICA77-ICA94 | 0,195 |  | ICA37-ICA75 | 0,1969 |  |  |  |
| ICA40-ICA54 | 0,204 |  | ICA34-ICA61 | 0,1855 |  |  |  |
| ICA31-ICA40 | 0,214 |  | ICA34-ICA57 | 0,1991 |  |  |  |
| ICA38-ICA17 | 0,213 |  | ICA42-ICA54 | 0,1877 |  |  |  |
| ICA3-ICA23 | 0,204 |  | ICA24-ICA44 | 0,2017 |  |  |  |
| ICA38-ICA21 | 0,194 |  | ICA11-ICA16 | 0,1892 |  |  |  |
| ICA85-ICA12 | 0,197 |  | ICA38-ICA84 | 0,1908 |  |  |  |
| ICA58-ICA13 | 0,198 |  | ICA24-ICA40 | 0,1875 |  |  |  |
| ICA68-ICA61 | 0,190 |  | ICA38-ICA55 | 0,1956 |  |  |  |
| ICA27-ICA20 | 0,197 |  | ICA26-ICA21 | 0,1845 |  |  |  |
| ICA8-ICA31 | 0,219 |  | ICA36-ICA9 | 0,1787 |  |  |  |
