# Supplementary material for: Heritability of human “directed” functional connectome
Source: Brain Behav. 2023 Mar 29;13(5):e2839. doi: 10.1002/brb3.2839 (PMC10175995; doi:10.1002/brb3.2839)
Supplement: Supplementary file 2 — Supplementary Table 5. Upper quartile of median GC across all subjects. [file BRB3-13-e2839-s001.pdf]

Supplementary Table 5. Upper quartile of median GC across all subjects.

Connections node #1 →	node #2	GC Strength x10^2
ICA 97	ICA 96	1.711
ICA 80	ICA 81	1.547
ICA 90	ICA 61	1.428
ICA 96	ICA 97	1.304
ICA 70	ICA 73	1.244
ICA 73	ICA 70	1.237
ICA 85	ICA 59	1.232
ICA 80	ICA 69	1.215
ICA 68	ICA 75	1.199
ICA 89	ICA 55	1.144
ICA 88	ICA 61	1.102
ICA 81	ICA 80	1.062
ICA 76	ICA 68	1.016
ICA 55	ICA 21	0.988
ICA 66	ICA 99	0.979
ICA 91	ICA 82	0.960

Connections node #1 →	node #2	GC Strength x10^2
ICA 68	ICA 76	0.950
ICA 57	ICA 24	0.945
ICA 95	ICA 62	0.918
ICA 64	ICA 58	0.904
ICA 56	ICA 55	0.888
ICA 55	ICA 56	0.886
ICA 88	ICA 65	0.876
ICA 87	ICA 80	0.874
ICA 85	ICA 55	0.871
ICA 88	ICA 98	0.869
ICA 60	ICA 92	0.865
ICA 92	ICA 60	0.865
ICA 87	ICA 63	0.860
ICA 73	ICA 74	0.852
ICA 62	ICA 91	0.829
ICA 90	ICA 92	0.814

Connections node #1 →	node #2	GC Strength x10^2
ICA 61	ICA 88	0.806
ICA 83	ICA 53	0.796
ICA 68	ICA 79	0.786
ICA 91	ICA 62	0.778
ICA 75	ICA 68	0.765
ICA 83	ICA 44	0.761
ICA 68	ICA 71	0.758
ICA 81	ICA 70	0.753
ICA 56	ICA 58	0.748
ICA 62	ICA 94	0.725
ICA 57	ICA 92	0.720
ICA 68	ICA 78	0.709
ICA 79	ICA 68	0.703
ICA 56	ICA 57	0.701
ICA 85	ICA 22	0.699
ICA 60	ICA 63	0.690

Connections node #1 →	node #2	GC Strength x10^2
ICA 61	ICA 90	0.679
ICA 93	ICA 84	0.678
ICA 69	ICA 68	0.671
ICA 81	ICA 72	0.669
ICA 63	ICA 61	0.662
ICA 63	ICA 87	0.647
ICA 63	ICA 88	0.645
ICA 80	ICA 87	0.645
ICA 66	ICA 55	0.644
ICA 70	ICA 72	0.634
ICA 88	ICA 58	0.633
ICA 58	ICA 88	0.631
ICA 90	ICA 58	0.629
ICA 56	ICA 99	0.619
ICA 69	ICA 79	0.613
ICA 89	ICA 85	0.610

Connections node #1	→ node #2	GC Strength $x10^2$
ICA 62	ICA 95	0.603
ICA 84	ICA 80	0.595
ICA 60	ICA 26	0.593
ICA 98	ICA 88	0.591
ICA 5	ICA 6	0.581
ICA 86	ICA 88	0.578
ICA 98	ICA 86	0.577
ICA 57	ICA 31	0.570
ICA 88	ICA 63	0.569
ICA 71	ICA 76	0.566
ICA 89	ICA 61	0.564
ICA 92	ICA 86	0.549
ICA 76	ICA 72	0.545
ICA 71	ICA 74	0.532
ICA 57	ICA 59	0.530
ICA 65	ICA 89	0.523

Connections node #1	→ node #2	GC Strength $x10^2$
ICA 69	ICA 80	0.518
ICA 71	ICA 68	0.517
ICA 55	ICA 99	0.513
ICA 91	ICA 94	0.512
ICA 70	ICA 81	0.504
ICA 78	ICA 68	0.502
ICA 3	ICA 7	0.501
ICA 71	ICA 81	0.499
ICA 92	ICA 89	0.498
ICA 98	ICA 62	0.498
ICA 56	ICA 32	0.497
ICA 89	ICA 98	0.495
ICA 66	ICA 61	0.493
ICA 86	ICA 80	0.489
ICA 83	ICA 93	0.480
ICA 91	ICA 85	0.476

Connections node #1	→ node #2	GC Strength $x10^2$
ICA 58	ICA 56	0.474
ICA 67	ICA 95	0.473
ICA 58	ICA 59	0.469
ICA 69	ICA 75	0.469
ICA 81	ICA 71	0.463
ICA 90	ICA 98	0.460
ICA 3	ICA 2	0.457
ICA 93	ICA 73	0.455
ICA 57	ICA 34	0.450
ICA 91	ICA 65	0.446
ICA 85	ICA 91	0.445
ICA 57	ICA 56	0.445
ICA 90	ICA 84	0.444
ICA 58	ICA 64	0.444
ICA 86	ICA 98	0.440
ICA 92	ICA 90	0.439

Connections node #1	→ node #2	GC Strength $x10^2$
ICA 90	ICA 80	0.437
ICA 6	ICA 5	0.436
ICA 86	ICA 97	0.429
ICA 88	ICA 82	0.426
ICA 92	ICA 57	0.425
ICA 71	ICA 72	0.424
ICA 89	ICA 92	0.424
ICA 95	ICA 96	0.423
ICA 56	ICA 92	0.418
ICA 85	ICA 89	0.418
ICA 99	ICA 56	0.417
ICA 60	ICA 99	0.409
ICA 6	ICA 3	0.408
ICA 86	ICA 92	0.404
ICA 73	ICA 77	0.404
ICA 69	ICA 78	0.401

Connections node #1 →	node #2	GC Strength x10^2
ICA 3	ICA 6	0.398
ICA 61	ICA 25	0.397
ICA 85	ICA 26	0.394
ICA 57	ICA 61	0.393
ICA 80	ICA 84	0.390
ICA 55	ICA 60	0.389
ICA 82	ICA 91	0.387
ICA 89	ICA 1	0.386
ICA 72	ICA 70	0.385
ICA 92	ICA 59	0.385
ICA 57	ICA 2	0.382
ICA 59	ICA 85	0.382
ICA 65	ICA 61	0.381
ICA 87	ICA 69	0.380
ICA 79	ICA 69	0.375
ICA 84	ICA 90	0.373

Connections node #1 →	node #2	GC Strength x10^2
ICA 30	ICA 22	0.372
ICA 76	ICA 74	0.371
ICA 93	ICA 81	0.367
ICA 98	ICA 30	0.366
ICA 73	ICA 76	0.365
ICA 62	ICA 61	0.364
ICA 99	ICA 60	0.364
ICA 92	ICA 85	0.363
ICA 65	ICA 91	0.363
ICA 56	ICA 24	0.362
ICA 84	ICA 69	0.361
ICA 55	ICA 65	0.361
ICA 87	ICA 81	0.357
ICA 66	ICA 98	0.355
ICA 60	ICA 85	0.353
ICA 94	ICA 62	0.352

Connections node #1 →	node #2	GC Strength x10^2
ICA 58	ICA 86	0.350
ICA 90	ICA 31	0.350
ICA 60	ICA 82	0.350
ICA 61	ICA 63	0.350
ICA 75	ICA 76	0.349
ICA 90	ICA 99	0.348
ICA 61	ICA 59	0.348
ICA 84	ICA 86	0.347
ICA 71	ICA 73	0.345
ICA 23	ICA 27	0.345
ICA 95	ICA 94	0.343
ICA 56	ICA 34	0.341
ICA 85	ICA 88	0.341
ICA 86	ICA 58	0.340
ICA 89	ICA 21	0.340
ICA 88	ICA 57	0.339

Connections node #1 →	node #2	GC Strength x10^2
ICA 63	ICA 65	0.338
ICA 59	ICA 92	0.336
ICA 94	ICA 91	0.334
ICA 86	ICA 60	0.332
ICA 72	ICA 71	0.332
ICA 95	ICA 40	0.331
ICA 59	ICA 58	0.331
ICA 95	ICA 93	0.330
ICA 87	ICA 82	0.330
ICA 80	ICA 86	0.330
ICA 91	ICA 86	0.330
ICA 76	ICA 71	0.327
ICA 90	ICA 55	0.326
ICA 99	ICA 66	0.325
ICA 64	ICA 1	0.325
ICA 56	ICA 61	0.323

Connections		GC
node #1 →	node #2	Strength x10^2
ICA 96	ICA 67	0.321
ICA 87	ICA 25	0.320
ICA 93	ICA 95	0.320
ICA 85	ICA 90	0.319
ICA 76	ICA 78	0.317
ICA 2	ICA 7	0.316
ICA 88	ICA 56	0.316
ICA 66	ICA 21	0.316
ICA 88	ICA 86	0.316
ICA 93	ICA 55	0.315
ICA 5	ICA 3	0.315
ICA 87	ICA 55	0.313
ICA 24	ICA 25	0.313
ICA 92	ICA 56	0.312
ICA 63	ICA 60	0.311
ICA 99	ICA 61	0.308

Connections		GC
node #1 →	node #2	Strength x10^2
ICA 67	ICA 96	0.308
ICA 84	ICA 81	0.307
ICA 64	ICA 93	0.306
ICA 99	ICA 63	0.306
ICA 86	ICA 66	0.305
ICA 84	ICA 49	0.303
ICA 55	ICA 89	0.303
ICA 78	ICA 75	0.303
ICA 92	ICA 61	0.301
ICA 85	ICA 82	0.300
ICA 81	ICA 73	0.300
ICA 72	ICA 76	0.300
ICA 21	ICA 27	0.300
ICA 93	ICA 59	0.299
ICA 66	ICA 47	0.298
ICA 85	ICA 60	0.294

Connections		GC
node #1 →	node #2	Strength x10^2
ICA 66	ICA 36	0.294
ICA 92	ICA 94	0.294
ICA 86	ICA 28	0.294
ICA 92	ICA 55	0.292
ICA 82	ICA 88	0.290
ICA 99	ICA 90	0.290
ICA 65	ICA 99	0.290
ICA 55	ICA 86	0.290
ICA 97	ICA 86	0.290
ICA 85	ICA 28	0.288
ICA 65	ICA 88	0.287
ICA 56	ICA 38	0.287
ICA 60	ICA 86	0.287
ICA 57	ICA 84	0.286
ICA 89	ICA 56	0.285
ICA 87	ICA 77	0.285

Connections		GC
node #1 →	node #2	Strength x10^2
ICA 61	ICA 24	0.285
ICA 62	ICA 98	0.283
ICA 71	ICA 82	0.283
ICA 64	ICA 89	0.283
ICA 57	ICA 63	0.283
ICA 61	ICA 98	0.282
ICA 80	ICA 82	0.282
ICA 68	ICA 69	0.282
ICA 24	ICA 30	0.282
ICA 56	ICA 88	0.282
ICA 60	ICA 91	0.282
ICA 7	ICA 2	0.280
ICA 90	ICA 62	0.279
ICA 91	ICA 59	0.278
ICA 88	ICA 30	0.277
ICA 99	ICA 55	0.277

Connections		GC
node #1 →	node #2	Strength $x10^2$
ICA 74	ICA 70	0.276
ICA 99	ICA 1	0.276
ICA 57	ICA 21	0.276
ICA 95	ICA 67	0.275
ICA 88	ICA 85	0.274
ICA 90	ICA 57	0.274
ICA 74	ICA 69	0.273
ICA 95	ICA 36	0.273
ICA 32	ICA 33	0.271
ICA 85	ICA 31	0.271
ICA 32	ICA 23	0.270
ICA 76	ICA 75	0.269
ICA 90	ICA 59	0.269
ICA 97	ICA 67	0.268
ICA 95	ICA 97	0.268
ICA 94	ICA 95	0.268

Connections		GC
node #1 →	node #2	Strength $x10^2$
ICA 80	ICA 77	0.267
ICA 85	ICA 84	0.267
ICA 58	ICA 91	0.266
ICA 60	ICA 59	0.266
ICA 86	ICA 63	0.265
ICA 88	ICA 91	0.265
ICA 59	ICA 90	0.264
ICA 58	ICA 25	0.264
ICA 57	ICA 88	0.263
ICA 100	ICA 55	0.263
ICA 56	ICA 25	0.263
ICA 55	ICA 85	0.262
ICA 63	ICA 99	0.262
ICA 82	ICA 80	0.262
ICA 84	ICA 91	0.261
ICA 98	ICA 90	0.261

Connections		GC
node #1 →	node #2	Strength $x10^2$
ICA 59	ICA 66	0.261
ICA 61	ICA 62	0.260
ICA 95	ICA 58	0.260
ICA 98	ICA 89	0.259
ICA 55	ICA 66	0.259
ICA 60	ICA 30	0.258
ICA 25	ICA 21	0.257
ICA 65	ICA 98	0.256
ICA 61	ICA 65	0.256
ICA 99	ICA 93	0.256
ICA 3	ICA 5	0.256
ICA 77	ICA 68	0.255
ICA 85	ICA 66	0.255
ICA 91	ICA 96	0.255
ICA 92	ICA 26	0.255
ICA 89	ICA 59	0.254

Connections		GC
node #1 →	node #2	Strength $x10^2$
ICA 97	ICA 49	0.253
ICA 70	ICA 62	0.251
ICA 91	ICA 21	0.251
ICA 31	ICA 24	0.251
ICA 60	ICA 33	0.251
ICA 77	ICA 70	0.250
ICA 86	ICA 84	0.250
ICA 72	ICA 81	0.250
ICA 73	ICA 81	0.249
ICA 70	ICA 71	0.249
ICA 65	ICA 69	0.249
ICA 82	ICA 61	0.249
ICA 84	ICA 57	0.249
ICA 60	ICA 88	0.249
ICA 81	ICA 69	0.246
ICA 74	ICA 73	0.246

Connections: node #1	→ node #2	GC: Strength $x10^2$
ICA 90	ICA 29	0.244
ICA 86	ICA 34	0.244
ICA 74	ICA 93	0.243
ICA 57	ICA 58	0.243
ICA 85	ICA 81	0.243
ICA 56	ICA 65	0.243
ICA 56	ICA 64	0.242
ICA 74	ICA 4	0.242
ICA 61	ICA 89	0.242
ICA 66	ICA 29	0.241
ICA 99	ICA 86	0.241
ICA 99	ICA 21	0.241
ICA 55	ICA 92	0.241
ICA 86	ICA 99	0.241
ICA 80	ICA 71	0.241
ICA 57	ICA 90	0.240

Connections: node #1	→ node #2	GC: Strength $x10^2$
ICA 59	ICA 65	0.240
ICA 85	ICA 86	0.240
ICA 82	ICA 78	0.239
ICA 65	ICA 66	0.239
ICA 55	ICA 90	0.238
ICA 67	ICA 56	0.238
ICA 83	ICA 94	0.238
ICA 55	ICA 93	0.238
ICA 61	ICA 99	0.238
ICA 93	ICA 89	0.237
ICA 75	ICA 69	0.236
ICA 80	ICA 60	0.236
ICA 89	ICA 24	0.235
ICA 61	ICA 29	0.235
ICA 84	ICA 60	0.235
ICA 66	ICA 85	0.234

Connections: node #1	→ node #2	GC: Strength $x10^2$
ICA 89	ICA 62	0.234
ICA 63	ICA 36	0.234
ICA 85	ICA 92	0.233
ICA 80	ICA 90	0.233
ICA 91	ICA 90	0.233
ICA 94	ICA 92	0.232
ICA 64	ICA 92	0.231
ICA 45	ICA 23	0.231
ICA 95	ICA 50	0.229
ICA 96	ICA 91	0.229
ICA 73	ICA 71	0.229
ICA 97	ICA 46	0.229
ICA 72	ICA 74	0.228
ICA 84	ICA 35	0.228
ICA 98	ICA 56	0.228
ICA 98	ICA 66	0.227

Connections: node #1	→ node #2	GC: Strength $x10^2$
ICA 93	ICA 67	0.226
ICA 90	ICA 60	0.226
ICA 60	ICA 65	0.226
ICA 60	ICA 56	0.225
ICA 66	ICA 59	0.225
ICA 56	ICA 33	0.224
ICA 59	ICA 55	0.224
ICA 60	ICA 90	0.224
ICA 68	ICA 77	0.223
ICA 100	ICA 94	0.223
ICA 61	ICA 82	0.223
ICA 26	ICA 22	0.222
ICA 26	ICA 23	0.222
ICA 92	ICA 82	0.222
ICA 90	ICA 69	0.222
ICA 87	ICA 35	0.221

Connections		GC
node #1 →	node #2	Strength $x10^2$
ICA 65	ICA 59	0.221
ICA 8	ICA 4	0.221
ICA 64	ICA 100	0.221
ICA 75	ICA 78	0.220
ICA 86	ICA 56	0.219
ICA 82	ICA 95	0.219
ICA 87	ICA 58	0.218
ICA 97	ICA 82	0.216
ICA 55	ICA 22	0.216
ICA 95	ICA 89	0.216
ICA 92	ICA 79	0.215
ICA 24	ICA 31	0.214
ICA 29	ICA 23	0.214
ICA 94	ICA 64	0.213
ICA 63	ICA 25	0.213
ICA 71	ICA 69	0.213

Connections		GC
node #1 →	node #2	Strength $x10^2$
ICA 98	ICA 37	0.212
ICA 68	ICA 73	0.212
ICA 93	ICA 26	0.212
ICA 81	ICA 35	0.211
ICA 88	ICA 33	0.211
ICA 58	ICA 85	0.211
ICA 87	ICA 86	0.210
ICA 56	ICA 87	0.210
ICA 91	ICA 30	0.210
ICA 58	ICA 90	0.210
ICA 89	ICA 94	0.209
ICA 94	ICA 73	0.208
ICA 87	ICA 95	0.208
ICA 95	ICA 84	0.208
ICA 87	ICA 70	0.207
ICA 67	ICA 97	0.206

Connections		GC
node #1 →	node #2	Strength $x10^2$
ICA 5	ICA 2	0.206
ICA 58	ICA 60	0.206
ICA 100	ICA 89	0.206
ICA 63	ICA 47	0.206
ICA 96	ICA 45	0.206
ICA 99	ICA 57	0.206
ICA 79	ICA 74	0.206
ICA 92	ICA 62	0.206
ICA 21	ICA 28	0.205
ICA 93	ICA 24	0.205
ICA 64	ICA 62	0.205
ICA 99	ICA 25	0.205
ICA 85	ICA 77	0.205
ICA 90	ICA 85	0.204
ICA 70	ICA 79	0.204
ICA 22	ICA 30	0.204

Connections		GC
node #1 →	node #2	Strength $x10^2$
ICA 28	ICA 27	0.204
ICA 73	ICA 80	0.203
ICA 69	ICA 74	0.203
ICA 98	ICA 21	0.202
ICA 57	ICA 77	0.202
ICA 62	ICA 90	0.202
ICA 55	ICA 57	0.202
ICA 63	ICA 93	0.202
ICA 88	ICA 60	0.201
ICA 64	ICA 52	0.200
ICA 57	ICA 42	0.199
ICA 27	ICA 28	0.199
ICA 2	ICA 85	0.199
ICA 95	ICA 65	0.199
ICA 87	ICA 36	0.199
ICA 55	ICA 87	0.198

Connections		GC
node #1 →	node #2	Strength x10[2]
ICA 61	ICA 57	0.198
ICA 74	ICA 1	0.198
ICA 85	ICA 21	0.197
ICA 66	ICA 30	0.197
ICA 62	ICA 80	0.197
ICA 64	ICA 94	0.197
ICA 93	ICA 83	0.197
ICA 57	ICA 74	0.196
ICA 57	ICA 52	0.196
ICA 65	ICA 84	0.195
ICA 25	ICA 28	0.195
ICA 96	ICA 43	0.195
ICA 42	ICA 47	0.195
ICA 64	ICA 70	0.194
ICA 84	ICA 36	0.194
ICA 80	ICA 63	0.194

Connections		GC
node #1 →	node #2	Strength x10[2]
ICA 47	ICA 42	0.194
ICA 93	ICA 29	0.193
ICA 94	ICA 40	0.193
ICA 56	ICA 69	0.192
ICA 65	ICA 70	0.192
ICA 63	ICA 58	0.191
ICA 86	ICA 96	0.191
ICA 92	ICA 21	0.191
ICA 7	ICA 85	0.190
ICA 65	ICA 90	0.190
ICA 85	ICA 62	0.190
ICA 57	ICA 43	0.190
ICA 75	ICA 79	0.190
ICA 57	ICA 33	0.190
ICA 84	ICA 74	0.190
ICA 56	ICA 85	0.190

Connections		GC
node #1 →	node #2	Strength x10[2]
ICA 82	ICA 75	0.190
ICA 62	ICA 70	0.189
ICA 70	ICA 69	0.189
ICA 98	ICA 39	0.189
ICA 89	ICA 25	0.189
ICA 61	ICA 60	0.189
ICA 86	ICA 71	0.189
ICA 93	ICA 91	0.188
ICA 95	ICA 59	0.188
ICA 59	ICA 33	0.188
ICA 71	ICA 80	0.187
ICA 86	ICA 69	0.187
ICA 88	ICA 94	0.187
ICA 78	ICA 79	0.187
ICA 93	ICA 31	0.186
ICA 23	ICA 29	0.185

Connections		GC
node #1 →	node #2	Strength x10[2]
ICA 64	ICA 67	0.185
ICA 92	ICA 63	0.185
ICA 89	ICA 100	0.185
ICA 71	ICA 70	0.185
ICA 57	ICA 28	0.185
ICA 65	ICA 1	0.185
ICA 87	ICA 99	0.184
ICA 2	ICA 4	0.184
ICA 86	ICA 91	0.184
ICA 99	ICA 30	0.184
ICA 78	ICA 69	0.184
ICA 98	ICA 61	0.184
ICA 63	ICA 30	0.183
ICA 45	ICA 43	0.183
ICA 33	ICA 22	0.183
ICA 97	ICA 81	0.182

Connections		GC
node #1 →	node #2	Strength $x10^2$
ICA 27	ICA 23	0.182
ICA 51	ICA 48	0.182
ICA 98	ICA 67	0.182
ICA 65	ICA 7	0.182
ICA 89	ICA 99	0.182
ICA 69	ICA 77	0.181
ICA 84	ICA 88	0.181
ICA 65	ICA 37	0.181
ICA 78	ICA 76	0.181
ICA 27	ICA 22	0.181
ICA 82	ICA 87	0.180
ICA 55	ICA 29	0.180
ICA 95	ICA 66	0.180
ICA 93	ICA 33	0.180
ICA 76	ICA 73	0.179
ICA 91	ICA 36	0.179

Connections		GC
node #1 →	node #2	Strength $x10^2$
ICA 93	ICA 58	0.179
ICA 55	ICA 59	0.179
ICA 84	ICA 93	0.178
ICA 82	ICA 66	0.178
ICA 4	ICA 85	0.178
ICA 93	ICA 87	0.178
ICA 1	ICA 89	0.177
ICA 84	ICA 85	0.177
ICA 93	ICA 37	0.177
ICA 35	ICA 91	0.177
ICA 73	ICA 94	0.177
ICA 87	ICA 98	0.176
ICA 48	ICA 51	0.176
ICA 66	ICA 87	0.176
ICA 78	ICA 82	0.176
ICA 79	ICA 75	0.176

Connections		GC
node #1 →	node #2	Strength $x10^2$
ICA 6	ICA 64	0.176
ICA 86	ICA 55	0.176
ICA 70	ICA 75	0.176
ICA 73	ICA 67	0.175
ICA 56	ICA 22	0.175
ICA 60	ICA 84	0.174
ICA 34	ICA 28	0.174
ICA 83	ICA 100	0.174
ICA 98	ICA 32	0.174
ICA 58	ICA 95	0.174
ICA 85	ICA 56	0.173
ICA 61	ICA 58	0.173
ICA 94	ICA 38	0.173
ICA 37	ICA 26	0.173
ICA 67	ICA 93	0.173
ICA 64	ICA 59	0.173

Connections		GC
node #1 →	node #2	Strength $x10^2$
ICA 86	ICA 77	0.173
ICA 64	ICA 65	0.173
ICA 61	ICA 92	0.173
ICA 87	ICA 47	0.173
ICA 97	ICA 92	0.173
ICA 86	ICA 67	0.173
ICA 84	ICA 54	0.173
ICA 90	ICA 73	0.172
ICA 98	ICA 99	0.172
ICA 74	ICA 76	0.172
ICA 60	ICA 61	0.172
ICA 65	ICA 95	0.171
ICA 94	ICA 89	0.171
ICA 74	ICA 71	0.171
ICA 63	ICA 29	0.171
ICA 74	ICA 68	0.171

Connections		GC
node #1 →	node #2	Strength $x10^2$
ICA 68	ICA 80	0.171
ICA 27	ICA 21	0.171
ICA 90	ICA 21	0.170
ICA 95	ICA 29	0.170
ICA 93	ICA 42	0.170
ICA 89	ICA 95	0.170
ICA 79	ICA 78	0.169
ICA 65	ICA 58	0.169
ICA 85	ICA 39	0.169
ICA 90	ICA 91	0.169
ICA 82	ICA 30	0.169
ICA 28	ICA 21	0.169
ICA 22	ICA 27	0.169
ICA 74	ICA 99	0.169
ICA 91	ICA 92	0.169
ICA 66	ICA 64	0.168

Connections		GC
node #1 →	node #2	Strength $x10^2$
ICA 91	ICA 60	0.168
ICA 93	ICA 61	0.168
ICA 56	ICA 67	0.168
ICA 56	ICA 86	0.168
ICA 93	ICA 60	0.167
ICA 93	ICA 72	0.167
ICA 26	ICA 37	0.167
ICA 64	ICA 26	0.167
ICA 85	ICA 47	0.167
ICA 89	ICA 66	0.167
ICA 58	ICA 66	0.167
ICA 38	ICA 36	0.166
ICA 23	ICA 30	0.166
ICA 57	ICA 97	0.166
ICA 88	ICA 28	0.165
ICA 64	ICA 88	0.165

Connections		GC
node #1 →	node #2	Strength $x10^2$
ICA 74	ICA 79	0.165
ICA 72	ICA 80	0.165
ICA 56	ICA 21	0.165
ICA 98	ICA 71	0.164
ICA 7	ICA 3	0.164
ICA 81	ICA 61	0.164
ICA 84	ICA 1	0.164
ICA 82	ICA 33	0.164
ICA 67	ICA 45	0.164
ICA 83	ICA 56	0.164
ICA 86	ICA 25	0.163
ICA 84	ICA 98	0.163
ICA 100	ICA 62	0.163
ICA 60	ICA 37	0.163
ICA 84	ICA 70	0.162
ICA 82	ICA 60	0.162

Connections		GC
node #1 →	node #2	Strength $x10^2$
ICA 55	ICA 100	0.162
ICA 86	ICA 83	0.162
ICA 87	ICA 74	0.162
ICA 56	ICA 35	0.162
ICA 87	ICA 31	0.161
ICA 84	ICA 71	0.161
ICA 23	ICA 32	0.161
ICA 57	ICA 55	0.161
ICA 21	ICA 25	0.161
ICA 98	ICA 31	0.160
ICA 91	ICA 35	0.160
ICA 36	ICA 23	0.160
ICA 66	ICA 25	0.160
ICA 86	ICA 47	0.160
ICA 26	ICA 33	0.160
ICA 59	ICA 64	0.160

Connections		GC
node #1 →	node #2	Strength x10^2
ICA 60	ICA 80	0.160
ICA 92	ICA 78	0.160
ICA 60	ICA 55	0.160
ICA 98	ICA 55	0.159
ICA 4	ICA 8	0.159
ICA 62	ICA 32	0.159
ICA 62	ICA 93	0.159
ICA 88	ICA 25	0.159
ICA 57	ICA 30	0.158
ICA 95	ICA 48	0.158
ICA 96	ICA 86	0.158
ICA 62	ICA 100	0.158
ICA 73	ICA 97	0.158
ICA 87	ICA 92	0.158
ICA 93	ICA 30	0.158
ICA 89	ICA 71	0.157

Connections		GC
node #1 →	node #2	Strength x10^2
ICA 89	ICA 93	0.157
ICA 73	ICA 32	0.157
ICA 58	ICA 61	0.157
ICA 87	ICA 90	0.157
ICA 62	ICA 37	0.157
ICA 90	ICA 56	0.157
ICA 89	ICA 76	0.157
ICA 59	ICA 30	0.157
ICA 74	ICA 77	0.157
ICA 98	ICA 87	0.156
ICA 83	ICA 45	0.156
ICA 94	ICA 60	0.156
ICA 37	ICA 30	0.156
ICA 65	ICA 34	0.156
ICA 90	ICA 95	0.156
ICA 95	ICA 45	0.156

Connections		GC
node #1 →	node #2	Strength x10^2
ICA 86	ICA 21	0.156
ICA 71	ICA 77	0.155
ICA 64	ICA 95	0.155
ICA 64	ICA 56	0.155
ICA 65	ICA 82	0.154
ICA 23	ICA 22	0.154
ICA 24	ICA 21	0.154
ICA 85	ICA 57	0.154
ICA 60	ICA 73	0.154
ICA 93	ICA 85	0.154
ICA 90	ICA 77	0.154
ICA 57	ICA 100	0.154
ICA 68	ICA 70	0.154
ICA 94	ICA 54	0.154
ICA 33	ICA 32	0.154
ICA 91	ICA 40	0.153

Connections		GC
node #1 →	node #2	Strength x10^2
ICA 82	ICA 49	0.153
ICA 64	ICA 55	0.153
ICA 25	ICA 22	0.153
ICA 85	ICA 33	0.153
ICA 80	ICA 62	0.153
ICA 93	ICA 34	0.153
ICA 98	ICA 36	0.153
ICA 82	ICA 74	0.153
ICA 94	ICA 34	0.153
ICA 74	ICA 81	0.153
ICA 73	ICA 88	0.153
ICA 30	ICA 21	0.152
ICA 89	ICA 52	0.152
ICA 55	ICA 58	0.152
ICA 61	ICA 33	0.152
ICA 90	ICA 34	0.152

Connections node #1 →	node #2	GC Strength $x10^2$
ICA 65	ICA 24	0.152
ICA 67	ICA 86	0.152
ICA 85	ICA 63	0.152
ICA 84	ICA 96	0.152
ICA 94	ICA 96	0.152
ICA 95	ICA 82	0.152
ICA 85	ICA 76	0.151
ICA 86	ICA 50	0.151
ICA 73	ICA 36	0.151
ICA 91	ICA 87	0.151
ICA 93	ICA 63	0.151
ICA 91	ICA 77	0.151
ICA 72	ICA 73	0.151
ICA 83	ICA 67	0.151
ICA 95	ICA 30	0.151
ICA 93	ICA 64	0.151

Connections node #1 →	node #2	GC Strength $x10^2$
ICA 66	ICA 82	0.151
ICA 91	ICA 88	0.151
ICA 92	ICA 34	0.151
ICA 84	ICA 66	0.150
ICA 84	ICA 83	0.150
ICA 31	ICA 25	0.150
ICA 83	ICA 72	0.150
ICA 87	ICA 65	0.150
ICA 73	ICA 99	0.150
ICA 67	ICA 46	0.150
ICA 65	ICA 57	0.150
ICA 63	ICA 85	0.150
ICA 85	ICA 93	0.149
ICA 83	ICA 35	0.149
ICA 29	ICA 42	0.149
ICA 69	ICA 70	0.149

Connections node #1 →	node #2	GC Strength $x10^2$
ICA 81	ICA 77	0.149
ICA 93	ICA 75	0.149
ICA 32	ICA 88	0.148
ICA 73	ICA 37	0.148
ICA 83	ICA 52	0.148
ICA 64	ICA 77	0.148
ICA 34	ICA 25	0.148
ICA 93	ICA 86	0.148
ICA 93	ICA 80	0.147
ICA 71	ICA 79	0.147
ICA 75	ICA 72	0.147
ICA 87	ICA 61	0.147
ICA 100	ICA 72	0.147
ICA 58	ICA 63	0.147
ICA 55	ICA 25	0.147
ICA 62	ICA 82	0.146

Connections node #1 →	node #2	GC Strength $x10^2$
ICA 88	ICA 64	0.146
ICA 96	ICA 95	0.146
ICA 58	ICA 87	0.146
ICA 6	ICA 2	0.146
ICA 57	ICA 96	0.145
ICA 70	ICA 38	0.145
ICA 66	ICA 43	0.145
ICA 92	ICA 88	0.145
ICA 56	ICA 100	0.145
ICA 36	ICA 4	0.145
ICA 73	ICA 33	0.145
ICA 87	ICA 93	0.145
ICA 83	ICA 97	0.145
ICA 74	ICA 72	0.145
ICA 95	ICA 23	0.145
ICA 96	ICA 31	0.145

Connections		GC
node #1 →	node #2	Strength $x10^2$
ICA 62	ICA 86	0.144
ICA 64	ICA 6	0.144
ICA 97	ICA 36	0.144
ICA 97	ICA 95	0.144
ICA 99	ICA 95	0.144
ICA 25	ICA 24	0.144
ICA 98	ICA 92	0.144
ICA 34	ICA 42	0.144
ICA 66	ICA 2	0.144
ICA 86	ICA 100	0.143
ICA 84	ICA 26	0.143
ICA 83	ICA 82	0.143
ICA 86	ICA 59	0.143
ICA 22	ICA 33	0.143
ICA 63	ICA 92	0.143
ICA 93	ICA 100	0.143

Connections		GC
node #1 →	node #2	Strength $x10^2$
ICA 69	ICA 94	0.143
ICA 62	ICA 79	0.143
ICA 98	ICA 57	0.143
ICA 65	ICA 60	0.143
ICA 62	ICA 33	0.143
ICA 92	ICA 97	0.143
ICA 59	ICA 86	0.143
ICA 66	ICA 60	0.143
ICA 94	ICA 90	0.143
ICA 85	ICA 80	0.142
ICA 58	ICA 38	0.142
ICA 97	ICA 84	0.142
ICA 93	ICA 5	0.142
ICA 30	ICA 24	0.142
ICA 57	ICA 60	0.142
ICA 60	ICA 57	0.142

Connections		GC
node #1 →	node #2	Strength $x10^2$
ICA 82	ICA 97	0.142
ICA 90	ICA 87	0.141
ICA 66	ICA 89	0.141
ICA 66	ICA 28	0.141
ICA 85	ICA 95	0.141
ICA 92	ICA 58	0.141
ICA 58	ICA 1	0.141
ICA 88	ICA 89	0.141
ICA 60	ICA 87	0.141
ICA 66	ICA 58	0.141
ICA 96	ICA 94	0.141
ICA 86	ICA 30	0.141
ICA 56	ICA 83	0.140
ICA 6	ICA 33	0.140
ICA 91	ICA 58	0.140
ICA 73	ICA 42	0.140

Connections		GC
node #1 →	node #2	Strength $x10^2$
ICA 94	ICA 49	0.140
ICA 66	ICA 37	0.140
ICA 98	ICA 49	0.140
ICA 93	ICA 99	0.139
ICA 77	ICA 81	0.139
ICA 93	ICA 62	0.139
ICA 67	ICA 88	0.139
ICA 36	ICA 85	0.139
ICA 66	ICA 1	0.139
ICA 83	ICA 88	0.139
ICA 70	ICA 78	0.139
ICA 77	ICA 80	0.138
ICA 94	ICA 68	0.138
ICA 84	ICA 62	0.138
ICA 6	ICA 89	0.138
ICA 83	ICA 76	0.138

Connections		GC
node #1 →	node #2	Strength x10^2
ICA 38	ICA 24	0.138
ICA 55	ICA 31	0.137
ICA 89	ICA 88	0.137
ICA 62	ICA 92	0.137
ICA 2	ICA 3	0.137
ICA 66	ICA 23	0.137
ICA 100	ICA 86	0.137
ICA 94	ICA 31	0.137
ICA 46	ICA 23	0.137
ICA 26	ICA 24	0.136
ICA 68	ICA 81	0.136
ICA 61	ICA 34	0.136
ICA 58	ICA 82	0.136
ICA 57	ICA 7	0.136
ICA 100	ICA 57	0.136
ICA 85	ICA 73	0.136

Connections		GC
node #1 →	node #2	Strength x10^2
ICA 92	ICA 98	0.136
ICA 67	ICA 82	0.136
ICA 92	ICA 71	0.136
ICA 58	ICA 84	0.136
ICA 64	ICA 90	0.136
ICA 77	ICA 99	0.135
ICA 78	ICA 74	0.135
ICA 86	ICA 23	0.135
ICA 4	ICA 82	0.135
ICA 94	ICA 43	0.135
ICA 4	ICA 36	0.135
ICA 55	ICA 35	0.135
ICA 84	ICA 95	0.135
ICA 100	ICA 47	0.135
ICA 6	ICA 4	0.135
ICA 99	ICA 77	0.134

Connections		GC
node #1 →	node #2	Strength x10^2
ICA 88	ICA 21	0.134
ICA 83	ICA 26	0.134
ICA 83	ICA 24	0.134
ICA 84	ICA 65	0.134
ICA 73	ICA 100	0.134
ICA 21	ICA 30	0.134
ICA 24	ICA 90	0.134
ICA 69	ICA 84	0.133
ICA 26	ICA 32	0.133
ICA 80	ICA 73	0.133
ICA 36	ICA 34	0.133
ICA 85	ICA 100	0.133
ICA 97	ICA 23	0.133
ICA 99	ICA 82	0.133
ICA 28	ICA 25	0.133
ICA 70	ICA 91	0.132

Connections		GC
node #1 →	node #2	Strength x10^2
ICA 64	ICA 71	0.132
ICA 62	ICA 64	0.132
ICA 94	ICA 69	0.132
ICA 67	ICA 64	0.132
ICA 2	ICA 23	0.132
ICA 38	ICA 23	0.132
ICA 95	ICA 87	0.132
ICA 94	ICA 57	0.132
ICA 40	ICA 94	0.132
ICA 84	ICA 82	0.132
ICA 46	ICA 48	0.132
ICA 83	ICA 78	0.132
ICA 57	ICA 93	0.132
ICA 28	ICA 42	0.132
ICA 62	ICA 40	0.132
ICA 33	ICA 26	0.132

Connections		GC
node #1 →	node #2	Strength $x10^2$
ICA 3	ICA 94	0.131
ICA 92	ICA 72	0.131
ICA 93	ICA 21	0.131
ICA 90	ICA 25	0.131
ICA 83	ICA 51	0.131
ICA 57	ICA 94	0.131
ICA 93	ICA 90	0.131
ICA 82	ICA 94	0.131
ICA 64	ICA 81	0.131
ICA 60	ICA 25	0.131
ICA 65	ICA 55	0.131
ICA 86	ICA 90	0.131
ICA 84	ICA 67	0.131
ICA 61	ICA 93	0.131
ICA 67	ICA 35	0.131
ICA 83	ICA 46	0.131

Connections		GC
node #1 →	node #2	Strength $x10^2$
ICA 2	ICA 57	0.131
ICA 94	ICA 76	0.131
ICA 39	ICA 52	0.130
ICA 85	ICA 32	0.130
ICA 93	ICA 94	0.130
ICA 82	ICA 65	0.130
ICA 24	ICA 23	0.130
ICA 29	ICA 31	0.130
ICA 77	ICA 57	0.130
ICA 93	ICA 22	0.129
ICA 64	ICA 99	0.129
ICA 95	ICA 86	0.129
ICA 60	ICA 50	0.129
ICA 36	ICA 27	0.129
ICA 89	ICA 84	0.129
ICA 83	ICA 54	0.129

Connections		GC
node #1 →	node #2	Strength $x10^2$
ICA 46	ICA 44	0.129
ICA 9	ICA 72	0.129
ICA 77	ICA 69	0.129
ICA 74	ICA 100	0.128
ICA 88	ICA 55	0.128
ICA 68	ICA 74	0.128
ICA 86	ICA 7	0.128
ICA 67	ICA 44	0.128
ICA 80	ICA 35	0.128
ICA 35	ICA 84	0.128
ICA 56	ICA 42	0.127
ICA 57	ICA 72	0.127
ICA 83	ICA 37	0.127
ICA 66	ICA 86	0.127
ICA 93	ICA 65	0.127
ICA 80	ICA 88	0.127

Connections		GC
node #1 →	node #2	Strength $x10^2$
ICA 70	ICA 74	0.127
ICA 51	ICA 63	0.127
ICA 28	ICA 34	0.127
ICA 86	ICA 87	0.127
ICA 45	ICA 38	0.127
ICA 92	ICA 100	0.126
ICA 98	ICA 60	0.126
ICA 83	ICA 40	0.126
ICA 65	ICA 74	0.126
ICA 89	ICA 42	0.126
ICA 73	ICA 57	0.126
ICA 65	ICA 31	0.126
ICA 97	ICA 57	0.126
ICA 36	ICA 38	0.126
ICA 87	ICA 85	0.126
ICA 13	ICA 36	0.126

Connections: node #1 →	node #2	GC: Strength $x10^2$
ICA 70	ICA 77	0.126
ICA 95	ICA 83	0.126
ICA 90	ICA 93	0.126
ICA 89	ICA 39	0.125
ICA 93	ICA 27	0.125
ICA 89	ICA 65	0.125
ICA 88	ICA 22	0.125
ICA 56	ICA 94	0.125
ICA 66	ICA 34	0.125
ICA 52	ICA 39	0.125
ICA 97	ICA 38	0.125
ICA 86	ICA 82	0.125
ICA 65	ICA 94	0.125
ICA 77	ICA 74	0.125
ICA 60	ICA 69	0.125
ICA 63	ICA 66	0.125

Connections: node #1 →	node #2	GC: Strength $x10^2$
ICA 96	ICA 88	0.125
ICA 89	ICA 73	0.125
ICA 60	ICA 38	0.125
ICA 64	ICA 21	0.125
ICA 95	ICA 90	0.125
ICA 25	ICA 89	0.124
ICA 30	ICA 25	0.124
ICA 73	ICA 35	0.124
ICA 67	ICA 98	0.124
ICA 82	ICA 70	0.124
ICA 100	ICA 99	0.124
ICA 100	ICA 21	0.124
ICA 89	ICA 57	0.124
ICA 80	ICA 70	0.124
ICA 83	ICA 96	0.124
ICA 45	ICA 35	0.124

Connections: node #1 →	node #2	GC: Strength $x10^2$
ICA 89	ICA 63	0.124
ICA 72	ICA 75	0.124
ICA 80	ICA 76	0.124
ICA 3	ICA 88	0.124
ICA 73	ICA 78	0.124
ICA 62	ICA 85	0.124
ICA 91	ICA 56	0.124
ICA 48	ICA 46	0.123
ICA 66	ICA 56	0.123
ICA 21	ICA 60	0.123
ICA 64	ICA 60	0.123
ICA 81	ICA 76	0.123
ICA 91	ICA 93	0.123
ICA 94	ICA 88	0.123
ICA 73	ICA 75	0.123
ICA 67	ICA 84	0.123

Connections: node #1 →	node #2	GC: Strength $x10^2$
ICA 55	ICA 63	0.123
ICA 88	ICA 67	0.123
ICA 64	ICA 42	0.123
ICA 58	ICA 47	0.123
ICA 100	ICA 24	0.123
ICA 99	ICA 26	0.123
ICA 79	ICA 70	0.123
ICA 58	ICA 33	0.122
ICA 65	ICA 92	0.122
ICA 59	ICA 1	0.122
ICA 74	ICA 95	0.122
ICA 22	ICA 25	0.122
ICA 73	ICA 72	0.122
ICA 58	ICA 89	0.122
ICA 36	ICA 35	0.122
ICA 64	ICA 63	0.122

Connections		GC
node #1 →	node #2	Strength x10^2
ICA 58	ICA 32	0.122
ICA 26	ICA 30	0.122
ICA 6	ICA 59	0.122
ICA 90	ICA 89	0.122
ICA 88	ICA 77	0.122
ICA 31	ICA 60	0.122
ICA 91	ICA 25	0.121
ICA 85	ICA 46	0.121
ICA 74	ICA 78	0.121
ICA 63	ICA 57	0.121
ICA 29	ICA 47	0.121
ICA 86	ICA 73	0.121
ICA 67	ICA 81	0.121
ICA 65	ICA 85	0.121
ICA 65	ICA 87	0.121
ICA 81	ICA 75	0.121

Connections		GC
node #1 →	node #2	Strength x10^2
ICA 82	ICA 62	0.121
ICA 87	ICA 62	0.121
ICA 99	ICA 81	0.121
ICA 97	ICA 26	0.121
ICA 63	ICA 77	0.121
ICA 80	ICA 98	0.121
ICA 25	ICA 30	0.121
ICA 76	ICA 81	0.121
ICA 64	ICA 36	0.121
ICA 96	ICA 23	0.120
ICA 22	ICA 85	0.120
ICA 65	ICA 81	0.120
ICA 74	ICA 75	0.120
ICA 92	ICA 91	0.120
ICA 66	ICA 40	0.120
ICA 4	ICA 77	0.120

Connections		GC
node #1 →	node #2	Strength x10^2
ICA 73	ICA 59	0.120
ICA 89	ICA 77	0.120
ICA 69	ICA 56	0.120
ICA 92	ICA 45	0.120
ICA 100	ICA 78	0.120
ICA 97	ICA 56	0.120
ICA 56	ICA 7	0.119
ICA 35	ICA 55	0.119
ICA 83	ICA 73	0.119
ICA 74	ICA 97	0.119
ICA 55	ICA 88	0.119
ICA 82	ICA 7	0.119
ICA 88	ICA 81	0.119
ICA 60	ICA 35	0.119
ICA 64	ICA 97	0.119
ICA 67	ICA 32	0.119

Connections		GC
node #1 →	node #2	Strength x10^2
ICA 86	ICA 85	0.119
ICA 67	ICA 40	0.119
ICA 60	ICA 40	0.119
ICA 64	ICA 51	0.119
ICA 64	ICA 69	0.119
ICA 28	ICA 92	0.119
ICA 86	ICA 93	0.119
ICA 44	ICA 53	0.119
ICA 94	ICA 72	0.119
ICA 99	ICA 84	0.119
ICA 82	ICA 72	0.119
ICA 96	ICA 9	0.118
ICA 61	ICA 27	0.118
ICA 67	ICA 83	0.118
ICA 63	ICA 98	0.118
ICA 36	ICA 95	0.118

Connections		GC
node #1 →	node #2	Strength x10^2
ICA 82	ICA 81	0.118
ICA 60	ICA 94	0.118
ICA 62	ICA 69	0.118
ICA 57	ICA 25	0.118
ICA 91	ICA 31	0.118
ICA 88	ICA 27	0.118
ICA 87	ICA 29	0.118
ICA 59	ICA 100	0.118
ICA 66	ICA 65	0.118
ICA 83	ICA 98	0.118
ICA 76	ICA 79	0.118
ICA 59	ICA 57	0.118
ICA 85	ICA 87	0.118
ICA 82	ICA 4	0.118
ICA 82	ICA 99	0.118
ICA 100	ICA 42	0.118

Connections		GC
node #1 →	node #2	Strength x10^2
ICA 36	ICA 91	0.117
ICA 95	ICA 27	0.117
ICA 87	ICA 21	0.117
ICA 89	ICA 82	0.117
ICA 83	ICA 58	0.117
ICA 64	ICA 91	0.117
ICA 93	ICA 6	0.117
ICA 57	ICA 87	0.117
ICA 35	ICA 81	0.117
ICA 95	ICA 71	0.117
ICA 97	ICA 91	0.117
ICA 94	ICA 84	0.117
ICA 89	ICA 22	0.117
ICA 87	ICA 56	0.117
ICA 23	ICA 38	0.117
ICA 99	ICA 65	0.117

Connections		GC
node #1 →	node #2	Strength x10^2
ICA 77	ICA 31	0.117
ICA 2	ICA 27	0.117
ICA 58	ICA 31	0.117
ICA 96	ICA 48	0.116
ICA 56	ICA 62	0.116
ICA 88	ICA 73	0.116
ICA 83	ICA 43	0.116
ICA 56	ICA 97	0.116
ICA 86	ICA 29	0.116
ICA 64	ICA 73	0.116
ICA 44	ICA 48	0.116
ICA 82	ICA 79	0.116
ICA 95	ICA 69	0.116
ICA 65	ICA 93	0.116
ICA 57	ICA 67	0.116
ICA 66	ICA 38	0.116

Connections		GC
node #1 →	node #2	Strength x10^2
ICA 29	ICA 34	0.116
ICA 89	ICA 31	0.115
ICA 62	ICA 55	0.115
ICA 63	ICA 81	0.115
ICA 55	ICA 62	0.115
ICA 77	ICA 71	0.115
ICA 48	ICA 91	0.115
ICA 96	ICA 57	0.115
ICA 85	ICA 34	0.115
ICA 93	ICA 78	0.115
ICA 78	ICA 70	0.115
ICA 21	ICA 91	0.115
ICA 56	ICA 59	0.115
ICA 89	ICA 68	0.115
ICA 81	ICA 66	0.115
ICA 59	ICA 95	0.115

Connections		GC
node #1 →	node #2	Strength x10^2
ICA 32	ICA 38	0.115
ICA 79	ICA 71	0.115
ICA 13	ICA 4	0.114
ICA 92	ICA 87	0.114
ICA 96	ICA 44	0.114
ICA 85	ICA 65	0.114
ICA 89	ICA 90	0.114
ICA 63	ICA 80	0.114
ICA 91	ICA 28	0.114
ICA 61	ICA 85	0.114
ICA 37	ICA 31	0.114
ICA 73	ICA 54	0.114
ICA 81	ICA 87	0.114
ICA 64	ICA 34	0.114
ICA 10	ICA 4	0.114
ICA 27	ICA 25	0.114

Connections		GC
node #1 →	node #2	Strength x10^2
ICA 72	ICA 77	0.114
ICA 83	ICA 21	0.114
ICA 94	ICA 58	0.114
ICA 49	ICA 82	0.114
ICA 92	ICA 96	0.114
ICA 88	ICA 96	0.114
ICA 83	ICA 84	0.114
ICA 93	ICA 25	0.114
ICA 64	ICA 74	0.114
ICA 3	ICA 85	0.114
ICA 58	ICA 67	0.114
ICA 89	ICA 86	0.114
ICA 31	ICA 82	0.114
ICA 69	ICA 81	0.114
ICA 96	ICA 80	0.114
ICA 88	ICA 62	0.114

Connections		GC
node #1 →	node #2	Strength x10^2
ICA 76	ICA 69	0.113
ICA 23	ICA 45	0.113
ICA 81	ICA 74	0.113
ICA 48	ICA 95	0.113
ICA 84	ICA 94	0.113
ICA 89	ICA 23	0.113
ICA 63	ICA 41	0.113
ICA 100	ICA 59	0.113
ICA 90	ICA 51	0.113
ICA 58	ICA 7	0.113
ICA 89	ICA 60	0.113
ICA 89	ICA 34	0.113
ICA 29	ICA 93	0.113
ICA 56	ICA 80	0.112
ICA 40	ICA 52	0.112
ICA 75	ICA 71	0.112

Connections		GC
node #1 →	node #2	Strength x10^2
ICA 100	ICA 77	0.112
ICA 90	ICA 76	0.112
ICA 82	ICA 36	0.112
ICA 23	ICA 25	0.112
ICA 72	ICA 78	0.112
ICA 46	ICA 54	0.112
ICA 95	ICA 54	0.112
ICA 73	ICA 23	0.112
ICA 57	ICA 86	0.112
ICA 76	ICA 70	0.112
ICA 98	ICA 82	0.112
ICA 33	ICA 42	0.112
ICA 73	ICA 26	0.112
ICA 51	ICA 49	0.112
ICA 93	ICA 70	0.112
ICA 69	ICA 62	0.112

Connections		GC
node #1 →	node #2	Strength $x10^2$
ICA 91	ICA 84	0.112
ICA 94	ICA 100	0.111
ICA 99	ICA 58	0.111
ICA 82	ICA 27	0.111
ICA 58	ICA 51	0.111
ICA 50	ICA 31	0.111
ICA 84	ICA 64	0.111
ICA 90	ICA 28	0.111
ICA 63	ICA 21	0.111
ICA 99	ICA 89	0.111
ICA 21	ICA 92	0.111
ICA 81	ICA 99	0.111
ICA 99	ICA 28	0.111
ICA 62	ICA 25	0.111
ICA 98	ICA 25	0.111
ICA 60	ICA 31	0.111

Connections		GC
node #1 →	node #2	Strength $x10^2$
ICA 74	ICA 83	0.111
ICA 24	ICA 26	0.111
ICA 62	ICA 72	0.111
ICA 62	ICA 71	0.111
ICA 87	ICA 66	0.111
ICA 71	ICA 51	0.111
ICA 84	ICA 28	0.111
ICA 100	ICA 91	0.111
ICA 88	ICA 97	0.110
ICA 96	ICA 36	0.110
ICA 86	ICA 22	0.110
ICA 64	ICA 43	0.110
ICA 87	ICA 64	0.110
ICA 66	ICA 24	0.110
ICA 66	ICA 31	0.110
ICA 90	ICA 65	0.110

Connections		GC
node #1 →	node #2	Strength $x10^2$
ICA 82	ICA 93	0.110
ICA 87	ICA 91	0.110
ICA 64	ICA 82	0.110
ICA 61	ICA 31	0.110
ICA 80	ICA 91	0.110
ICA 73	ICA 27	0.110
ICA 98	ICA 94	0.110
ICA 94	ICA 65	0.110
ICA 80	ICA 51	0.110
ICA 25	ICA 34	0.110
ICA 18	ICA 99	0.110
ICA 80	ICA 94	0.110
ICA 86	ICA 81	0.109
ICA 82	ICA 71	0.109
ICA 34	ICA 29	0.109
ICA 94	ICA 82	0.109

Connections		GC
node #1 →	node #2	Strength $x10^2$
ICA 64	ICA 54	0.109
ICA 88	ICA 43	0.109
ICA 56	ICA 95	0.109
ICA 69	ICA 71	0.109
ICA 67	ICA 72	0.109
ICA 60	ICA 89	0.109
ICA 83	ICA 62	0.109
ICA 69	ICA 73	0.109
ICA 99	ICA 33	0.109
ICA 72	ICA 79	0.109
ICA 56	ICA 82	0.109
ICA 63	ICA 84	0.109
ICA 95	ICA 77	0.109
ICA 89	ICA 67	0.109
ICA 78	ICA 81	0.109
ICA 23	ICA 26	0.109

Connections		GC
node #1 →	node #2	Strength $x10^2$
ICA 57	ICA 89	0.109
ICA 81	ICA 58	0.109
ICA 99	ICA 83	0.109
ICA 94	ICA 48	0.109
ICA 62	ICA 77	0.108
ICA 28	ICA 63	0.108
ICA 80	ICA 72	0.108
ICA 88	ICA 37	0.108
ICA 74	ICA 84	0.108
ICA 91	ICA 81	0.108
ICA 98	ICA 96	0.108
ICA 87	ICA 51	0.108
ICA 93	ICA 35	0.108
ICA 5	ICA 93	0.108
ICA 98	ICA 77	0.108
ICA 97	ICA 85	0.108

Connections		GC
node #1 →	node #2	Strength $x10^2$
ICA 57	ICA 29	0.108
ICA 72	ICA 82	0.108
ICA 47	ICA 29	0.108
ICA 76	ICA 64	0.108
ICA 36	ICA 63	0.108
ICA 71	ICA 4	0.108
ICA 7	ICA 79	0.108
ICA 56	ICA 47	0.108
ICA 75	ICA 74	0.108
ICA 93	ICA 3	0.108
ICA 58	ICA 30	0.108
ICA 83	ICA 95	0.108
ICA 84	ICA 72	0.108
ICA 58	ICA 55	0.108
ICA 98	ICA 6	0.108
ICA 86	ICA 94	0.108

Connections		GC
node #1 →	node #2	Strength $x10^2$
ICA 52	ICA 40	0.108
ICA 31	ICA 58	0.107
ICA 66	ICA 84	0.107
ICA 35	ICA 61	0.107
ICA 60	ICA 96	0.107
ICA 74	ICA 51	0.107
ICA 9	ICA 10	0.107
ICA 53	ICA 44	0.107
ICA 40	ICA 36	0.107
ICA 76	ICA 87	0.107
ICA 87	ICA 88	0.107
ICA 61	ICA 28	0.107
ICA 65	ICA 47	0.107
ICA 42	ICA 29	0.107
ICA 86	ICA 65	0.107
ICA 59	ICA 61	0.107

Connections		GC
node #1 →	node #2	Strength $x10^2$
ICA 31	ICA 50	0.107
ICA 23	ICA 48	0.107
ICA 100	ICA 82	0.107
ICA 88	ICA 72	0.107
ICA 56	ICA 3	0.107
ICA 83	ICA 75	0.107
ICA 24	ICA 27	0.107
ICA 93	ICA 36	0.107
ICA 37	ICA 91	0.107
ICA 2	ICA 59	0.107
ICA 81	ICA 82	0.107
ICA 77	ICA 87	0.107
ICA 65	ICA 30	0.107
ICA 83	ICA 86	0.107
ICA 68	ICA 72	0.107
ICA 58	ICA 81	0.107

Connections		GC
node #1 →	node #2	Strength x10^2
ICA 88	ICA 83	0.107
ICA 96	ICA 92	0.107
ICA 82	ICA 47	0.107
ICA 42	ICA 94	0.107
ICA 74	ICA 36	0.107
ICA 72	ICA 93	0.107
ICA 79	ICA 76	0.107
ICA 92	ICA 42	0.106
ICA 63	ICA 70	0.106
ICA 4	ICA 6	0.106
ICA 62	ICA 87	0.106
ICA 58	ICA 26	0.106
ICA 82	ICA 31	0.106
ICA 56	ICA 79	0.106
ICA 38	ICA 32	0.106
ICA 56	ICA 98	0.106

Connections		GC
node #1 →	node #2	Strength x10^2
ICA 76	ICA 84	0.106
ICA 55	ICA 30	0.106
ICA 100	ICA 22	0.106
ICA 59	ICA 81	0.106
ICA 60	ICA 62	0.106
ICA 4	ICA 72	0.106
ICA 86	ICA 37	0.106
ICA 61	ICA 30	0.106
ICA 62	ICA 27	0.106
ICA 72	ICA 69	0.106
ICA 30	ICA 37	0.106
ICA 7	ICA 4	0.106
ICA 64	ICA 22	0.106
ICA 92	ICA 80	0.106
ICA 36	ICA 61	0.106
ICA 90	ICA 79	0.106

Connections		GC
node #1 →	node #2	Strength x10^2
ICA 59	ICA 60	0.106
ICA 87	ICA 57	0.106
ICA 61	ICA 56	0.106
ICA 98	ICA 65	0.106
ICA 85	ICA 36	0.105
ICA 80	ICA 96	0.105
ICA 74	ICA 57	0.105
ICA 67	ICA 51	0.105
ICA 56	ICA 60	0.105
ICA 75	ICA 70	0.105
ICA 42	ICA 33	0.105
ICA 95	ICA 24	0.105
ICA 67	ICA 38	0.105
ICA 84	ICA 77	0.105
ICA 73	ICA 68	0.105
ICA 65	ICA 77	0.105

Connections		GC
node #1 →	node #2	Strength x10^2
ICA 60	ICA 66	0.105
ICA 64	ICA 84	0.105
ICA 59	ICA 77	0.105
ICA 95	ICA 26	0.105
ICA 100	ICA 65	0.105
ICA 60	ICA 21	0.105
ICA 57	ICA 82	0.105
ICA 22	ICA 37	0.105
ICA 83	ICA 87	0.105
ICA 85	ICA 23	0.105
ICA 85	ICA 67	0.105
ICA 75	ICA 82	0.105
ICA 85	ICA 6	0.105
ICA 57	ICA 37	0.105
ICA 29	ICA 28	0.104
ICA 64	ICA 40	0.104

Connections		GC
node #1 →	node #2	Strength x10^2
ICA 4	ICA 100	0.104
ICA 90	ICA 81	0.104
ICA 77	ICA 73	0.104
ICA 99	ICA 2	0.104
ICA 91	ICA 42	0.104
ICA 99	ICA 98	0.104
ICA 96	ICA 82	0.104
ICA 7	ICA 84	0.104
ICA 89	ICA 58	0.104
ICA 97	ICA 83	0.104
ICA 98	ICA 22	0.104
ICA 85	ICA 27	0.104
ICA 68	ICA 82	0.104
ICA 84	ICA 89	0.104
ICA 55	ICA 24	0.104
ICA 1	ICA 55	0.104

Connections		GC
node #1 →	node #2	Strength x10^2
ICA 63	ICA 55	0.104
ICA 56	ICA 30	0.104
ICA 100	ICA 23	0.104
ICA 87	ICA 54	0.104
ICA 1	ICA 59	0.104
ICA 66	ICA 57	0.104
ICA 64	ICA 87	0.104
ICA 59	ICA 56	0.104
ICA 100	ICA 38	0.104
ICA 100	ICA 63	0.104
ICA 57	ICA 85	0.104
ICA 47	ICA 81	0.104
ICA 92	ICA 84	0.104
ICA 70	ICA 80	0.104
ICA 95	ICA 76	0.104
ICA 66	ICA 93	0.104

Connections		GC
node #1 →	node #2	Strength x10^2
ICA 93	ICA 66	0.104
ICA 98	ICA 80	0.104
ICA 38	ICA 34	0.104
ICA 58	ICA 37	0.104
ICA 65	ICA 27	0.104
ICA 63	ICA 64	0.104
ICA 66	ICA 100	0.104
ICA 78	ICA 72	0.104
ICA 52	ICA 89	0.103
ICA 84	ICA 22	0.103
ICA 65	ICA 63	0.103
ICA 7	ICA 50	0.103
ICA 55	ICA 37	0.103
ICA 57	ICA 83	0.103
ICA 36	ICA 98	0.103
ICA 95	ICA 85	0.103

Connections		GC
node #1 →	node #2	Strength x10^2
ICA 95	ICA 55	0.103
ICA 73	ICA 79	0.103
ICA 72	ICA 57	0.103
ICA 95	ICA 44	0.103
ICA 64	ICA 47	0.103
ICA 60	ICA 24	0.103
ICA 60	ICA 46	0.103
ICA 86	ICA 62	0.103
ICA 83	ICA 71	0.103
ICA 64	ICA 57	0.103
ICA 96	ICA 50	0.103
ICA 23	ICA 46	0.103
ICA 88	ICA 32	0.103
ICA 70	ICA 76	0.103
ICA 85	ICA 35	0.103
ICA 97	ICA 53	0.103

Connections		GC
node #1 →	node #2	Strength $x10^2$
ICA 80	ICA 74	0.103
ICA 8	ICA 67	0.103
ICA 81	ICA 27	0.103
ICA 60	ICA 71	0.103
ICA 93	ICA 96	0.103
ICA 89	ICA 26	0.103
ICA 99	ICA 27	0.103
ICA 90	ICA 86	0.103
ICA 100	ICA 73	0.103
ICA 64	ICA 85	0.102
ICA 92	ICA 67	0.102
ICA 98	ICA 91	0.102
ICA 56	ICA 91	0.102
ICA 36	ICA 37	0.102
ICA 69	ICA 90	0.102
ICA 63	ICA 74	0.102

Connections		GC
node #1 →	node #2	Strength $x10^2$
ICA 66	ICA 27	0.102
ICA 67	ICA 23	0.102
ICA 88	ICA 26	0.102
ICA 92	ICA 25	0.102
ICA 89	ICA 45	0.102
ICA 100	ICA 98	0.102
ICA 68	ICA 94	0.102
ICA 86	ICA 95	0.102
ICA 10	ICA 9	0.102
ICA 90	ICA 36	0.102
ICA 70	ICA 4	0.102
ICA 66	ICA 68	0.102
ICA 62	ICA 89	0.102
ICA 100	ICA 66	0.102
ICA 73	ICA 29	0.102
ICA 62	ICA 73	0.102

Connections		GC
node #1 →	node #2	Strength $x10^2$
ICA 55	ICA 28	0.102
ICA 100	ICA 79	0.102
ICA 31	ICA 85	0.102
ICA 43	ICA 45	0.102
ICA 90	ICA 68	0.102
ICA 63	ICA 1	0.102
ICA 72	ICA 62	0.102
ICA 88	ICA 31	0.102
ICA 86	ICA 35	0.102
ICA 78	ICA 71	0.102
ICA 25	ICA 31	0.101
ICA 77	ICA 85	0.101
ICA 23	ICA 36	0.101
ICA 100	ICA 74	0.101
ICA 95	ICA 70	0.101
ICA 86	ICA 1	0.101

Connections		GC
node #1 →	node #2	Strength $x10^2$
ICA 87	ICA 72	0.101
ICA 63	ICA 86	0.101
ICA 67	ICA 58	0.101
ICA 64	ICA 86	0.101
ICA 67	ICA 89	0.101
ICA 62	ICA 23	0.101
ICA 58	ICA 35	0.101
ICA 96	ICA 53	0.101
ICA 85	ICA 61	0.101
ICA 46	ICA 35	0.101
ICA 66	ICA 35	0.101
ICA 48	ICA 23	0.101
ICA 95	ICA 73	0.101
ICA 80	ICA 58	0.101
ICA 56	ICA 73	0.101
ICA 89	ICA 27	0.101

Connections		GC
node #1 →	node #2	Strength x10^2
ICA 82	ICA 86	0.101
ICA 99	ICA 100	0.101
ICA 72	ICA 68	0.101
ICA 28	ICA 50	0.101
ICA 89	ICA 69	0.101
ICA 85	ICA 40	0.101
ICA 66	ICA 94	0.101
ICA 48	ICA 24	0.101
ICA 83	ICA 49	0.101
ICA 62	ICA 65	0.101
ICA 92	ICA 31	0.101
ICA 67	ICA 73	0.101
ICA 25	ICA 57	0.101
ICA 100	ICA 71	0.101
ICA 55	ICA 64	0.101
ICA 95	ICA 74	0.101

Connections		GC
node #1 →	node #2	Strength x10^2
ICA 21	ICA 66	0.101
ICA 24	ICA 63	0.101
ICA 37	ICA 22	0.101
ICA 89	ICA 6	0.100
ICA 73	ICA 69	0.100
ICA 80	ICA 68	0.100
ICA 57	ICA 98	0.100
ICA 70	ICA 23	0.100
ICA 36	ICA 100	0.100
ICA 59	ICA 91	0.100
ICA 69	ICA 65	0.100
ICA 66	ICA 22	0.100
ICA 89	ICA 43	0.100
ICA 95	ICA 80	0.100
ICA 92	ICA 70	0.100
ICA 21	ICA 55	0.100

Connections		GC
node #1 →	node #2	Strength x10^2
ICA 58	ICA 92	0.100
ICA 82	ICA 29	0.100
ICA 97	ICA 73	0.100
ICA 90	ICA 45	0.100
ICA 91	ICA 1	0.100
ICA 93	ICA 77	0.100
ICA 58	ICA 99	0.100
ICA 25	ICA 90	0.100
ICA 86	ICA 51	0.100
ICA 66	ICA 63	0.100
ICA 100	ICA 88	0.100
ICA 90	ICA 37	0.100
ICA 96	ICA 54	0.100
ICA 62	ICA 34	0.100
ICA 84	ICA 97	0.100
ICA 85	ICA 68	0.100

Connections		GC
node #1 →	node #2	Strength x10^2
ICA 33	ICA 25	0.100
ICA 93	ICA 57	0.100
ICA 84	ICA 27	0.100
ICA 82	ICA 23	0.100
ICA 23	ICA 82	0.100
ICA 66	ICA 50	0.100
ICA 63	ICA 91	0.099
ICA 87	ICA 39	0.099
ICA 82	ICA 85	0.099
ICA 56	ICA 29	0.099
ICA 77	ICA 59	0.099
ICA 83	ICA 81	0.099
ICA 98	ICA 52	0.099
ICA 93	ICA 32	0.099
ICA 51	ICA 54	0.099
ICA 88	ICA 75	0.099

Connections node #1	→ node #2	GC Strength $x10^2$
ICA 1	ICA 33	0.099
ICA 82	ICA 92	0.099
ICA 93	ICA 97	0.099
ICA 89	ICA 91	0.099
ICA 67	ICA 53	0.099
ICA 64	ICA 33	0.099
ICA 61	ICA 80	0.099
ICA 92	ICA 24	0.099
ICA 53	ICA 48	0.099
ICA 100	ICA 31	0.099
ICA 73	ICA 83	0.099
ICA 63	ICA 69	0.099
ICA 82	ICA 38	0.099
ICA 63	ICA 32	0.099
ICA 82	ICA 39	0.099
ICA 80	ICA 50	0.099

Connections node #1	→ node #2	GC Strength $x10^2$
ICA 65	ICA 56	0.099
ICA 91	ICA 70	0.099
ICA 88	ICA 48	0.099
ICA 24	ICA 92	0.099
ICA 87	ICA 40	0.099
ICA 21	ICA 24	0.099
ICA 83	ICA 57	0.099
ICA 51	ICA 71	0.099
ICA 71	ICA 78	0.099
ICA 99	ICA 24	0.099
ICA 57	ICA 54	0.099
ICA 84	ICA 43	0.099
ICA 87	ICA 60	0.099
ICA 94	ICA 21	0.099
ICA 9	ICA 68	0.099
ICA 86	ICA 31	0.099

Connections node #1	→ node #2	GC Strength $x10^2$
ICA 48	ICA 53	0.099
ICA 21	ICA 99	0.099
ICA 63	ICA 89	0.098
ICA 49	ICA 89	0.098
ICA 100	ICA 54	0.098
ICA 5	ICA 90	0.098
ICA 81	ICA 25	0.098
ICA 65	ICA 25	0.098
ICA 30	ICA 23	0.098
ICA 67	ICA 2	0.098
ICA 73	ICA 62	0.098
ICA 29	ICA 21	0.098
ICA 99	ICA 42	0.098
ICA 85	ICA 25	0.098
ICA 73	ICA 5	0.098
ICA 57	ICA 44	0.098

Connections node #1	→ node #2	GC Strength $x10^2$
ICA 57	ICA 51	0.098
ICA 88	ICA 51	0.098
ICA 23	ICA 35	0.098
ICA 38	ICA 45	0.098
ICA 96	ICA 40	0.098
ICA 36	ICA 50	0.098
ICA 94	ICA 71	0.098
ICA 24	ICA 37	0.098
ICA 64	ICA 29	0.098
ICA 58	ICA 48	0.098
ICA 44	ICA 46	0.098
ICA 85	ICA 58	0.098
ICA 66	ICA 62	0.098
ICA 75	ICA 80	0.098
ICA 65	ICA 32	0.098
ICA 72	ICA 94	0.098

Connections		GC
node #1 →	node #2	Strength $x10^2$
ICA 100	ICA 75	0.098
ICA 95	ICA 100	0.098
ICA 35	ICA 87	0.098
ICA 76	ICA 23	0.097
ICA 57	ICA 80	0.097
ICA 100	ICA 58	0.097
ICA 86	ICA 26	0.097
ICA 73	ICA 93	0.097
ICA 22	ICA 69	0.097
ICA 60	ICA 32	0.097
ICA 51	ICA 35	0.097
ICA 63	ICA 28	0.097
ICA 77	ICA 100	0.097
ICA 57	ICA 39	0.097
ICA 36	ICA 31	0.097
ICA 56	ICA 66	0.097

Connections		GC
node #1 →	node #2	Strength $x10^2$
ICA 56	ICA 51	0.097
ICA 51	ICA 64	0.097
ICA 85	ICA 42	0.097
ICA 62	ICA 96	0.097
ICA 59	ICA 98	0.097
ICA 88	ICA 79	0.097
ICA 89	ICA 81	0.097
ICA 100	ICA 32	0.097
ICA 83	ICA 23	0.097
ICA 85	ICA 2	0.097
ICA 26	ICA 47	0.097
ICA 98	ICA 81	0.097
ICA 55	ICA 26	0.097
ICA 89	ICA 35	0.097
ICA 70	ICA 68	0.097
ICA 84	ICA 58	0.097

Connections		GC
node #1 →	node #2	Strength $x10^2$
ICA 99	ICA 34	0.097
ICA 61	ICA 21	0.097
ICA 55	ICA 61	0.097
ICA 85	ICA 3	0.097
ICA 30	ICA 90	0.097
ICA 58	ICA 36	0.097
ICA 63	ICA 24	0.097
ICA 50	ICA 53	0.097
ICA 74	ICA 80	0.097
ICA 28	ICA 29	0.096
ICA 55	ICA 98	0.096
ICA 93	ICA 79	0.096
ICA 81	ICA 90	0.096
ICA 54	ICA 46	0.096
ICA 86	ICA 40	0.096
ICA 98	ICA 50	0.096

Connections		GC
node #1 →	node #2	Strength $x10^2$
ICA 22	ICA 50	0.096
ICA 77	ICA 63	0.096
ICA 55	ICA 3	0.096
ICA 50	ICA 40	0.096
ICA 57	ICA 95	0.096
ICA 95	ICA 57	0.096
ICA 99	ICA 88	0.096
ICA 70	ICA 35	0.096
ICA 92	ICA 37	0.096
ICA 77	ICA 55	0.096
ICA 91	ICA 76	0.096
ICA 77	ICA 79	0.096
ICA 93	ICA 48	0.096
ICA 56	ICA 90	0.096
ICA 83	ICA 91	0.096
ICA 64	ICA 45	0.096

Connections		GC
node #1 →	node #2	Strength $x10^2$
ICA 99	ICA 32	0.096
ICA 77	ICA 35	0.096
ICA 76	ICA 36	0.096
ICA 86	ICA 70	0.096
ICA 32	ICA 42	0.096
ICA 7	ICA 86	0.096
ICA 89	ICA 74	0.096
ICA 97	ICA 29	0.096
ICA 56	ICA 31	0.096
ICA 100	ICA 81	0.096
ICA 71	ICA 75	0.096
ICA 92	ICA 33	0.096
ICA 100	ICA 84	0.096
ICA 25	ICA 60	0.096
ICA 97	ICA 74	0.095
ICA 94	ICA 93	0.095

Connections		GC
node #1 →	node #2	Strength $x10^2$
ICA 92	ICA 64	0.095
ICA 4	ICA 99	0.095
ICA 91	ICA 95	0.095
ICA 97	ICA 69	0.095
ICA 93	ICA 49	0.095
ICA 98	ICA 85	0.095
ICA 94	ICA 24	0.095
ICA 67	ICA 36	0.095
ICA 89	ICA 80	0.095
ICA 78	ICA 92	0.095
ICA 71	ICA 35	0.095
ICA 51	ICA 72	0.095
ICA 37	ICA 36	0.095
ICA 57	ICA 23	0.095
ICA 82	ICA 34	0.095
ICA 65	ICA 73	0.095

Connections		GC
node #1 →	node #2	Strength $x10^2$
ICA 57	ICA 27	0.095
ICA 24	ICA 55	0.095
ICA 83	ICA 70	0.095
ICA 23	ICA 24	0.095
ICA 83	ICA 48	0.095
ICA 88	ICA 100	0.095
ICA 96	ICA 58	0.095
ICA 71	ICA 24	0.095
ICA 85	ICA 4	0.095
ICA 73	ICA 64	0.095
ICA 81	ICA 79	0.095
ICA 57	ICA 47	0.095
ICA 7	ICA 82	0.095
ICA 55	ICA 95	0.095
ICA 88	ICA 66	0.095
ICA 73	ICA 92	0.095

Connections		GC
node #1 →	node #2	Strength $x10^2$
ICA 65	ICA 35	0.095
ICA 76	ICA 88	0.095
ICA 45	ICA 46	0.095
ICA 63	ICA 56	0.095
ICA 87	ICA 46	0.095
ICA 83	ICA 60	0.095
ICA 73	ICA 95	0.095
ICA 36	ICA 52	0.095
ICA 96	ICA 59	0.095
ICA 69	ICA 72	0.095
ICA 67	ICA 9	0.095
ICA 58	ICA 57	0.095
ICA 100	ICA 35	0.095
ICA 81	ICA 68	0.095
ICA 58	ICA 27	0.095
ICA 67	ICA 50	0.095

Connections		GC
node #1 →	node #2	Strength x10^2
ICA 32	ICA 26	0.095
ICA 23	ICA 4	0.095
ICA 1	ICA 87	0.095
ICA 100	ICA 83	0.094
ICA 66	ICA 45	0.094
ICA 57	ICA 26	0.094
ICA 100	ICA 36	0.094
ICA 71	ICA 40	0.094
ICA 98	ICA 83	0.094
ICA 4	ICA 2	0.094
ICA 86	ICA 32	0.094
ICA 85	ICA 64	0.094
ICA 85	ICA 37	0.094
ICA 62	ICA 84	0.094
ICA 86	ICA 49	0.094
ICA 6	ICA 100	0.094

Connections		GC
node #1 →	node #2	Strength x10^2
ICA 92	ICA 36	0.094
ICA 95	ICA 81	0.094
ICA 25	ICA 27	0.094
ICA 89	ICA 2	0.094
ICA 94	ICA 63	0.094
ICA 64	ICA 4	0.094
ICA 86	ICA 61	0.094
ICA 68	ICA 84	0.094
ICA 95	ICA 25	0.094
ICA 48	ICA 54	0.094
ICA 98	ICA 69	0.094
ICA 80	ICA 92	0.094
ICA 87	ICA 100	0.094
ICA 62	ICA 59	0.094
ICA 50	ICA 61	0.094
ICA 77	ICA 90	0.094

Connections		GC
node #1 →	node #2	Strength x10^2
ICA 80	ICA 93	0.094
ICA 36	ICA 88	0.094
ICA 57	ICA 69	0.094
ICA 69	ICA 76	0.094
ICA 40	ICA 92	0.094
ICA 99	ICA 23	0.094
ICA 98	ICA 70	0.094
ICA 55	ICA 27	0.094
ICA 36	ICA 24	0.094
ICA 57	ICA 65	0.094
ICA 58	ICA 96	0.094
ICA 87	ICA 42	0.094
ICA 95	ICA 37	0.094
ICA 64	ICA 68	0.094
ICA 68	ICA 95	0.094
ICA 60	ICA 68	0.094

Connections		GC
node #1 →	node #2	Strength x10^2
ICA 52	ICA 43	0.094
ICA 59	ICA 93	0.094
ICA 60	ICA 93	0.094
ICA 50	ICA 36	0.093
ICA 76	ICA 82	0.093
ICA 74	ICA 86	0.093
ICA 92	ICA 68	0.093
ICA 99	ICA 87	0.093
ICA 21	ICA 61	0.093
ICA 62	ICA 56	0.093
ICA 62	ICA 75	0.093
ICA 99	ICA 97	0.093
ICA 70	ICA 87	0.093
ICA 83	ICA 50	0.093
ICA 62	ICA 83	0.093
ICA 91	ICA 79	0.093

Connections: node #1 →	node #2	GC Strength $x10^2$
ICA 80	ICA 100	0.093
ICA 86	ICA 74	0.093
ICA 90	ICA 88	0.093
ICA 100	ICA 56	0.093
ICA 94	ICA 10	0.093
ICA 94	ICA 52	0.093
ICA 58	ICA 65	0.093
ICA 24	ICA 36	0.093
ICA 36	ICA 65	0.093
ICA 63	ICA 27	0.093
ICA 76	ICA 80	0.093
ICA 95	ICA 68	0.093
ICA 62	ICA 46	0.093
ICA 29	ICA 30	0.093
ICA 100	ICA 64	0.093
ICA 87	ICA 1	0.093

Connections: node #1 →	node #2	GC Strength $x10^2$
ICA 70	ICA 26	0.093
ICA 80	ICA 78	0.093
ICA 90	ICA 22	0.093
ICA 24	ICA 22	0.093
ICA 83	ICA 77	0.093
ICA 89	ICA 28	0.093
ICA 74	ICA 96	0.093
ICA 96	ICA 60	0.093
ICA 100	ICA 70	0.092
ICA 26	ICA 64	0.092
ICA 27	ICA 24	0.092
ICA 88	ICA 92	0.092
ICA 35	ICA 60	0.092
ICA 21	ICA 98	0.092
ICA 97	ICA 1	0.092
ICA 62	ICA 51	0.092

Connections: node #1 →	node #2	GC Strength $x10^2$
ICA 92	ICA 76	0.092
ICA 92	ICA 30	0.092
ICA 90	ICA 63	0.092
ICA 84	ICA 100	0.092
ICA 92	ICA 93	0.092
ICA 95	ICA 47	0.092
ICA 21	ICA 88	0.092
ICA 66	ICA 42	0.092
ICA 46	ICA 36	0.092
ICA 94	ICA 97	0.092
ICA 83	ICA 31	0.092
ICA 96	ICA 62	0.092
ICA 74	ICA 48	0.092
ICA 64	ICA 66	0.092
ICA 94	ICA 36	0.092
ICA 62	ICA 58	0.092

Connections: node #1 →	node #2	GC Strength $x10^2$
ICA 97	ICA 88	0.092
ICA 88	ICA 78	0.092
ICA 67	ICA 91	0.092
ICA 94	ICA 47	0.092
ICA 64	ICA 98	0.092
ICA 84	ICA 73	0.092
ICA 92	ICA 99	0.092
ICA 28	ICA 97	0.092
ICA 59	ICA 21	0.092
ICA 96	ICA 98	0.092
ICA 92	ICA 52	0.092
ICA 77	ICA 98	0.092
ICA 22	ICA 92	0.092
ICA 85	ICA 30	0.092
ICA 100	ICA 76	0.092
ICA 61	ICA 72	0.092

Connections		GC
node #1 →	node #2	Strength $x10^2$
ICA 82	ICA 50	0.092
ICA 81	ICA 78	0.092
ICA 87	ICA 43	0.092
ICA 57	ICA 78	0.092
ICA 100	ICA 92	0.092
ICA 30	ICA 27	0.092
ICA 67	ICA 78	0.092
ICA 56	ICA 81	0.092
ICA 58	ICA 100	0.092
ICA 61	ICA 45	0.091
ICA 91	ICA 27	0.091
ICA 56	ICA 71	0.091
ICA 69	ICA 95	0.091
ICA 83	ICA 5	0.091
ICA 4	ICA 23	0.091
ICA 95	ICA 46	0.091

Connections		GC
node #1 →	node #2	Strength $x10^2$
ICA 81	ICA 57	0.091
ICA 68	ICA 90	0.091
ICA 28	ICA 68	0.091
ICA 48	ICA 44	0.091
ICA 90	ICA 64	0.091
ICA 91	ICA 69	0.091
ICA 59	ICA 31	0.091
ICA 95	ICA 49	0.091
ICA 74	ICA 26	0.091
ICA 65	ICA 71	0.091
ICA 81	ICA 59	0.091
ICA 23	ICA 43	0.091
ICA 58	ICA 52	0.091
ICA 89	ICA 49	0.091
ICA 47	ICA 26	0.091
ICA 28	ICA 22	0.091

Connections		GC
node #1 →	node #2	Strength $x10^2$
ICA 47	ICA 52	0.091
ICA 1	ICA 26	0.091
ICA 77	ICA 34	0.091
ICA 59	ICA 82	0.091
ICA 81	ICA 67	0.091
ICA 63	ICA 94	0.091
ICA 33	ICA 61	0.091
ICA 64	ICA 61	0.091
ICA 90	ICA 94	0.091
ICA 56	ICA 36	0.091
ICA 62	ICA 78	0.091
ICA 88	ICA 76	0.091
ICA 96	ICA 90	0.091
ICA 80	ICA 30	0.091
ICA 59	ICA 79	0.091
ICA 73	ICA 85	0.091

Connections		GC
node #1 →	node #2	Strength $x10^2$
ICA 31	ICA 29	0.091
ICA 57	ICA 99	0.091
ICA 91	ICA 38	0.091
ICA 89	ICA 70	0.091
ICA 82	ICA 37	0.091
ICA 96	ICA 83	0.091
ICA 38	ICA 100	0.091
ICA 57	ICA 48	0.091
ICA 53	ICA 50	0.091
ICA 87	ICA 73	0.091
ICA 61	ICA 70	0.091
ICA 45	ICA 67	0.091
ICA 94	ICA 80	0.090
ICA 99	ICA 37	0.090
ICA 81	ICA 97	0.090
ICA 89	ICA 37	0.090

Connections node #1	→ node #2	GC Strength $x10^2$
ICA 91	ICA 80	0.090
ICA 49	ICA 46	0.090
ICA 91	ICA 97	0.090
ICA 3	ICA 97	0.090
ICA 24	ICA 38	0.090
ICA 55	ICA 34	0.090
ICA 98	ICA 74	0.090
ICA 59	ICA 24	0.090
ICA 81	ICA 84	0.090
ICA 50	ICA 59	0.090
ICA 62	ICA 31	0.090
ICA 36	ICA 40	0.090
ICA 66	ICA 26	0.090
ICA 56	ICA 27	0.090
ICA 40	ICA 50	0.090
ICA 39	ICA 89	0.090

Connections node #1	→ node #2	GC Strength $x10^2$
ICA 24	ICA 52	0.090
ICA 100	ICA 95	0.090
ICA 56	ICA 89	0.090
ICA 52	ICA 57	0.090
ICA 73	ICA 4	0.090
ICA 77	ICA 91	0.090
ICA 89	ICA 29	0.090
ICA 59	ICA 34	0.090
ICA 63	ICA 51	0.090
ICA 90	ICA 33	0.090
ICA 98	ICA 28	0.090
ICA 84	ICA 79	0.090
ICA 95	ICA 91	0.090
ICA 60	ICA 27	0.090
ICA 69	ICA 92	0.090
ICA 73	ICA 96	0.090

Connections node #1	→ node #2	GC Strength $x10^2$
ICA 89	ICA 30	0.090
ICA 100	ICA 50	0.090
ICA 26	ICA 25	0.090
ICA 74	ICA 23	0.090
ICA 77	ICA 49	0.090
ICA 62	ICA 24	0.090
ICA 81	ICA 100	0.090
ICA 27	ICA 30	0.090
ICA 98	ICA 59	0.090
ICA 82	ICA 98	0.090
ICA 63	ICA 37	0.090
ICA 93	ICA 7	0.090
ICA 73	ICA 90	0.090
ICA 56	ICA 63	0.090
ICA 68	ICA 51	0.090
ICA 79	ICA 73	0.090

Connections node #1	→ node #2	GC Strength $x10^2$
ICA 77	ICA 82	0.090
ICA 79	ICA 72	0.090
ICA 61	ICA 22	0.090
ICA 85	ICA 50	0.090
ICA 83	ICA 74	0.090
ICA 73	ICA 86	0.090
ICA 57	ICA 45	0.090
ICA 95	ICA 22	0.090
ICA 85	ICA 97	0.090
ICA 94	ICA 81	0.090
ICA 98	ICA 35	0.089
ICA 36	ICA 75	0.089
ICA 77	ICA 72	0.089
ICA 65	ICA 29	0.089
ICA 81	ICA 94	0.089
ICA 99	ICA 4	0.089

Connections node #1 →	node #2	GC Strength $x10^2$	Connections node #1 →	node #2	GC Strength $x10^2$	Connections node #1 →	node #2	GC Strength $x10^2$	Connections node #1 →	node #2	GC Strength $x10^2$
ICA 74	ICA 82	0.089	ICA 93	ICA 39	0.089	ICA 83	ICA 68	0.089	ICA 66	ICA 51	0.089
ICA 7	ICA 100	0.089	ICA 91	ICA 48	0.089	ICA 67	ICA 48	0.089	ICA 56	ICA 28	0.089
ICA 37	ICA 24	0.089	ICA 86	ICA 27	0.089	ICA 68	ICA 60	0.089	ICA 92	ICA 40	0.088
ICA 55	ICA 94	0.089	ICA 67	ICA 71	0.089	ICA 64	ICA 30	0.089	ICA 87	ICA 94	0.088
ICA 93	ICA 76	0.089	ICA 38	ICA 43	0.089	ICA 84	ICA 37	0.089	ICA 63	ICA 73	0.088
ICA 31	ICA 37	0.089	ICA 82	ICA 67	0.089	ICA 93	ICA 82	0.089	ICA 88	ICA 70	0.088
ICA 72	ICA 61	0.089	ICA 66	ICA 81	0.089	ICA 60	ICA 72	0.089	ICA 57	ICA 81	0.088
ICA 64	ICA 7	0.089	ICA 57	ICA 91	0.089	ICA 3	ICA 90	0.089	ICA 32	ICA 39	0.088
ICA 56	ICA 93	0.089	ICA 60	ICA 98	0.089	ICA 88	ICA 3	0.089	ICA 71	ICA 98	0.088
ICA 91	ICA 26	0.089	ICA 89	ICA 97	0.089	ICA 58	ICA 93	0.089	ICA 10	ICA 85	0.088
ICA 1	ICA 24	0.089	ICA 92	ICA 95	0.089	ICA 6	ICA 62	0.089	ICA 58	ICA 94	0.088
ICA 93	ICA 74	0.089	ICA 81	ICA 62	0.089	ICA 64	ICA 31	0.089	ICA 93	ICA 71	0.088
ICA 2	ICA 79	0.089	ICA 67	ICA 77	0.089	ICA 60	ICA 58	0.089	ICA 85	ICA 51	0.088
ICA 82	ICA 35	0.089	ICA 99	ICA 92	0.089	ICA 81	ICA 93	0.089	ICA 29	ICA 90	0.088
ICA 77	ICA 24	0.089	ICA 100	ICA 68	0.089	ICA 54	ICA 49	0.089	ICA 21	ICA 22	0.088
ICA 98	ICA 26	0.089	ICA 38	ICA 26	0.089	ICA 45	ICA 52	0.089	ICA 93	ICA 40	0.088

Connections		GC
node #1 →	node #2	Strength $x10^2$
ICA 48	ICA 45	0.087
ICA 100	ICA 93	0.087
ICA 74	ICA 98	0.087
ICA 95	ICA 98	0.087
ICA 18	ICA 79	0.087
ICA 84	ICA 24	0.087
ICA 2	ICA 99	0.087
ICA 91	ICA 55	0.087
ICA 58	ICA 23	0.087
ICA 61	ICA 87	0.087
ICA 56	ICA 1	0.087
ICA 93	ICA 38	0.087
ICA 92	ICA 77	0.087
ICA 84	ICA 92	0.087
ICA 98	ICA 58	0.087
ICA 61	ICA 91	0.087

Connections		GC
node #1 →	node #2	Strength $x10^2$
ICA 74	ICA 5	0.087
ICA 94	ICA 85	0.087
ICA 29	ICA 38	0.087
ICA 61	ICA 73	0.087
ICA 90	ICA 66	0.087
ICA 66	ICA 52	0.087
ICA 74	ICA 94	0.087
ICA 84	ICA 76	0.086
ICA 79	ICA 92	0.086
ICA 55	ICA 43	0.086
ICA 64	ICA 25	0.086
ICA 56	ICA 77	0.086
ICA 98	ICA 33	0.086
ICA 95	ICA 92	0.086
ICA 44	ICA 23	0.086
ICA 83	ICA 80	0.086

Connections		GC
node #1 →	node #2	Strength $x10^2$
ICA 63	ICA 90	0.086
ICA 36	ICA 49	0.086
ICA 48	ICA 81	0.086
ICA 35	ICA 85	0.086
ICA 84	ICA 39	0.086
ICA 47	ICA 38	0.086
ICA 59	ICA 89	0.086
ICA 85	ICA 38	0.086
ICA 88	ICA 99	0.086
ICA 66	ICA 54	0.086
ICA 70	ICA 65	0.086
ICA 92	ICA 43	0.086
ICA 91	ICA 100	0.086
ICA 50	ICA 93	0.086
ICA 66	ICA 76	0.086
ICA 93	ICA 56	0.086

Connections		GC
node #1 →	node #2	Strength $x10^2$
ICA 73	ICA 56	0.086
ICA 61	ICA 26	0.086
ICA 81	ICA 56	0.086
ICA 65	ICA 86	0.086
ICA 83	ICA 69	0.086
ICA 6	ICA 50	0.086
ICA 67	ICA 70	0.086
ICA 5	ICA 24	0.086
ICA 50	ICA 58	0.086
ICA 36	ICA 41	0.086
ICA 80	ICA 38	0.086
ICA 90	ICA 24	0.086
ICA 95	ICA 43	0.086
ICA 35	ICA 73	0.086
ICA 60	ICA 22	0.086
ICA 35	ICA 77	0.086

Connections		GC
node #1 →	node #2	Strength x10^2
ICA 57	ICA 70	0.087
ICA 91	ICA 49	0.087
ICA 63	ICA 82	0.087
ICA 97	ICA 45	0.087
ICA 70	ICA 58	0.087
ICA 33	ICA 21	0.087
ICA 70	ICA 57	0.087
ICA 65	ICA 75	0.087
ICA 87	ICA 3	0.087
ICA 3	ICA 23	0.087
ICA 87	ICA 96	0.087
ICA 76	ICA 77	0.087
ICA 99	ICA 73	0.087
ICA 88	ICA 87	0.087
ICA 66	ICA 95	0.087
ICA 77	ICA 86	0.087

Connections		GC
node #1 →	node #2	Strength x10^2
ICA 93	ICA 23	0.087
ICA 97	ICA 99	0.087
ICA 86	ICA 72	0.087
ICA 6	ICA 68	0.086
ICA 6	ICA 37	0.086
ICA 55	ICA 91	0.086
ICA 90	ICA 78	0.086
ICA 51	ICA 62	0.086
ICA 73	ICA 82	0.086
ICA 64	ICA 35	0.086
ICA 83	ICA 22	0.086
ICA 3	ICA 87	0.086
ICA 64	ICA 83	0.086
ICA 37	ICA 67	0.086
ICA 57	ICA 62	0.086
ICA 65	ICA 6	0.086

Connections		GC
node #1 →	node #2	Strength x10^2
ICA 58	ICA 98	0.086
ICA 5	ICA 7	0.086
ICA 85	ICA 52	0.086
ICA 65	ICA 3	0.086
ICA 45	ICA 64	0.086
ICA 59	ICA 87	0.086
ICA 83	ICA 42	0.086
ICA 98	ICA 46	0.086
ICA 22	ICA 21	0.086
ICA 96	ICA 93	0.086
ICA 2	ICA 29	0.086
ICA 55	ICA 84	0.086
ICA 76	ICA 25	0.086
ICA 97	ICA 10	0.086
ICA 59	ICA 22	0.086
ICA 96	ICA 84	0.086

Connections		GC
node #1 →	node #2	Strength x10^2
ICA 67	ICA 76	0.086
ICA 82	ICA 77	0.086
ICA 91	ICA 63	0.086
ICA 70	ICA 36	0.086
ICA 95	ICA 31	0.086
ICA 32	ICA 27	0.086
ICA 61	ICA 81	0.086
ICA 93	ICA 68	0.086
ICA 88	ICA 38	0.086
ICA 57	ICA 22	0.086
ICA 56	ICA 37	0.086
ICA 22	ICA 23	0.086
ICA 29	ICA 27	0.086
ICA 95	ICA 72	0.086
ICA 58	ICA 24	0.086
ICA 85	ICA 98	0.086

Connections		GC
node #1 →	node #2	Strength x10^2
ICA 45	ICA 53	0.086
ICA 100	ICA 4	0.086
ICA 71	ICA 25	0.086
ICA 62	ICA 60	0.086
ICA 66	ICA 71	0.086
ICA 63	ICA 22	0.086
ICA 74	ICA 27	0.086
ICA 71	ICA 47	0.086
ICA 64	ICA 96	0.086
ICA 63	ICA 52	0.086
ICA 66	ICA 32	0.086
ICA 65	ICA 21	0.086
ICA 22	ICA 57	0.086
ICA 66	ICA 97	0.086
ICA 28	ICA 89	0.086
ICA 22	ICA 28	0.086

Connections		GC
node #1 →	node #2	Strength x10^2
ICA 26	ICA 34	0.086
ICA 65	ICA 26	0.086
ICA 57	ICA 64	0.086
ICA 90	ICA 32	0.086
ICA 66	ICA 92	0.086
ICA 90	ICA 52	0.086
ICA 92	ICA 75	0.086
ICA 6	ICA 61	0.086
ICA 4	ICA 76	0.086
ICA 2	ICA 25	0.086
ICA 25	ICA 23	0.086
ICA 60	ICA 64	0.086
ICA 55	ICA 76	0.086
ICA 84	ICA 78	0.086
ICA 24	ICA 48	0.086
ICA 73	ICA 45	0.086

Connections		GC
node #1 →	node #2	Strength x10^2
ICA 22	ICA 68	0.086
ICA 35	ICA 46	0.086
ICA 65	ICA 52	0.086
ICA 60	ICA 76	0.085
ICA 36	ICA 28	0.085
ICA 34	ICA 36	0.085
ICA 64	ICA 27	0.085
ICA 86	ICA 57	0.085
ICA 96	ICA 49	0.085
ICA 21	ICA 89	0.085
ICA 59	ICA 43	0.085
ICA 33	ICA 88	0.085
ICA 64	ICA 32	0.085
ICA 50	ICA 84	0.085
ICA 90	ICA 30	0.085
ICA 96	ICA 24	0.085

Connections		GC
node #1 →	node #2	Strength x10^2
ICA 95	ICA 28	0.085
ICA 85	ICA 78	0.085
ICA 45	ICA 85	0.085
ICA 67	ICA 54	0.085
ICA 89	ICA 51	0.085
ICA 6	ICA 84	0.085
ICA 78	ICA 80	0.085
ICA 61	ICA 55	0.085
ICA 88	ICA 23	0.085
ICA 77	ICA 93	0.085
ICA 97	ICA 94	0.085
ICA 7	ICA 59	0.085
ICA 96	ICA 6	0.085
ICA 91	ICA 98	0.085
ICA 87	ICA 75	0.085
ICA 97	ICA 89	0.085

Connections		GC
node #1 →	node #2	Strength $x10^2$
ICA 46	ICA 45	0.085
ICA 35	ICA 36	0.085
ICA 26	ICA 28	0.085
ICA 23	ICA 37	0.085
ICA 62	ICA 66	0.085
ICA 88	ICA 71	0.085
ICA 28	ICA 47	0.085
ICA 22	ICA 59	0.085
ICA 68	ICA 62	0.085
ICA 23	ICA 39	0.085
ICA 65	ICA 8	0.085
ICA 96	ICA 73	0.085
ICA 57	ICA 50	0.085
ICA 71	ICA 26	0.085
ICA 33	ICA 90	0.085
ICA 80	ICA 99	0.085

Connections		GC
node #1 →	node #2	Strength $x10^2$
ICA 90	ICA 50	0.085
ICA 94	ICA 86	0.085
ICA 56	ICA 84	0.085
ICA 29	ICA 84	0.085
ICA 98	ICA 27	0.085
ICA 87	ICA 37	0.085
ICA 88	ICA 50	0.085
ICA 99	ICA 74	0.085
ICA 23	ICA 44	0.085
ICA 35	ICA 56	0.085
ICA 45	ICA 48	0.085
ICA 84	ICA 99	0.085
ICA 90	ICA 39	0.085
ICA 51	ICA 70	0.085
ICA 32	ICA 100	0.085
ICA 74	ICA 2	0.085

Connections		GC
node #1 →	node #2	Strength $x10^2$
ICA 88	ICA 93	0.085
ICA 71	ICA 33	0.085
ICA 74	ICA 24	0.085
ICA 84	ICA 59	0.085
ICA 91	ICA 61	0.085
ICA 83	ICA 89	0.085
ICA 28	ICA 31	0.085
ICA 81	ICA 45	0.085
ICA 62	ICA 81	0.085
ICA 95	ICA 42	0.085
ICA 4	ICA 87	0.085
ICA 78	ICA 57	0.085
ICA 89	ICA 64	0.084
ICA 86	ICA 89	0.084
ICA 50	ICA 97	0.084
ICA 74	ICA 91	0.084

Connections		GC
node #1 →	node #2	Strength $x10^2$
ICA 32	ICA 71	0.084
ICA 92	ICA 27	0.084
ICA 92	ICA 73	0.084
ICA 75	ICA 81	0.084
ICA 85	ICA 96	0.084
ICA 23	ICA 2	0.084
ICA 71	ICA 22	0.084
ICA 77	ICA 29	0.084
ICA 73	ICA 28	0.084
ICA 88	ICA 84	0.084
ICA 56	ICA 52	0.084
ICA 73	ICA 60	0.084
ICA 31	ICA 92	0.084
ICA 77	ICA 1	0.084
ICA 88	ICA 74	0.084
ICA 77	ICA 75	0.084

Connections: node #1 →	node #2	GC: Strength $x10^2$
ICA 59	ICA 78	0.084
ICA 95	ICA 64	0.084
ICA 60	ICA 49	0.084
ICA 99	ICA 85	0.084
ICA 42	ICA 85	0.084
ICA 62	ICA 57	0.084
ICA 84	ICA 55	0.084
ICA 70	ICA 84	0.084
ICA 12	ICA 67	0.084
ICA 65	ICA 48	0.084
ICA 93	ICA 1	0.084
ICA 48	ICA 63	0.084
ICA 79	ICA 82	0.084
ICA 36	ICA 25	0.084
ICA 73	ICA 31	0.084
ICA 4	ICA 10	0.084

Connections: node #1 →	node #2	GC: Strength $x10^2$
ICA 100	ICA 25	0.084
ICA 80	ICA 39	0.084
ICA 61	ICA 43	0.084
ICA 58	ICA 62	0.084
ICA 24	ICA 61	0.084
ICA 87	ICA 71	0.084
ICA 76	ICA 55	0.084
ICA 79	ICA 81	0.084
ICA 66	ICA 69	0.084
ICA 85	ICA 5	0.084
ICA 71	ICA 23	0.084
ICA 27	ICA 73	0.084
ICA 88	ICA 1	0.084
ICA 63	ICA 71	0.084
ICA 32	ICA 24	0.084
ICA 91	ICA 89	0.084

Connections: node #1 →	node #2	GC: Strength $x10^2$
ICA 92	ICA 66	0.084
ICA 60	ICA 28	0.084
ICA 33	ICA 54	0.084
ICA 76	ICA 27	0.084
ICA 58	ICA 21	0.084
ICA 62	ICA 26	0.084
ICA 91	ICA 46	0.084
ICA 62	ICA 22	0.084
ICA 97	ICA 93	0.084
ICA 70	ICA 94	0.084
ICA 64	ICA 80	0.084
ICA 75	ICA 73	0.084
ICA 74	ICA 41	0.084
ICA 84	ICA 45	0.084
ICA 63	ICA 100	0.084
ICA 72	ICA 26	0.084

Connections: node #1 →	node #2	GC: Strength $x10^2$
ICA 95	ICA 4	0.084
ICA 91	ICA 37	0.084
ICA 67	ICA 55	0.084
ICA 94	ICA 87	0.084
ICA 100	ICA 33	0.084
ICA 88	ICA 90	0.084
ICA 59	ICA 88	0.084
ICA 61	ICA 35	0.084
ICA 76	ICA 95	0.084
ICA 98	ICA 24	0.084
ICA 47	ICA 82	0.084
ICA 40	ICA 37	0.084
ICA 98	ICA 84	0.084
ICA 70	ICA 95	0.084
ICA 22	ICA 63	0.084
ICA 94	ICA 66	0.084

Connections		GC
node #1 →	node #2	Strength $x10^2$
ICA 100	ICA 85	0.083
ICA 100	ICA 1	0.083
ICA 66	ICA 39	0.083
ICA 62	ICA 74	0.083
ICA 59	ICA 25	0.083
ICA 85	ICA 24	0.083
ICA 90	ICA 27	0.083
ICA 67	ICA 57	0.083
ICA 97	ICA 5	0.083
ICA 47	ICA 69	0.083
ICA 63	ICA 59	0.083
ICA 78	ICA 77	0.083
ICA 3	ICA 22	0.083
ICA 33	ICA 89	0.083
ICA 58	ICA 34	0.083
ICA 93	ICA 51	0.083

Connections		GC
node #1 →	node #2	Strength $x10^2$
ICA 57	ICA 66	0.083
ICA 74	ICA 65	0.083
ICA 76	ICA 63	0.083
ICA 40	ICA 93	0.083
ICA 58	ICA 80	0.083
ICA 8	ICA 2	0.083
ICA 94	ICA 55	0.083
ICA 68	ICA 23	0.083
ICA 7	ICA 36	0.083
ICA 58	ICA 69	0.083
ICA 99	ICA 40	0.083
ICA 68	ICA 100	0.083
ICA 73	ICA 2	0.083
ICA 21	ICA 31	0.083
ICA 76	ICA 94	0.083
ICA 59	ICA 4	0.083

Connections		GC
node #1 →	node #2	Strength $x10^2$
ICA 48	ICA 55	0.083
ICA 99	ICA 29	0.083
ICA 57	ICA 35	0.083
ICA 96	ICA 42	0.083
ICA 99	ICA 94	0.083
ICA 61	ICA 66	0.083
ICA 5	ICA 23	0.083
ICA 32	ICA 63	0.083
ICA 84	ICA 61	0.083
ICA 21	ICA 58	0.083
ICA 12	ICA 97	0.083
ICA 27	ICA 72	0.083
ICA 64	ICA 23	0.083
ICA 3	ICA 59	0.083
ICA 86	ICA 38	0.083
ICA 83	ICA 38	0.083

Connections		GC
node #1 →	node #2	Strength $x10^2$
ICA 93	ICA 69	0.083
ICA 73	ICA 84	0.083
ICA 84	ICA 34	0.083
ICA 68	ICA 87	0.083
ICA 98	ICA 47	0.083
ICA 60	ICA 42	0.083
ICA 63	ICA 40	0.083
ICA 98	ICA 95	0.083
ICA 87	ICA 24	0.083
ICA 50	ICA 51	0.083
ICA 97	ICA 80	0.083
ICA 38	ICA 51	0.083
ICA 28	ICA 57	0.083
ICA 65	ICA 50	0.083
ICA 74	ICA 90	0.083
ICA 62	ICA 36	0.083

Connections		GC
node #1	node #2	Strength $x10^2$
**ICA 59** 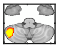	**ICA 50** 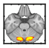	0.083
**ICA 64** 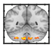	**ICA 72** 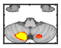	0.083
**ICA 100** 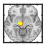	**ICA 87** 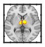	0.083
**ICA 40** 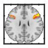	**ICA 88** 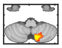	0.083
